# Pros and cons of methylation-based enrichment methods for ancient DNA

**DOI:** 10.1038/srep11826

**Published:** 2015-07-02

**Authors:** Andaine Seguin-Orlando, Cristina Gamba, Clio Der Sarkissian, Luca Ermini, Guillaume Louvel, Eugenia Boulygina, Alexey Sokolov, Artem Nedoluzhko, Eline D. Lorenzen, Patricio Lopez, H. Gregory McDonald, Eric Scott, Alexei Tikhonov, Thomas W. Stafford,, Ahmed H. Alfarhan, Saleh A. Alquraishi, Khaled A. S. Al-Rasheid, Beth Shapiro, Eske Willerslev, Egor Prokhortchouk, Ludovic Orlando

**Affiliations:** 1 Centre for GeoGenetics, Natural History Museum of Denmark, Øster Voldgade 5-7, 1350K Copenhagen, Denmark; 2 National High-throughput DNA Sequencing Centre, Øster Farimagsgade 2D, 1353K Copenhagen, Denmark; 3 National Research Centre Kurchatov Institute, 1, Akademika Kurchatova, Moscow, 123182, Russian Federation; 4 Centre Bioengineering, Russian Academy of Sciences, Prospekt 60-Letiya Oktyabrya 7/1, Moscow, 117312, Russian Federation; 5 Department of Integrative Biology, University of California, Berkeley, CA 94720, USA; 6 Department of Anthropology, Universidad de Chile, Ignacio Carrera Pinto 1045, Ñuñoa, Santiago, Chile; 7 Park Museum Management Program, National Park Service, 1201 Oakridge Drive, Suite 150, Fort Collins, Colorado 80525, USA; 8 San Bernardino County Museum, Division of Geological Sciences, 2024 Orange Tree Lane, Redlands, California 92374, USA; 9 Zoological Institute of Russian Academy of Sciences, 199034 St. Petersburg, Russian Federation; 10 Institute of Applied Ecology of the North, North-Eastern Federal University, 677980 Yakutsk, Russian Federation; 11 Zoology Department, College of Science, King Saud University, Riyadh 11451, Saudi Arabia; 12 Department of Ecology and Evolutionary Biology, University of California Santa Cruz, Santa Cruz, CA 95060, USA; 13 Université de Toulouse, University Paul Sabatier (UPS), Laboratoire AMIS, CNRS UMR 5288, 37 allées Jules Guesde, 31000 Toulouse, France

## Abstract

The recent discovery that DNA methylation survives in fossil material provides an opportunity for novel molecular approaches in palaeogenomics. Here, we apply to ancient DNA extracts the probe-independent Methylated Binding Domains (MBD)-based enrichment method, which targets DNA molecules containing methylated CpGs. Using remains of a Palaeo-Eskimo Saqqaq individual, woolly mammoths, polar bears and two equine species, we confirm that DNA methylation survives in a variety of tissues, environmental contexts and over a large temporal range (4,000 to over 45,000 years before present). MBD enrichment, however, appears principally biased towards the recovery of CpG-rich and long DNA templates and is limited by the fast *post-mortem* cytosine deamination rates of methylated epialleles. This method, thus, appears only appropriate for the analysis of ancient methylomes from very well preserved samples, where both DNA fragmentation and deamination have been limited. This work represents an essential step toward the characterization of ancient methylation signatures, which will help understanding the role of epigenetic changes in past environmental and cultural transitions.

The retrieval of DNA from archaeological and palaeontological material provides direct genetic information about the evolutionary past^1^,^2^. However, DNA fragmentation and chemical damage start shortly after an organism dies, making ancient DNA (aDNA) analyses particularly challenging. Many technologies have been developed to improve the characterization of aDNA since the early days of bacterial cloning^3^, which, together with the development of High-Throughput Sequencing (HTS) technologies, opened access to an increasing number of ancient genomes^3^,^4^. The extraordinary throughput of HTS technologies is, however, often hampered by the abundance of environmental microbial DNA in aDNA extracts^5^ and shotgun sequencing can only achieve high-coverage genomes from exceptionally well-preserved material where microbial colonization is minimal (e.g.^6^,^7^,^8^).

Several target enrichment methods have been developed to circumvent these limitations. Most are based on the hybridization of probes tiled on markers of interest to DNA library templates^9^,^10^. Within a single enrichment experiment, the number of *loci* targeted can vary from the mitochondrial genome and a few nuclear genes^11^ to entire bacterial genomes^12^, human chromosomes^13^ or complete mammalian genomes^10^,^14^. Hybridization-based target enrichment approaches are subject to a variety of experimental biases, including the possible over-representation of long inserts^15^, %GC-rich genomic regions^16^ and repetitive elements^14^. Additionally, as ancient DNA library inserts are generally short, the size of adapters can influence the enrichment efficiency^17^. The number of mismatches between probes and library molecules, which increases with the evolutionary distance and the amount of *post-mortem* DNA damage, can also reduce annealing efficiency, which can represent a problem in situations where no closely related species are available for designing probes^18^.

Probe-free enrichment methods have been developed to avoid introducing bias while annealing probes to targets. With the ‘uracil selection’ method, ancient DNA templates carrying damage (in the form of deaminated cytosines) can be separated from those showing no damage during library preparation^10^. Deep sequencing of the damage-enriched fraction has shown enrichment of 1.1–4.7-fold on a range of Neanderthal specimens, and performance is expected to increase with damage levels^10^.

Methyl Binding Domains (MBD) based enrichment is another probe-free enrichment method but directly operates on DNA extracts, prior to library construction. It exploits the affinity of MBD for methylated CpG dinucleotides (^m^CpG) so as to separate complex metagenomic assemblages into two fractions^19^, the first of which binds to MBD and is mostly of vertebrate origin, while the second does not bind and is mostly microbial^19^. This is so because vertebrate genomes are globally methylated, with 70–80% of methylated cytosines being located in a CpG context in the human genome^20^, in contrast to bacterial genomes where methylation marks are often found in non-CpG contexts^21^. This method could provide the basis for new enrichment approach in aDNA research, where microbial DNA often represents the vast majority of the DNA templates extracted. Surprisingly, it has so far been used in only a single aDNA study, which focused on the analysis of 200–2,800 year-old barley seeds from a single archaeological site in southern Egypt^22^. The potential of the method, thus, still remains largely unexplored.

Here, we applied MBD enrichment to a selection of mammalian fossil specimens preserved across a wide geographical and temporal range. We identify experimental conditions that influence the overall efficiency of MBD enrichment and conclude that the method is only compatible with a cost-effective characterization of ancient methylomes when DNA fragmentation and deamination are limited.

## Results

The MBD enrichment procedure tested in this study relies on the affinity of MBD2−Fc for ^m^CpGs. The MBD2−Fc protein corresponds to the fusion of the human MBD2 domain and the Fc tail fragment of human IgG1^19^. Following incubation, ^m^CpG-rich DNA molecules bound to MDB2−Fc can be recovered using paramagnetic beads coated with the protein A, which bind to the Fc tail. The supernatant (MBD−) is expected to contain mostly DNA molecules showing low ^m^CpG content in contrast to the enriched fraction (MBD+). We explored the performance of this MBD enrichment system using 20 aDNA extracts from soft and calcified tissues (Table 1; Supplementary Table S1), including specimens preserved in a variety of environments: seven from arctic regions (the Palaeo-Eskimo Saqqaq individual from Greenland that was previously genome sequenced^6^, two Siberian woolly mammoths, three polar bears, and one and three equids from Canada and Russia, respectively), and four equids from a cave, karstic formations and a submerged terrestrial site. These specimens spanned a wide temporal range, extending from the Holocene (~4,000 years before present, BP) to the Late Pleistocene (>45,000 years BP). Following MBD enrichment, both MBD+ and MBD− fractions were used to construct DNA libraries and were deep-sequenced to generate a total of 409 million paired-end sequences on Illumina HiSeq 2500 and MiSeq platforms (Supplementary Table S3.7).

### Endogenous contents

**Figure 1:**
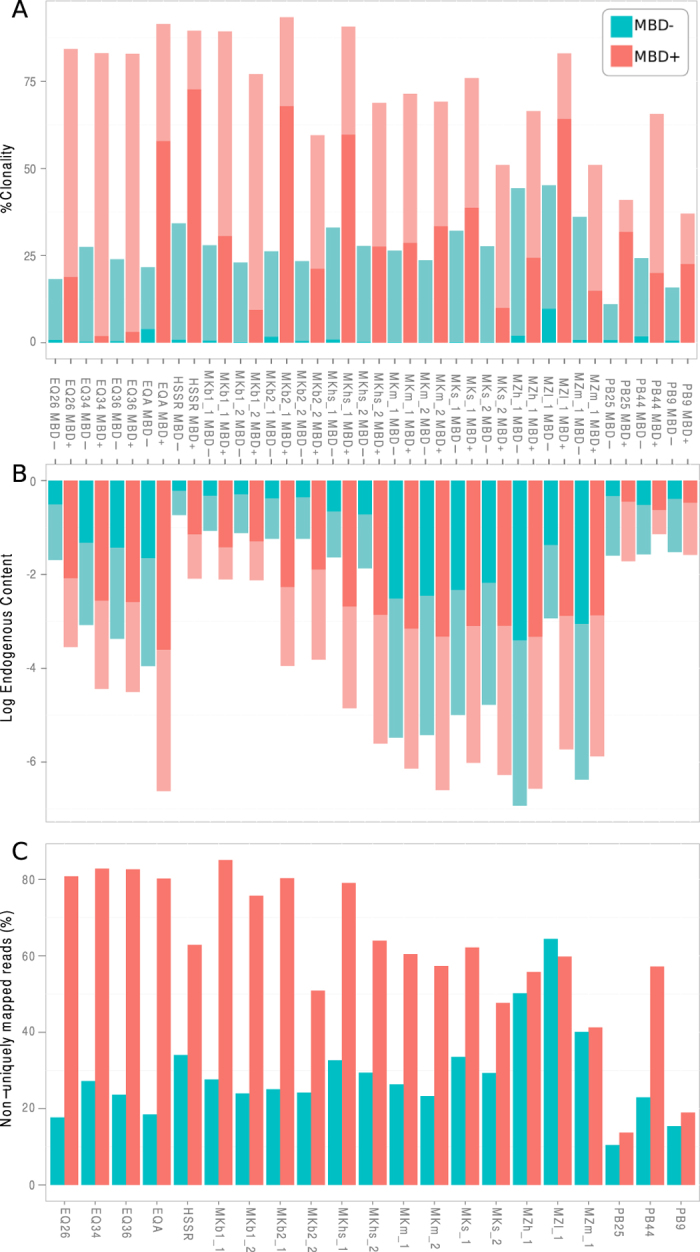
Endogenous content, clonality and number of non-unique reads of DNA libraries constructed from MBD+ and MBD− fractions. (**A**) Clonality estimates, expressed as the percentage of hits that were PCR duplicates both for those not filtered for mapping quality (MQ0, darker shade) and those filtered for mapping quality (MQ25, lighter shade). (**B**) Endogenous DNA content, expressed as the natural logarithm of the fractions of unique hits over the total number of reads retained both for those not filtered for mapping quality (MQ0, lighter shade) and those filtered for mapping quality (MQ25, darker shade). (**C**) Fraction of nuclear DNA reads non-uniquely mapped to the reference genome calculated on the full-length reads (minimum 2,000 per sample) not filtered for mapping quality (MQ0). MBD+ = MBD enriched fraction. MBD− = uncaptured fraction.

Since *post-mortem* deamination progressively transforms ^m^CpGs into TpGs, we first evaluated whether sufficient amounts of ^m^CpGs were preserved to enable successful MBD enrichment. We were able to build Illumina DNA libraries from both the MBD+ and MBD− fractions for all samples investigated. After Illumina sequencing, we used stringent alignment parameters and quantified the proportion of molecules of endogenous origin based on the number of both low and high-quality hits (hereafter referred to as MQ0 and MQ25 filters) that mapped uniquely to the reference genomes (for extinct species, genomes of phylogenetically-close species). The MQ0 filter allowed us to quantify the fraction of the sequences that aligned against repetitive elements while the MQ25 filter identified high-quality unique hits^23^. For almost all comparisons (90%), the proportion of unique endogenous reads in the MBD+ fraction was lower than that in the MBD− fraction (Fig. 1B). This represented only a 1.2–1.3-fold reduction for the three polar bear specimens, but the reduction reached about two orders of magnitude in some of the equine and mammoth specimens (90–138-fold for MQ0 and MQ25 filters, respectively; Supplementary Table S2.1). The reduction of the proportion of endogenous reads could be partly explained by an increase in the proportion of PCR duplicates in MBD+ libraries (relative to MBD− libraries), which was somewhat comparable to the reduction observed in endogenous contents (6 to 391-fold) (Fig. 1A, Supplementary Table S2.2).

### MBD enrichment efficiency

In contrast to the nuclear genome, the mitochondrial genome is globally hypo-methylated, if at all^24^. We exploited this to evaluate the efficiency of MBD enrichment. To avoid biasing our estimates, only specimens where both MBD+ and MBD− fractions had a sufficient number of mitochondrial unique hits were considered (MQ25, and ≥20 hits). As expected, the contribution of mitochondrial DNA (mtDNA) to the pool of endogenous sequences was found to decrease significantly in the MBD+ fractions relative to the MBD− fractions (Supplementary Table S2.3). This was observed across all tissues, but most dramatically for one polar bear bone (PB25) and two mammoth tissues (heart - MZh_1, muscle - Mzm_1), which showed a 15.6-fold to 23.6-fold reduction (Supplementary Table S2.3).

Similarly, using CpG island (CGI) annotations available for the human and horse reference genomes, we found that, in the MBD+ fraction, CGIs were on average 9.3-fold less covered than the rest of the genome (MQ25 filter; Supplementary Table S3.1). This is in line with the overall hypo-methylation state of CGIs^25^.

Since methylation is involved in the silencing of transposable elements (TEs), we also tested for differences in the representation of TEs between MBD+ and MBD− fractions. We found that sub-optimal hits (i.e., mapping to multiple genomic regions identified using the MQ0 filter) represented a larger proportion of endogenous reads in the MBD+ fraction than in the MBD− fraction (average = 2.4-fold increase, Supplementary Table S3.2). To confirm that this was driven by TEs, which are present in multiple copies within the genome, we stratified coverage differences for annotated TEs in MBD+ and MBD− fractions. We found a higher abundance of TEs in the MBD+ fraction, which were on average 2.1-fold more abundant than in the MBD− fraction (Supplementary Table S3.3).

**Figure 2:**
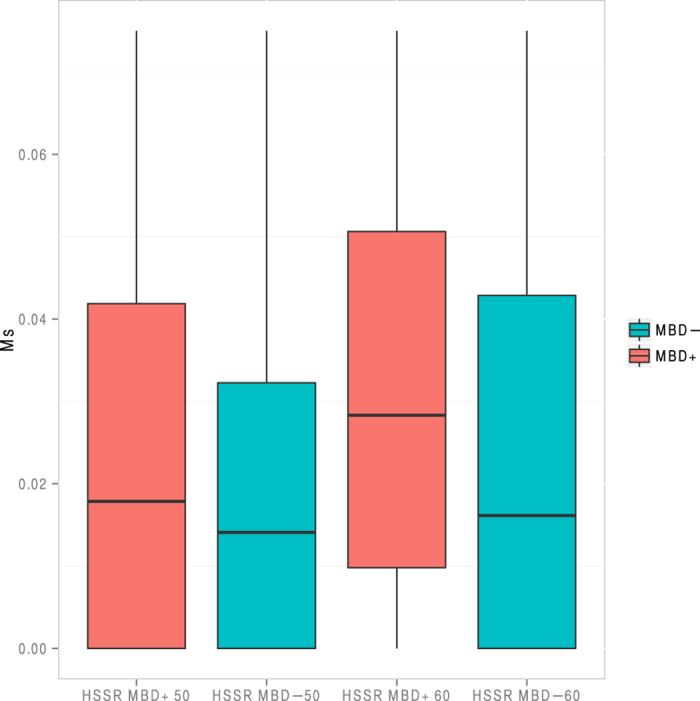
Measure of regional methylation level based on the average CpG→TpG mismatch frequencies observed at read starts (Ms), as defined by Pedersen and colleagues^26^. Ms was calculated within 100 bp genomic windows and two minimal coverage thresholds were tested (50 and 60 per window, respectively).

To further assess the efficiency of MBD enrichment, we took advantage of the work by Pedersen and colleagues^26^, which quantified genome-wide methylation levels for the Palaeo-Eskimo Saqqaq individual. We used the sequence data presented by Rasmussen and colleagues^6^ and the regional methylation proxy (Ms) defined by Pedersen and colleagues^26^ to estimate methylation levels within 100 bp sliding windows. We then performed MBD enrichment on DNA extracts from the Palaeo-Eskimo Saqqaq individual and tested whether the MBD+ fraction covered genomic regions with higher Ms values than the MBD− fraction. MBD+ sequences were found to overlap regions with higher Ms values than MBD− sequences for the two minimal coverage thresholds considered (Fig. 2, Supplementary Table S3.5).

Altogether, the under-representation of mtDNA and CGIs in the MBD+ fraction, coupled with the over-representation of TEs in the MBD+ fraction, and the presence of DNA fragments showing higher methylation rates in the Palaeo-Eskimo Saqqaq sequence data, demonstrate the overall efficiency of MBD enrichment.

### Size and base compositional bias

**Figure 3:**
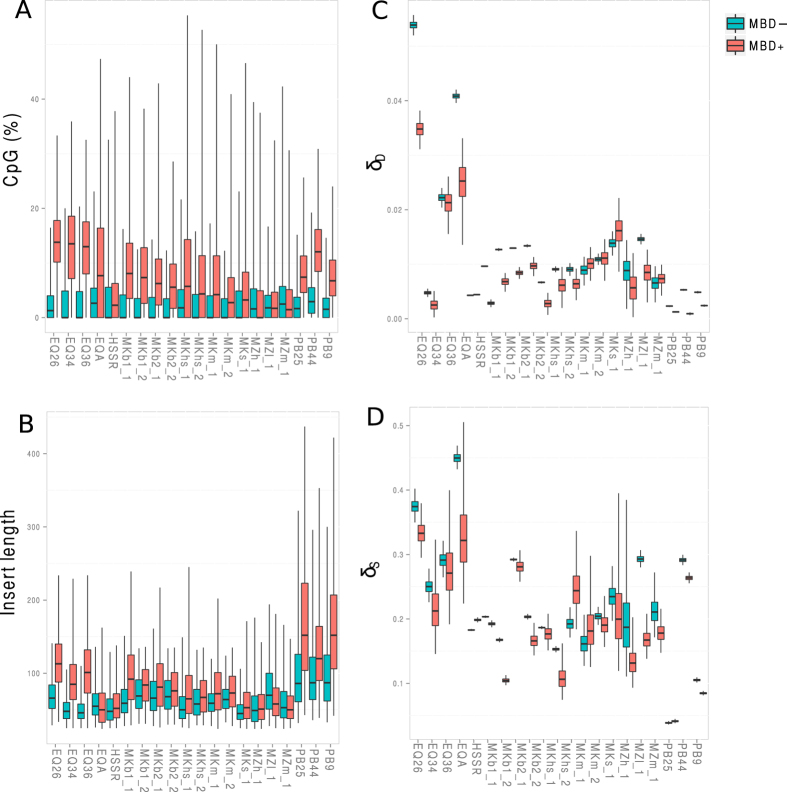
Molecular characteristics of DNA library inserts and post-mortem DNA degradation. (**A**) Percentage of CpGs in the nuclear DNA, normalized by read length. (**B**) Length of DNA library inserts. (**C**) Cytosine deamination probability in double-stranded DNA context (δ_d_). (**D**) Cytosine deamination probability in single-stranded DNA contexts (δ_s_). DNA degradation parameters were calculated using mapDamage 2^27^. A minimum threshold of 2,000 reads was applied to all data shown (mapping quality filtered, MQ25) so as to retrieve unbiased distributions. MBD+ = MBD enriched fraction. MBD− = uncaptured fraction.

We next evaluated whether endogenous DNA fragments recovered in MBD+ and MBD− fractions exhibited similar molecular properties. Only samples showing at least 2,000 high-quality hits mapping uniquely against their respective reference genome (MQ25 filter) were considered so as to enable reliable estimates of base compositional bias. We implemented linear mixed models analyses to assess statistical differences within the two fractions for %GC content, CpG densities and the size of library inserts. We found that MBD+ fractions were significantly enriched in endogenous reads carrying higher %GC contents and CpG densities, with the exception of a single sample (MZh_1, Figs 1C and 3A; Supplementary Fig. S1; Supplementary Tables S4.1 and S4.2).

In MDB+ fractions, CpG densities increased up to 3.1-fold compared to that observed for other dinucleotides (Supplementary Tables S5.1 and S5.2). Interestingly, we detected a decrease in the number of ApAs, ApTs, TpAs and TpTs in the MBD+ fraction, coupled with an increase in the number of CpCs, GpCs and GpGs. These differences were statistically significant when tested for the three species with a minimum number of three individuals (except for CpG and GpC in polar bears; Supplementary Table S5.2). We also found that endogenous DNA inserts from MBD+ fractions were, on average, longer than in MBD− fractions (Fig. 3B). This difference was statistically significant in the woolly mammoth (with the exception of MZh_1, Supplementary Table S4.2), where MBD+ DNA inserts were on average ~21-bp longer than those from the MBD− fraction. MBD+ DNA library inserts were on average ~35-bp longer than those present in the MBD− fraction amongst equids and ~56-bp longer amongst polar bears. These differences were statistically significant (Supplementary Tables S4.1 and S4.2).

We observed that the relative amount of endogenous inserts showing at least one CpG generally increased with the size of DNA inserts. This trend was only apparent in MBD+ fractions, however, and the inverse was found in MBD− fractions (Supplementary Fig. S4), suggesting that MBD enrichment separated methylated and unmethylated fractions more efficiently on long DNA templates. This most likely reflects enhanced DNA binding to MBD2−Fc with increasing numbers of ^m^CpG targets, as has been previously reported^19^.

The bias observed toward DNA templates that are longer and richer in ^m^CpG is expected to contribute to the reduced molecular complexity of MBD+ fractions as the vast majority of endogenous DNA templates present in aDNA extracts consists of short DNA molecules^3^,^4^. To test this, we calculated the number of PCR duplicates found in the MBD+ fractions (relative to the MBD− fractions, MQ25 filter; Supplementary Table S2.2), which provides a measure of the loss of molecular complexity in the MBD+ fraction. We observed an inverse relationship with the median size of MBD+ endogenous inserts (Supplementary Fig. S3). No relationship was found with other DNA damage related parameters (data not shown), confirming that the level of DNA fragmentation is a fundamental limitation in respect to the efficiency of MBD enrichment.

### DNA damage

Given that *post-mortem* cytosine deamination transforms methylated CpGs into TpGs and that the efficiency of MBD2−Fc binding increases for ^m^CpG-rich DNA templates, we next evaluated whether MBD enrichment was biased against recovering fragments altered by *post-mortem* cytosine deamination, as described by Smith and colleagues^22^. We used mapDamage 2^27^ to quantify a series of *post-mortem* DNA damage parameters for each DNA library, including cytosine deamination rates at overhangs (δ_s_) and in double-stranded (δ_d_) DNA contexts, and a proxy for the average length of overhanging ends (λ). We applied a threshold of 2,000 high-quality hits (MQ25 filter) as described above to the estimation of damage parameters.

Although not statistically significant (Supplementary Tables S4.3, S4.4 and S4.5), we found that both δ_s_ and δ_d_ rates were generally lower in the MBD+ fraction than in the MBD− fraction of a given specimen (Fig. 3C,D). On average, we found a 1.8-fold reduction in the δ_d_ rates of MBD+ fractions (relative to MBD− fractions). This reduction was of lower magnitude for δ_s_ rates (1.2-fold). This probably reflects that ^m^CpGs present in overhangs of aDNA templates have a limited contribution to DNA binding to MBD2−Fc, probably owing to their limited size (estimated here to be 0.9–2.8 bp-long on average; Supplementary Fig. S2). Accordingly, no particular shift was observed in the length of overhangs from MBD+ and MBD− fractions (Supplementary Table S4.5, Supplementary Fig. S2).

**Figure 4:**
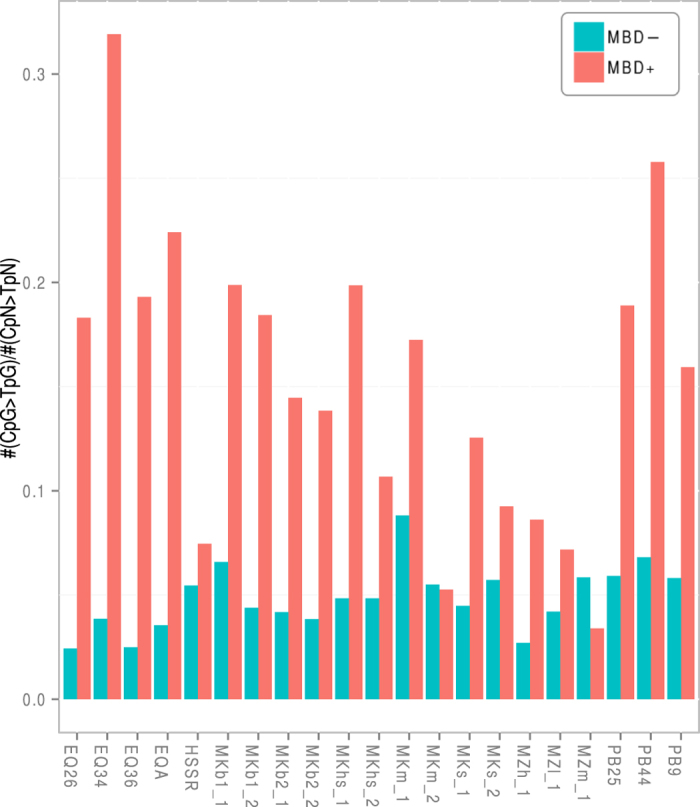
Relative amount of CpGs to TpGs transitions over all other kind of possible C->T mutations in CpN contexts. A minimum threshold of 2,000 reads was applied.

We next evaluated whether cytosine deamination occurs at faster rates when CpG dinucleotides are methylated, as expected from genetic assay based on reversion of mutations in single-stranded^28^ and double-stranded^29^ DNA contexts and as previously observed in archeological barley material from Qasr-Ibrim, Egypt^22^. For this, we used C→T mutation rates to calculate the relative proportion of cytosine deaminations detected at CpG sites and other CpN dinucleotide contexts (MQ25 filter; Fig. 4). We observed that this proportion was up to 8.3-fold higher in endogenous sequences from the MBD+ fractions relative to MBD− fractions (Supplementary Table S3.6), except for two samples showing minimal sequence data (MKm_2 and MZm_1). This adds to the body of empirical evidence supporting faster cytosine deamination rates at methylated epialleles^22^,^29^. This fast decay of ^m^CpGs also participates to reducing the molecular complexity of MBD+ libraries.

### Depth-of-coverage

**Figure 5:**
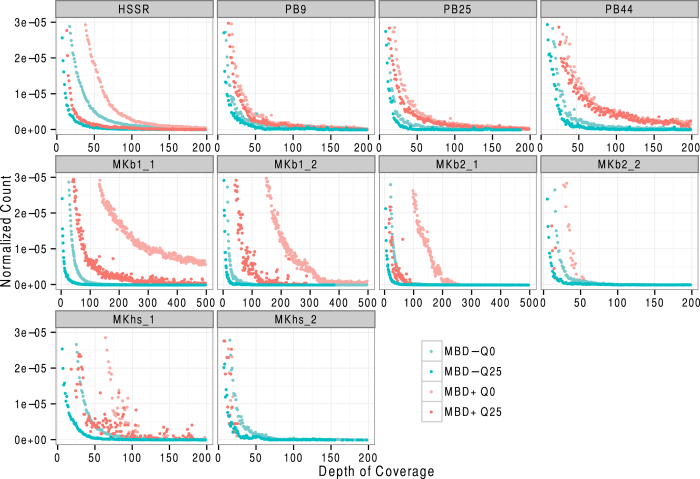
Per-base coverage distributions. The dominant class of uncovered bases is not shown and the distributions are restricted to bases covered at least once in the nuclear genome. A minimum threshold of 2,000 reads was applied.

We next tested for differential patterns of depth-of-coverage between MBD+ and MBD− fractions (Fig. 5). These analyses were restricted to samples showing sufficient sequencing data (namely polar bear, the Palaeo-Eskimo Saqqaq and the Khroma mammoth bones and hair/skin samples), and were normalized by the overall depth-of-coverage for bases covered at least once and performed on both quality-filtered (MQ25) and not quality-filtered (MQ0) reads. MBD+ distributions were shifted towards higher coverage when compared to MBD− distributions (Fig. 5). This indicates that for a given sequencing effort, MBD enrichment can achieve the sequencing of target regions to a higher coverage relative to no enrichment.

### Microbial contents

Although MBD enrichment is designed to target vertebrate methylated DNA, MBD+ libraries were found to include DNA inserts of microbial origin (Supplementary Table S6). To compare the bacterial diversity of MBD+ and MBD− fractions, we excluded samples for which less than 2 *genera* were identified in either of the two fractions. The microbial profiles obtained from MBD− fractions segregated according to the type of environment in which the samples were collected (permafrost for mammoths and polar bears *vs.* caves for equids; Supplementary Fig. S7). At the *genus*-level, MBD+ and MBD− fractions were more similar to soil microbial communities than to a broad repertoire of human microbiomes (Supplementary Fig. S6). This is in line with previous studies^5^ and rules out extensive DNA contamination from microbes of human origin during and after excavation.

For most samples, MBD+ and MBD− fractions showed similar Shannon diversity indices (Supplementary Table S7) and little segregation by hierarchical clustering (Supplementary Fig. S5). This confirms that MBD enrichment does not significantly bias microbial profiles towards a particular bacterial group, as previously shown^19^. The equine specimens, however, except for sample EQB, stand as an exception. In these samples, class-level profiles showed differences between the MBD+ and MBD− fractions (Supplementary Fig. S8), and Linear Discriminant Analyses (LDA)^30^ identified significant enrichment for *Afipia*, *Rhodopseudomonas*, unclassified *Methylocystaceae* and *Acetobacteraceae* in MBD− fractions, and for *Burkholderia* and *Pseudomonas* in MBD+ fractions (Supplementary Fig. S9). This suggests that, in these cases, MBD enrichment shows some level of selectivity with regard to the enrichment of certain microbial DNA present in the DNA extracts.

## Discussion

**Figure 6:**
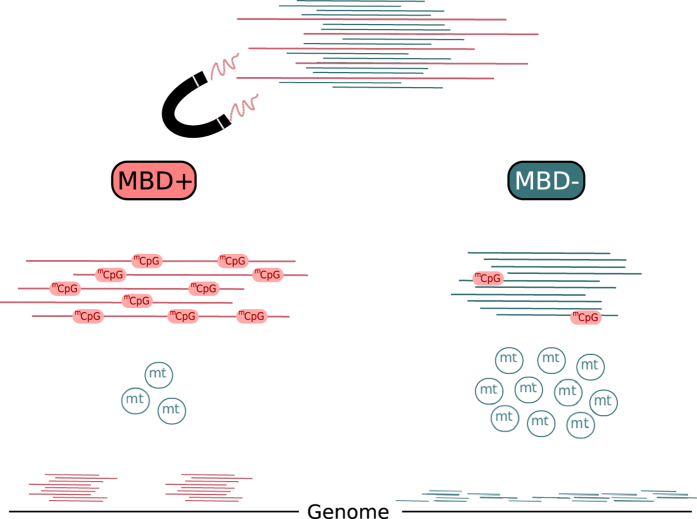
Properties of MBD enrichment for ancient DNA. The MBD+ fraction favors DNA templates with a longer size and a higher number of ^m^CpGs. This represents a limited fraction of the molecules present in aDNA extracts. mtDNA is under-represented in the MBD+ fraction while higher coverage is observed for hyper-methylated regions of the nuclear genome, including TEs.

The recent discovery that epigenetic marks can be tracked through evolutionary time has opened new avenues for aDNA research^22^,^31^,^32^,^33^. In this study, we explore the efficiency and potential biases that may result from MBD enrichment of aDNA extracts (Fig. 6). Not unexpectedly, the method appears to be principally biased towards recovering CpG-rich templates. As a result, long molecules, which show a higher density of CpGs, and therefore higher binding potential to MBD2−Fc, are preferentially enriched. The specificity of the method toward ^m^CpGs is also apparent in the Palaeo-Eskimo Saqqaq sequence data, where the MBD+ fraction shows higher regional methylation scores than the MBD− fraction, in the lower coverage of mtDNA and CGI, and in the over-representation of TEs in MBD+ fractions. In addition, DNA templates for which fewer cytosines have been deaminated (and therefore for which fewer ^m^CpGs were converted to TpGs, which represent inappropriate targets for MBD enrichment), are over-represented in enriched fractions.

These features have several important consequences for the analysis of aDNA extracts. First, aDNA molecules are extensively fragmented after an organism dies. Real-time PCR experiments have shown a 10 to 100-fold decrease in the number of amplifiable templates for every doubling of the amplicon sizes^34^. NGS data from a range of environmental conditions have confirmed that endogenous aDNA molecules become exponentially rare with increasing fragment length. Although nucleosomes can sometimes protect DNA from fragmentation and introduce periodicity patterns in depth-of-coverage and size distributions^26^, DNA inserts rarely exceed 100 bp. The observed bias towards recovering long DNA fragments, thus, unavoidably results in a decrease in the total number of molecules that are available for sequencing in the MBD+ fractions post-enrichment, which translates into reduced molecular complexity (and increased clonality) of DNA libraries. This bias is enhanced due to the faster cytosine deamination rates observed for methylated epialleles, since the relatively few number of methylated sites present in short templates will be relatively rapidly converted into inappropriate TpG targets. Additionally, from the billions of nucleotides that comprise mammalian genomes, only ~30 million CpGs are found^20^,^35^, of which only a subset are methylated in a tissue-specific manner. This means that the pool of endogenous molecules available for MBD enrichment shows lower complexity than the overall genome. We also found that microbial and organellar DNA templates represent an important proportion within MBD+ fractions and with the experimental conditions presented here, the population of endogenous sequences that lack CpGs but are still present in MBD+ fractions represents 1.0–3.2% of the data for polar bears, 2.9–30.9% for equids, 13.4–48.8% for woolly mammoths and 46.28% for the Palaeo-Eskimo Saqqaq (Supplementary Tables S3.4). Altogether, the relatively limited efficiency and drop in complexity associated with MBD enrichment as well as the substantial off-target carry-over observed suggest that MBD enrichment is probably not adequate for analyzing the vast majority of aDNA extracts, which generally show extensive DNA fragmentation, deamination, and microbial contamination.

However, despite the biases described above, MBD enrichment provided access to a fraction of the methylome from a diversity of remains originating from both permafrost and non-permafrost environments. This demonstrates that a significant proportion of ^m^CpGs are still intact in a variety of aDNA extracts. Importantly, for the three polar bear samples, the total fraction of the genome that is covered after sequencing MBD+ fractions, as calculated from the cumulative length of unique high-quality hits observed for every million sequences generated, was found to be, on average, higher in MBD+ than in MBD− fractions (0.041-fold *vs.* 0.027-fold; Supplementary Table S2.4). It follows that for these samples, the reduction in complexity and endogenous content of DNA libraries constructed from MBD+ fractions is compensated for by an increase in the DNA library insert size. Common to these samples is a high endogenous content (23.3–46.7%; Supplementary Table S2.1) and relatively moderate fragmentation levels (endogenous DNA fragments in MBD+ and MBD− fractions are on average 62.3 and 100.0 bp long, which is 48.1–99.4 bp longer than that of the Palaeo-Eskimo Saqqaq; Fig. 3B, Supplementary Table S4.1).

We therefore suggest that for similar samples, MBD enrichment holds the potential to be used to obtain complete genome sequences while measuring ancient methylation levels at the same time. This most certainly restricts the applicability of the method to the most recent and/or most preserved specimens, which represents only a minority of specimens present in the paleontological and archeological record. We, thus, caution against the method for DNA extracts showing significant levels of deamination and fragmentation (e.g. median sizes ≤80 bp).

Ideally, both MBD+ and MBD− fractions should be sequenced to help identify regions differentially covered in the two fractions. This might provide in the future an alternative to methods currently available for recovering ancient epigenetic information based on bisulphite conversion^31^ and differential patterns of CpG→TpG mutations at methylated and unmethylated sites^26^,^32^. Given the decrease in endogenous DNA content and the increase in clonality observed for MBD+ libraries, we recommend performing MBD enrichment on large volumes of aDNA extracts whenever possible. This will increase the pool of DNA templates available for enrichment and the molecular complexity of DNA libraries constructed on MBD+ fractions. Alternatively, DNA extracts that have been concentrated following additional purification could be used for MBD enrichment (although this will also lead to a substantial loss of DNA molecules during purification). In recognition of the observed bias towards CpG-rich DNA fragments, we recommend the use of specific DNA polymerases, such as Accuprime *Pfx*, and, more generally, PCR conditions that limit the over-amplification of %GC-rich DNA templates^36^. Other modifications to the procedure described here, for example involving different washing and elution conditions following DNA binding to MBD2−Fc^37^, might also contribute toward releasing the whole diversity of methylated DNA molecules present in the extract and limiting the carry-over of organellar and other unmethylated templates into the MBD+ fraction.

We note that the observed preference for ^m^CpG-rich fragments introduces a bias in the quantification of *post-mortem* DNA degradation. Specifically, DNA templates where ^m^CpG sites have been less affected by cytosine deamination – the most common aDNA damage – are more likely to be enriched. Epialleles also show higher deamination rates while methylated than not. The resultant pool of DNA fragments that make up the MBD+ fraction is, thus, unlikely to reflect the true level of *post-mortem* DNA damage. This observation was first noted by Smith and colleagues^22^, who described deamination reactions at unmethylated and methylated cytosines as two complementary forces, both contributing to the global estimate of *post-mortem* DNA deamination rates.

Although DNA degradation reactions are known to take place at faster rates with increasing temperature at depositional sites^38^, we observed striking differences in the molecular preservation of the different woolly mammoth tissues (Table 1; Fig. 3). For Khroma, bone endogenous DNA content was 1.6 to 140-fold higher than in soft tissues (Supplementary Table S2.1), and cytosine deamination occurred up to 2.4-fold slower (Fig. 3, Supplementary Table S4.4). This suggests that DNA degradation and *post-mortem* microbial colonization are greater in soft tissues, most likely due to the rapid collapse of cellular membranes and disruption of the tissue organization, are greater in soft tissues, as previously suggested^39^.

Our data also indicate that cytosine deamination levels cannot provide a direct estimate of the time since a fossil was buried, since we observed a large variation in cytosine deamination levels at overhangs across the tissues from the same mammoth specimen (Khroma; Fig. 3D, Supplementary Table S4.4). This contrasts with the linear relationship between time and C→T nucleotide misincorporation rates at sequence starts observed by Sawyer and colleagues^40^ in a diversity of preservation environments, but is in line with other studies inside^41^ and outside^17^ permafrost regions, where high variability of deamination rates where observed across DNA libraries prepared from the same bone samples^42^. This is also in agreement with previous reports showing different deamination rates in the DNA molecules isolated from pathogenic microbes and those from their human host^43^.

In this study, we have identified DNA fragmentation and degradation as the most important limiting factor for the efficiency of MBD enrichment. We have suggested experimental conditions and procedures to help characterize ancient methylation marks through MBD enrichment. Pending technologies capable of accurately identifying base modifications while sequencing, MBD enrichment provides a method complementary to those currently available for characterizing ancient methylomes but appears only appropriate for a limited fraction of ancient specimens affected by minimal DNA fragmentation and deamination. Together, these approaches are essential steps towards the evaluation of the role of epigenetic reprogramming during major evolutionary transitions^33^, such as the Industrial Revolution, the transition from hunting-gathering to farming, the domestication of plants and animals and the climatic warming that followed the Last Glacial Maximum.

## Methods

### Samples and DNA extraction

We extracted aDNA from 20 ancient and palaeontological specimens (human, mammoth, polar bear and several equine species) using previously published methods from^6^,^41^,^44^ (Table 1 and Supplementary Table S1). DNA extraction, MBD enrichment and DNA library building were performed in state-of-the-art aDNA facilities at the Centre for GeoGenetics, Copenhagen, Denmark. Specimens were selected to represent a range of time periods (Holocene and Late Pleistocene) and environmental preservation conditions (permafrost and caves).

Since methylomes are tissue-specific, we investigated the robustness of the MBD enrichment approach to tissue types using both calcified and soft tissues, taking advantage of two exceptional woolly mammoth specimens known as Khroma and Zhenya. Khroma was excavated in October 2008 in the Khroma River, Yana-Indigirka lowland, Yakutia, Eastern Siberia; the specimen’s radiocarbon age was >45,000 years^45^. We extracted 0.137 to 1.350 grams of fresh samples from skeletal muscle, a piece of skin, a piece of skin covered with hair, and two different bones. For Zhenya, which was radiocarbon dated to 44,750 (+950/−700) year BP and excavated in September 2012 on the right bank of the Yenisei River Gulf, Taymyr Peninsula, Western Siberia^46^, 0.195–1.447 grams of fresh samples of liver, skeletal muscle and cardiac muscles were extracted for DNA. All aDNA extracts were stored at −20 °C before being subjected to MBD enrichment.

### MBD enrichment

MBD enrichment of the aDNA extracts was performed using either the EpiMark^®^ Methylated DNA Enrichment Kit (catalogue number E2600, New England BioLabs) or the NEBNext^®^ Microbiome DNA Enrichment Kit (E2612), with slight modifications (Supplementary Table S1). For each enrichment reaction, we coupled 10 μL of human MBD2 protein fused to the Fc tail of human IgG1 (MBD2−Fc) to 1 μL of paramagnetic protein A beads (MBD2−Fc/Protein A Magnetic Bead) by incubating with 11 μL of 1X Bind/Wash Buffer for 15 min at Room Temperature (RT) with agitation. MBD2−Fc/Protein A Magnetic Beads were then concentrated using a magnetic rack and washed twice using 1 mL 1X Bind/Wash Buffer before being re-suspended in 11 μL 1X Bind/Wash Buffer. A volume of 10 μL of washed beads was mixed with 21.25–50 μL of aDNA extract, 20 μL of 5X Bind/Wash Buffer and supplemented with sterile water up to 100 μL, before being incubated for 20 minutes at RT with agitation. The fraction of DNA molecules that could bind to MBD2−Fc/Protein A Magnetic Beads was concentrated using a magnetic rack. The supernatant was saved as the fraction of the extract that could not bind to the MBD2−Fc/Protein A Magnetic Beads (hereafter, MBD−). MBD2−Fc/Protein A Magnetic Beads were washed three times with 1 mL of 1X Bind/Wash Buffer (or 1 mL of 0.4 M KCl for the Palaeo-Eskimo Saqqaq sample), before the fraction enriched for methylated DNA (hereafter, MBD+) was eluted in 150 μL TE with 15 μL proteinase K after 30 min incubation at 37 °C (except for the Palaeo-Eskimo Saqqaq MBD+, which was eluted in 100 μl of 1 M KCl, after 1 h incubation at 37 °C). Both MBD+ and MBD− fractions were purified using MinElute columns (QIAGEN) and 21.25 μL of EB buffer following the manufacturer’s instructions, except that the final elution was performed following a 15 min incubation step at 37 °C. For one polar bear sample (PB44), this MinElute purification step was replaced by ethanol precipitation and resuspension in 21.25 μL EB and 15 min incubation at 37 °C (Supplementary Table S1).

### DNA library preparation and sequencing

For each sample, we constructed Illumina DNA libraries using both MBD+ and MBD− fractions and the procedure from Meyer and Kicher (2010)^47^, with the slight modifications from Seguin-Orlando and colleagues^48^. Half the volume of each library was amplified in a 50 μL PCR reaction using 5 units AmpliTaq Gold (Life Technologies). PCR cycling conditions consisted of an initial denaturation step for 10 min at 92 °C, followed by 8 to 15 cycles of 30 sec denaturation at 92 °C, 30 sec annealing at 60 °C and 40 sec elongation at 72 °C. The final elongation step lasted for 7 min at 72 °C. When flat DNA profiles were observed using the 2100 Bioanalyzer (Agilent) High-Sensitivity DNA Assay, a second round of PCR amplification for 6–12 cycles was performed in four parallel reactions, as described by Seguin-Orlando and colleagues^48^. Final products were purified on a MinElute column (QIAGEN) and eluted in 20 μL EB after 15 min incubation at 37 °C. Amplified libraries were quantified using the 2100 Bioanalyzer (Agilent) High-Sensitivity DNA Assay, pooled with other indexed libraries, and sequenced on Illumina HiSeq 2500 and MiSeq platforms at the Danish National High-Throughput DNA Sequencing Centre. For all mammoth samples, the other half of each DNA library was subjected to two more independent rounds of PCR amplification and shipped to the Kurtchatov Institute, Moscow, Russia for sequencing on the GAIIx platform. For the Palaeo-Eskimo Saqqaq sample, both halves of the DNA libraries were amplified independently and sequenced in Denmark. The first position of both pair-ended reads generated in Russia was trimmed due to base calling issues at that position, after checking for DNA damage signature profiles with mapDamage 2^27^. All sequence information generated in this study is available for download at the Short Read Archive (Accession Nb. PRJNA260386).

### Sequence alignment

Sequencing reads were aligned against reference genomes using the PALEOMIX pipeline^49^. Seeding was disabled while mapping and two different alignment files were obtained per sample, one without applying a mapping quality threshold (MQ0) and one filtering for a mapping quality above 25 (MQ25 filter). For equine samples, sequencing reads were aligned against the nuclear chromosomes from the horse reference genome EquCab2^50^ and against the horse mitochondrial reference genome (Accession Nb. NC_001640.1)^51^. The sequence data from the Palaeo-Eskimo Saqqaq individual was aligned to the GRCh37/hg19 assembly of the human genome and to the rCRS sequence (Accession Nb. NC_012920) for the mtDNA^52^,^53^. The scaffolds from the African elephant genome loxAfr3 (Accession Nb. GCA_000001905.1) and the *Mammuthus primigenius* mitochondrial sequence from Rogaev and colleagues^54^ (Accession Nb. DQ316067) were used for aligning mammoth reads. For mammoth and equine specimens, we used standard mapping parameters, except that the -n option from BWA was relaxed to 0.03 to compensate for the evolutionary distance to the genomes used as reference. The polar bear nuclear scaffolds from Miller and colleagues^55^ (SRA054912) and the mitochondrial reference from Lindqvist and colleagues^56^ (Accession Nb. GU573488.1) were used to align sequencing reads generated from polar bear DNA libraries.

All the analyses described below were performed on samples that showed a minimum number of 2,000 reads for the nuDNA and 20 reads for the mtDNA.

### MBD+ *vs.* MBD− comparisons

#### DNA damage

The package mapDamage 2^27^ was used to characterize DNA fragment length distributions and nucleotide misincorporation patterns. We also estimated the parameters for the DNA damage model, namely deamination rates in double strands (*δ*_*d*_) and single strands (*δ*_*s*_), and the probability of reads not terminating in overhangs (*λ*). The analyses were run with BAM alignments, after removing duplicates and filtering for a minimum mapping quality of 25, against reference genomes as input files, with default settings. We also evaluated whether MBD+ and MBD− fractions differed in their amount of DNA damage using the two-sample t-test on the posterior means of the damage parameters *δ*_*d*_, *δ*_*s*_ and λ. All statistical tests were implemented in R version 3.0.1^57^. Tests were carried out as three independent statistical comparisons between MBD+ and MBD− fractions on samples carrying at least 2,000 reads and on species with a minimum of three samples: *Homo sapiens* (HSSR), *Mammuthus primigenius* (Khroma, Zhenya), *Ursus maritimus* (PB9, PB25, PB44), and *Equus ferus* (EQ26, EQ34, EQ36).

#### Read size, GC content and CpG densities

We performed a linear mixed effect analysis to compare MBD+ and MBD− fractions for their size distribution, %CG and CpG densities, all calculated using seqtk comp (available at https://github.com/lh3/seqtk) on collapsed reads. We used a linear mixed effect model, with the overall mean μ and the fraction type covariate τ_i_ as fixed effects:


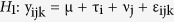



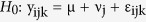


The within-sample dependence is modeled as the random factor ν_j_; furthermore the error ε_ijk_ and the ν_j_ terms are independent and identically distributed, respectively. Mixed effects model fitting was achieved by applying the lmer function (option REML = FALSE) in the R package lme4 (http://cran.r-project.org/package=achieved lme4). The asymptotic chi-square test was used to assess the statistical significance of the likelihood difference.

Linear mixed models were restricted to samples carrying a minimum of 2,000 collapsed reads in each MBD+ and MBD− fractions (one *Homo sapiens* - the Palaeo-Eskimo Saqqaq individual, twelve samples of *Mammuthus primigenius*, three samples of *Ursus maritimus*, three samples of *Equus ferus* and one sample of *Equus lambei*). The availability of different tissues for the mammoth specimens allowed us to implement one linear model per library, enabling each tissue type to be modelled separately.

#### Dimer composition

The relative representation of CpGs and all other dinucleotides in the two fractions (MBD+ and MDB−) was calculated as the total number of each possible dinucleotide normalized over the total number of dinucleotides observed. The calculation was restricted to samples carrying at least 2,000 collapsed reads. Differences between MBD+ and MBD− fractions were statistically assessed by two-sample t-tests performed on mean distributions. We carried out three independent statistical comparisons between MBD+ and MBD− fractions with respect to the three species with a minimum of three samples: *Mammuthus primigenius* (Khroma, Zhenya), *Ursus maritimus* (PB9, PB25, PB44), and *Equus ferus* (EQ26, EQ34, EQ,36).

#### Genome coverage

We investigated possible differences in patterns of genome coverage between samples, tissues and MBD+/MBD− fractions, focusing on the Palaeo-Eskimo Saqqaq individual, polar bear samples and the mammoth bones, hair and skin tissues, which all showed enough sequencing data to implement such analyses (above 0.0005X mean genome coverage). We used samtools depth^58^ to retrieve the distribution of coverage for positions covered at least once. Spearman’s rank-correlation was carried out on a random subset of two million reads per sample to statistically assess the relationships between MBD+ and MBD− fractions for differential coverage per mapping position in the nuclear genome.

#### Distribution of transposable elements

We compared MBD+ and MBD− fractions for their distribution of TEs using two complementary approaches. First, we used the proportion of reads with one or more sub-optimal reads (non-null X1:i tag and null X0:1 tag) as a proxy for TEs, since TEs represent regions found at multiple copies in the genome. Secondly, we directly estimated the relative amount of repetitive elements in MBD+ and MBD− fractions, using the human, equine and elephantid sequence data, as BED annotations for repetitive elements were available from the UCSC Genome Browser and for the polar bear we used the annotation kindly made available by the authors from Liu and colleagues^59^. Coverage within and outside annotated repetitive elements was calculated using samtools depth^58^.

#### Distribution of CpG islands

We compared the MBD+ and MBD− fractions for their representation in CpG islands (CGIs), which are globally hypo-methylated^25^. This analysis was restricted to the human and equine data, since BED annotations of those genomes were available for CGIs. Samples showing less than 2,000 collapsed reads mapping to such regions were disregarded to enable reliable coverage estimates. Coverage within and outside the CpG islands was calculated using the coverage tool from PALEOMIX^49^.

#### Read length and presence of CpG

We tested if the length shift toward longer reads in the MBD+ fraction was related to the presence of CpGs by calculating the relative abundance of reads showing the presence of at least one CpG in MBD+ and MBD− within different size categories (<30 bp, 30–40 bp, 40–50 bp, 50–60 bp, 60–70 bp, 70–80 bp, 80–90 bp, 90–100 bp, 100–200 bp, 200–300 bp, >300 bp). The number of reads with at least one CpG within a category was normalized over the total number of reads in that category and the relative importance of that specific category in the total number of reads considered.

### MBD+ and MBD− microbial profiling

We compared MBD+ and MBD− fractions for their distribution of microbial taxonomic groups and their respective relative abundances. Microbial profiles were obtained using MetaPhlAn (version 1.7.7^60^) as implemented within the metagenomic module of PALEOMIX^49^ and as described in Der Sarkissian and colleagues^5^ and Schubert and colleagues^49^. To limit the impact of false positive identification in downstream analyses, low-abundance taxa (less than 1%) were disregarded.

The Shannon diversity index of each microbial profile was computed using genus relative abundance data and the function diversity of the *vegan* package in R (http://cran.r-project.org/package=vegan). Principal Coordinate Analysis (PCoA) of Bray-Curtis distances among profiles was performed at the genus level using the R function pcoa. Manhattan distance-based relationships amongst profiles were assessed at the genus level by hierarchical clustering using the R package pvclust^61^. The support for each cluster was estimated through p-values (Approximately Unbiased and Bootstrap Probabilities) estimated from 10,000 bootstrap pseudo-replicates. Further statistical comparisons between the microbial profiles of MBD+ and MBD− fractions were carried out using the program LEfSe^30^. Consistent differences in taxon abundances were tested using the non-parametric Kruskal-Wallis sum-rank test and the unpaired Wilcoxon test. A linear discriminant analysis also estimated the effect size of taxonomical covariates at the origin of the fraction differences (threshold on logarithmic linear discriminant analysis score for discriminative features = 2.0). Microbial profiles were also compared with publicly available MetaPhlAn profiles of soil^5^,^62^ and human samples^63^ by PCoA based on Bray-Curtis distances.

## Additional Information

**Accession codes**: All sequences generated in this study are available for download at the Short Read Archive (Accession Nb. PRJNA260386).

**How to cite this article**: Seguin-Orlando, A. *et al.* Pros and cons of methylation-based enrichment methods for ancient DNA. *Sci. Rep.* **5**, 11826; doi: 10.1038/srep11826 (2015).

## Supplementary Material

Supplementary Information

Supplementary Table S1

Supplementary Tables S2-S3

Supplementary Tables S4-S5

Supplementary Tables S6-S7-S8

## Figures and Tables

**Table 1:** Sample information.

Genus species	Location	Age (years Before Present)	ID	Tissue	Volume of extract captured(μl)	Total extract volume (μl)	PCR cycles	Lab
*Ursus maritimus*	Zhokhov Island, Russia	~8,000	PB9	bone	25	200	MBD−: 12,MBD+: 12 + 10	1
*Ursus maritimus*	Zhokhov Island, Russia	~8,000	PB25	bone	25	200	MBD−: 12,MBD+: 12 + 10	1
*Ursus maritimus*	Zhokhov Island, Russia	~8,000	PB44	bone	50	200	MBD−: 12,MBD+: 12 + 10	1
*Equus ferus*	Ice cave museum, Khatanga, Taymyr, Russia	22,887 ± 125	EQ26	bone	21.25	150	MBD−: 9 + 8,MBD+: 12 + 11	1
*Equus ferus*	Ice cave museum, Khatanga, Taymyr, Russia	27,968 ± 213	EQ34	bone	21.25	150	MBD−: 9 + 9,MBD+: 12 + 12	1
*Equus ferus*	Ice cave museum, Khatanga, Taymyr, Russia	21,477 ± 94	EQ36	bone	21.25	150	MBD−: 9 + 9,MBD+: 12 + 12	1
*Equus lambei*	Yukon Territory, Canada	Late Wisconsinian (11,000−25,000)	EQA	bone	21.25	150	MBD−: 10 + 8,MBD+: 13 + 10	1
*Hippidion sp.*	Quebrada Opache, Chile	Late Pleistocene	EQB	bone	21.25	150	MBD−: 10 + 8,MBD+: 13 + 10	1
*Hippidion saldiasi*	Betecsa-1, Chile	21,070 ± 100, 21,380 ± 100	EQC	bone	21.25	150	MBD−: 10 + 8,MBD+: 13 + 10	1
*Equus (Amerhippus) sp.*	GNL Quintero 1, Chile	24,890 ± 70 (bioapatite)	EQE	bone	21.25	150	MBD−: 10 + 8,MBD+: 13 + 10	1
*Hippidion sp.*	Baño Nuevo-I, Chile	13,275±30	EQF	bone	21.25	150	MBD−: 10 + 8,MBD+: 13 + 10	1
*Mammuthus primigenius*	Yenisei River Gulf, Taymyr Peninsula, Western Siberia	44,750 (+950/−700)	MZh_1	heart	21.25	150	MBD−: 12 + 8,MBD+: 14 + 11	1
			MZh_2	heart	21.25	150	MBD−: 8 + 6,MBD+: 12 + 9	2
			MZl_1	liver	21.25	150	MBD−: 12 + 8,MBD+: 14 + 11	1
			MZl_2	liver	21.25	150	MBD−: 8 + 6,MBD+: 12 + 9	2
			MZm_1	skeletal muscle	21.25	150	MBD−: 12 + 8,MBD+: 14 + 11	1
			MZm_2	skeletal muscle	21.25	150	MBD−: 8 + 6,MBD+: 12 + 9	2
*Mammuthus primigenius*	Khroma River, Yana-Indigirka lowland, Yakutia, Eastern Siberia	≥45,000	MKm_1	skeletal muscle	21.25	150	MBD−: 12 + 8,MBD+: 14 + 11	1
			MKm_2	skeletal muscle	21.25	150	MBD−: 8 + 6,MBD+: 12 + 9	2
			MKs_1	skin	21.25	150	MBD−: 12 + 8,MBD+: 14 + 11	1
			MKs_2	skin	21.25	150	MBD−: 8 + 6,MBD+: 12 + 9	2
			MKhs_1	hair and skin	21.25	150	MBD−: 12 + 8,MBD+: 14 + 11	1
			MKhs_2	hair and skin	21.25	150	MBD−: 8 + 6,MBD+: 12 + 9	2
			MKb1_1	bone 1	21.25	150	MBD−: 12 + 8,MBD+: 14 + 11	1
			MKb1_2	bone 1	21.25	150	MBD−: 8 + 6,MBD+: 12 + 9	2
			MKb2_1	bone 2	21.25	150	MBD−: 12 + 8,MBD+: 14 + 11	1
			MKb2_2	bone 2	21.25	150	MBD−: 8+6,MBD+: 12 + 9	2
*Homo sapiens*	Qeqertasussuk, Greenland	4,044 ± 31	HSSR	hair	70	80	MBD−: 8/10,MBD+: 12 + 10/15 + 7	1

Lab 1 = Sequencing Laboratory 1, Danish National High-Throughput DNA Sequencing Centre; Lab 2 = Sequencing Laboratory 2, Kurtchatov Institute, Moscow, Russia. The age of samples is provided in uncalibrated radiocarbon years, when available.
