# Supplementary material for: Pros and cons of methylation-based enrichment methods for ancient DNA
Source: Sci Rep. 2015 Jul 2;5:11826. doi: 10.1038/srep11826 (PMC4488743; doi:10.1038/srep11826)
Supplement: Supplementary Information [file srep11826-s1.pdf]

Pros and cons of methylation-based enrichment methods for ancient DNA

Andaine Seguin-Orlando[1,2,$], Cristina Gamba[1,$], Clio Der Sarkissian[1], Luca Ermini[1], Guillaume Louvel[1], Eugenia Boulygina[3], Alexey Sokolov[4], Artem Nedoluzhko[3], Eline Lorenzen[1,5], Patricio Lopez[6], H. Gregory McDonald[7], Eric Scott[8], Alexei Tikhonov[9,10], Thomas W. Stafford, Jr[1], Ahmed H. Alfarhan[11], Saleh A. Alquraishi[11], Khaled A.S. Al-Rasheid[11], Beth Shapiro[12], Eske Willerslev[1], Egor Prokhortchouk[3], Ludovic Orlando[1,13*].

[$] The authors contributed equally.
* Correspondence should be sent to: Dr. Ludovic Orlando, Centre for GeoGenetics, Natural History Museum of Denmark, University of Copenhagen, Øster Voldgade 5-7, 1350K Copenhagen, Denmark. lorlando@snm.ku.dk. Phone: +45 21 84 96 46; Fax: +45 35 32 23 25.

[1] Centre for GeoGenetics, Natural History Museum of Denmark, Øster Voldgade 5-7, 1350K Copenhagen, Denmark
[2] National High-throughput DNA Sequencing Centre, Øster Farimagsgade 2D, 1353K Copenhagen, Denmark
[3] National Research Centre Kurchatov Institute, 1, Akademika Kurchatova, Moscow, 123182, Russian Federation
[4] Centre Bioengineering, Russian Academy of Sciences, Prospekt 60-Letiya Oktyabrya 7/1, Moscow, 117312, Russian Federation
[5] Department of Integrative Biology, University of California, Berkeley, CA 94720, USA
[6] Department of Anthropology, Universidad de Chile, Ignacio Carrera Pinto 1045, Ñuñoa, Santiago, Chile
[7] Park Museum Management Program, National Park Service, 1201 Oakridge Drive, Suite 150, Fort Collins, Colorado 80525, USA
[8] San Bernardino County Museum, Division of Geological Sciences, 2024 Orange Tree Lane, Redlands, California 92374, USA
[9] Zoological Institute of Russian Academy of Sciences, 199034 St. Petersburg, Russian Federation
[10] Institute of Applied Ecology of the North, North-Eastern Federal University, 677980 Yakutsk, Russian Federation
[11] Zoology Department, College of Science, King Saud University, Riyadh 11451, Saudi Arabia
[12] Department of Ecology and Evolutionary Biology, University of California Santa Cruz, Santa Cruz, CA 95060, USA
[13] Université de Toulouse, UPS, UMR 5288 du CNRS, 37 allées Jules Guesde, 31000 Toulouse, France

SUPPLEMENTARY FIGURES

Supplementary Figure S1. Percentage of GCs in the nuclear DNA for the captured (MBD+) and supernatant (MBD-) fractions.

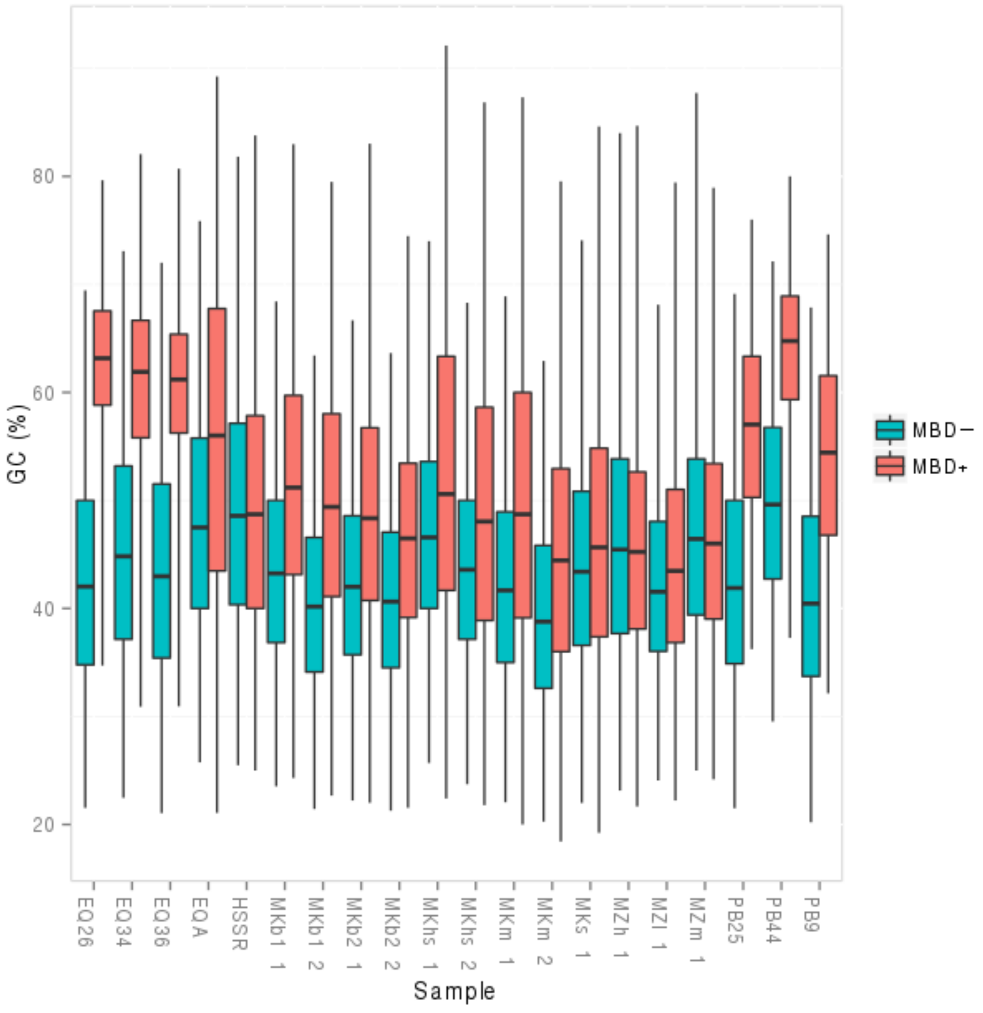

Supplementary Figure S2. Overhang length of MBD+ and MBD- fractions on the nuclear DNA obtained with mapDamage 2.0.

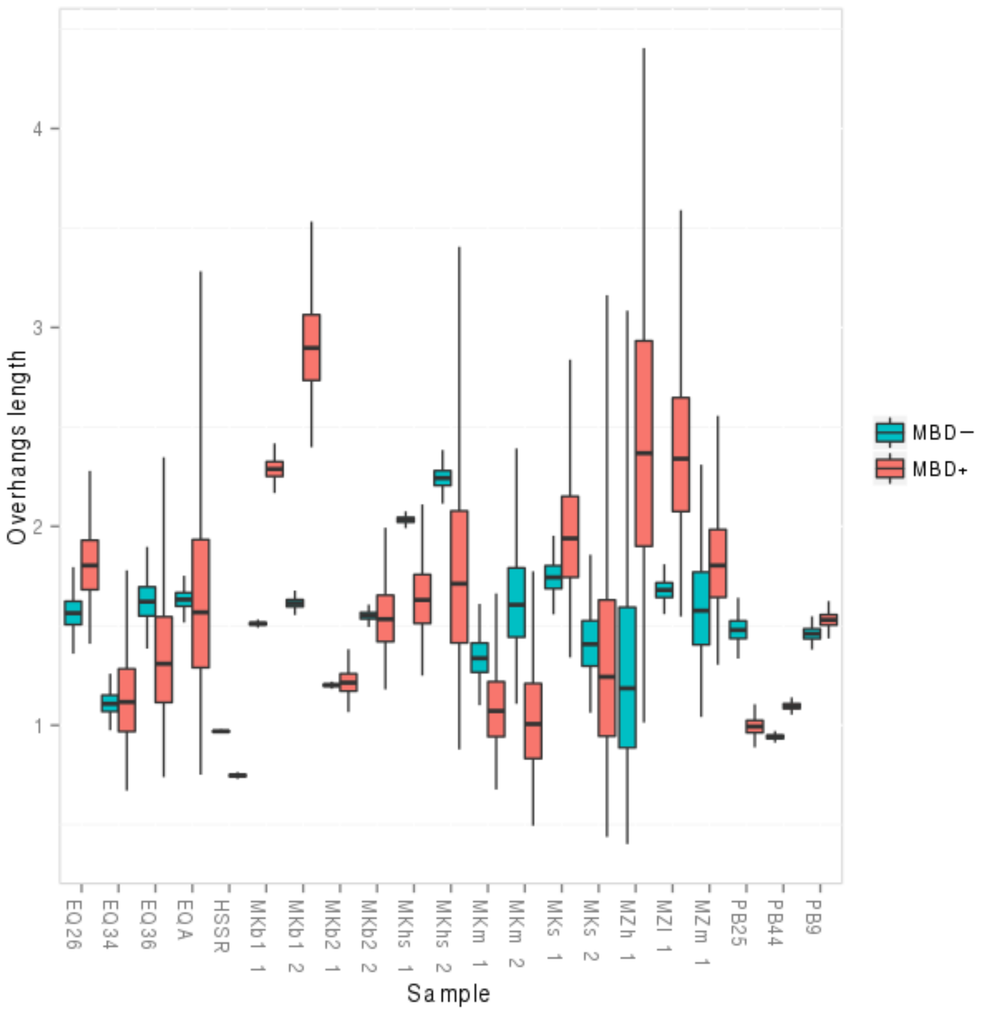

Supplementary Figure S3. Proportion of PCR duplicates in the MBD+ over the MBD- fraction vs. the median length of the fragments.

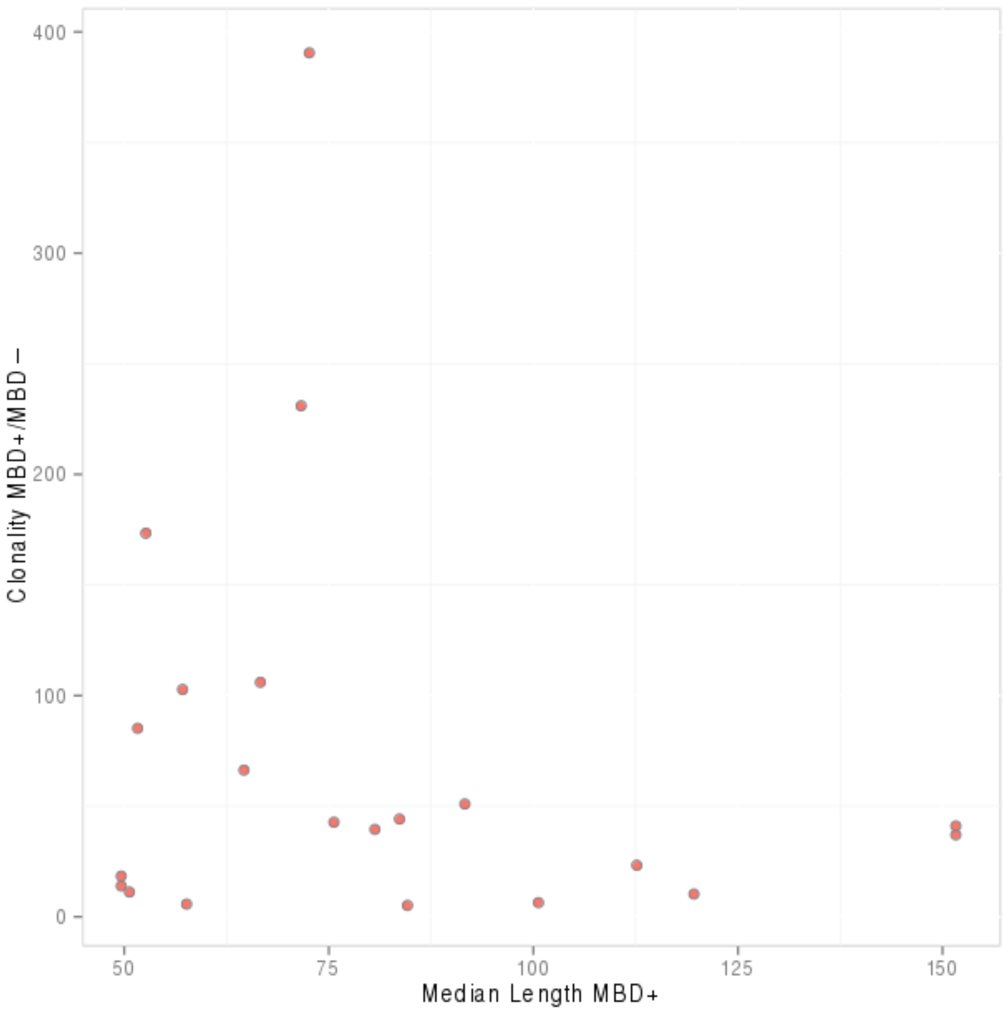

Supplementary Figure S4 - Number of reads with at least 1 CpG in the MBD+ and MBD- fractions, normalized over the total number of reads per length class (see legend) and the abundance of each class.

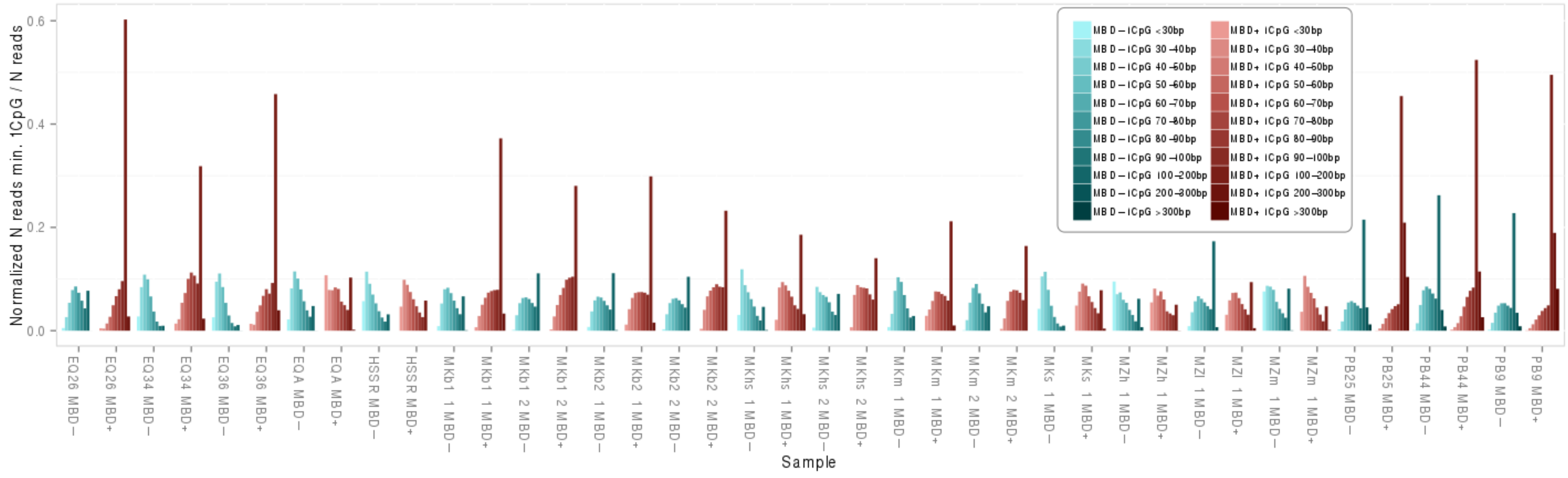

Supplementary Figure S5. Principal Coordinate Analysis of Bray Curtis distances between microbial profiles at the genus level in various soil and human-associated microbiomes, and in the captured (MBD+) and uncaptured (MBD-) fractions.

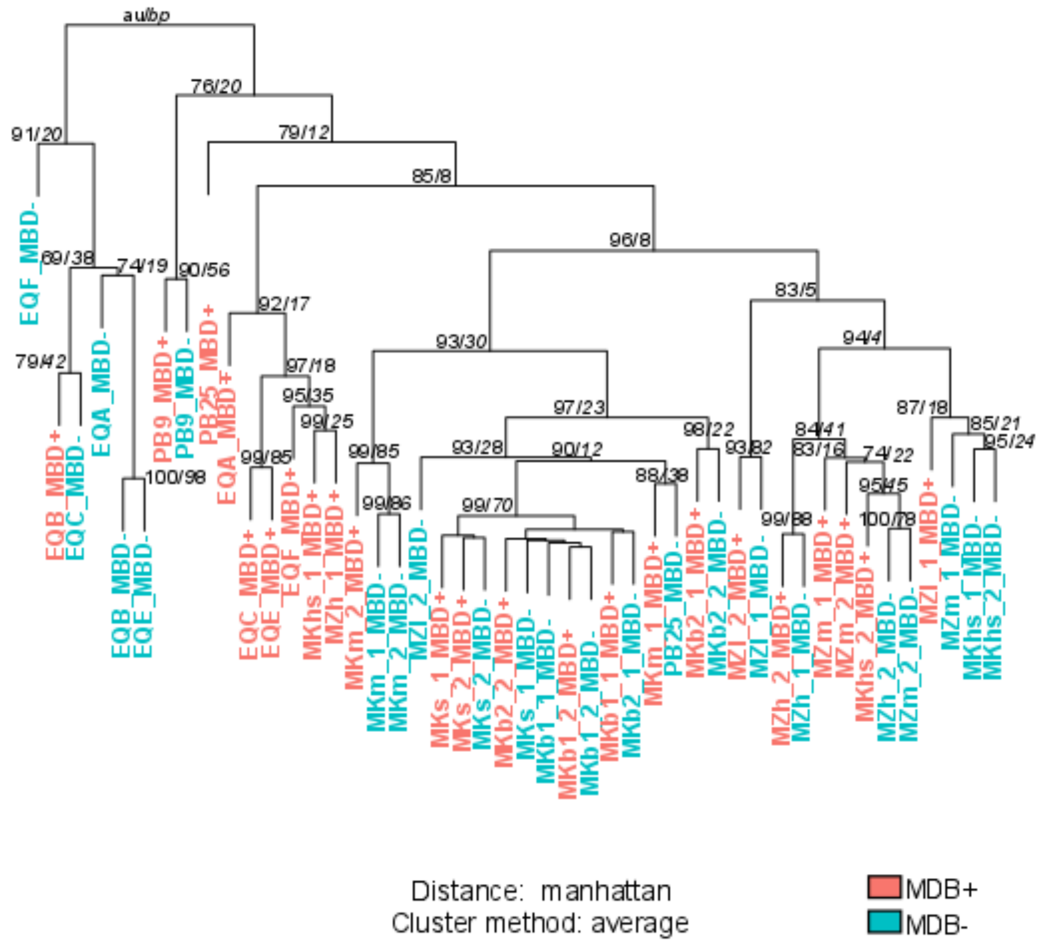

Supplementary Figure S6. Principal Coordinate Analysis of Bray-Curtis distances and hierarchical clustering of Manhattan distances between genus-level microbial DNA profiles in ancient equid, mammoth and polar bear extracts.

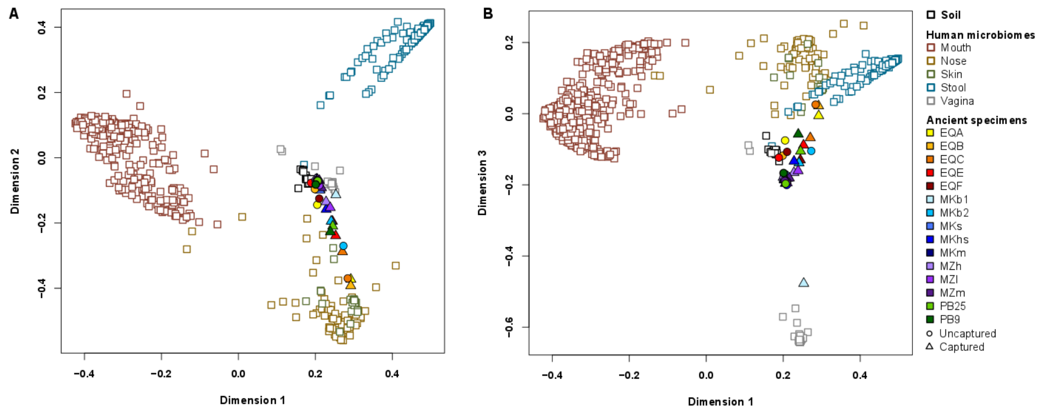

Supplementary Figure S7. Relative abundance of microbial classes in ancient equid, mammoth and polar bear DNA extracts.

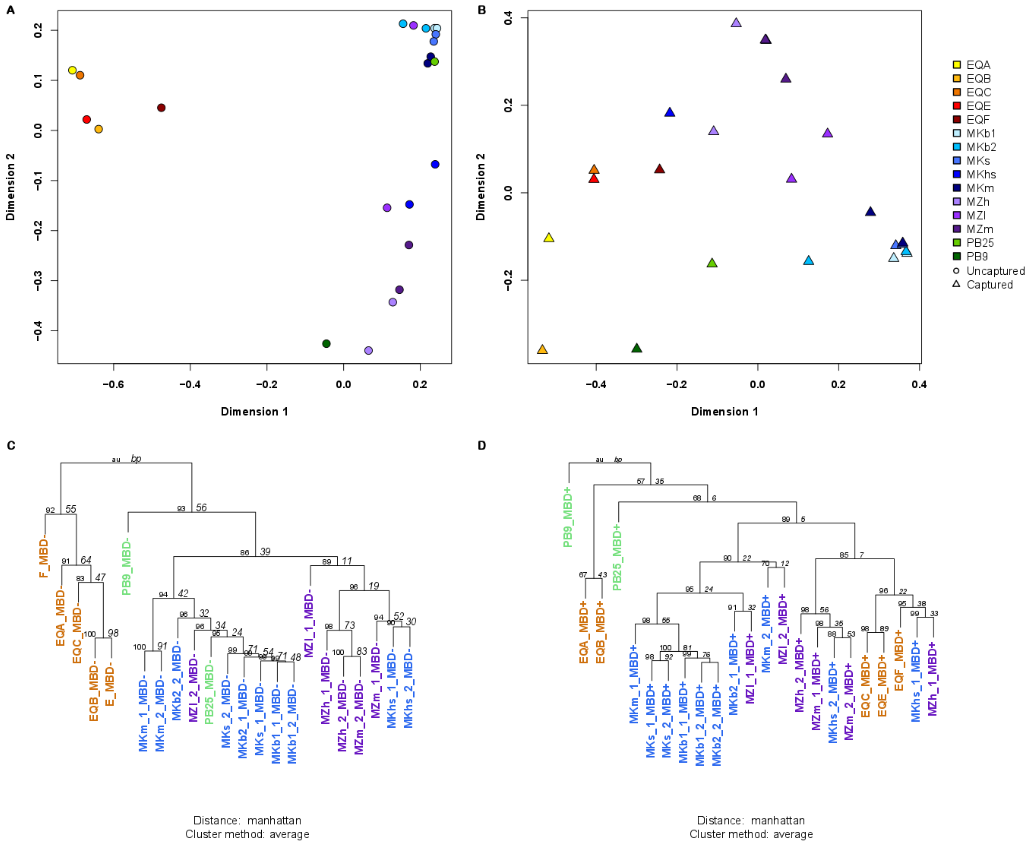

Supplementary Figure S8. Differentiating microbial features (biomarkers) among clusters of ancient equid DNA extracts, as identified and quantified by LEfSe.

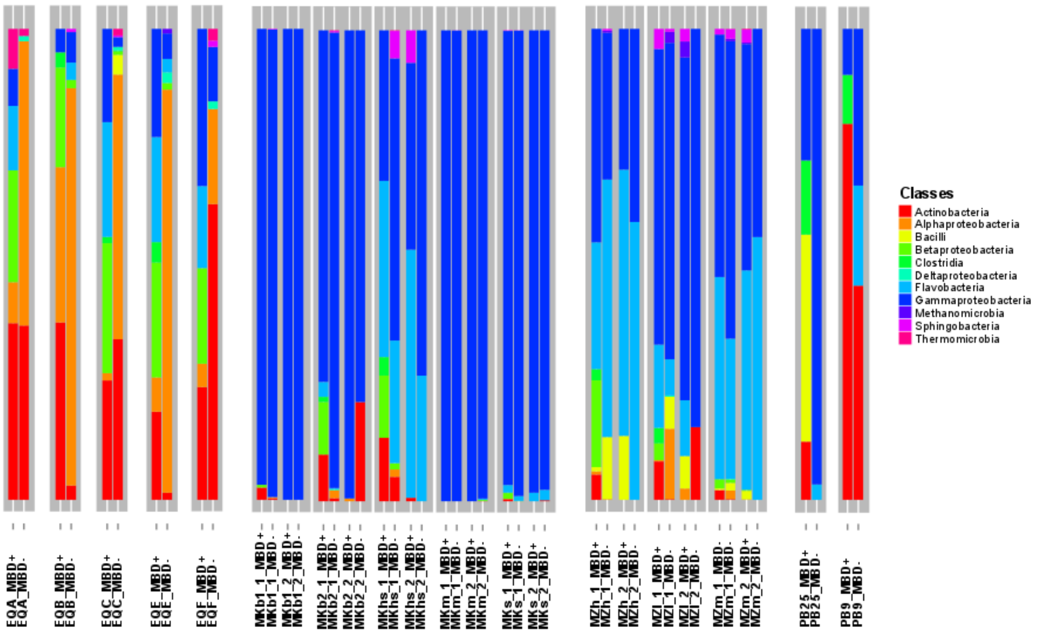

Supplementary Figure S9. Hierarchical clustering of Manhattan distances between microbial DNA profiles at the genus level in ancient equine, mammoth and polar bear extracts (10,000 bootstraps). "au", approximately unbiased p-value; "bp", bootstrap probability.

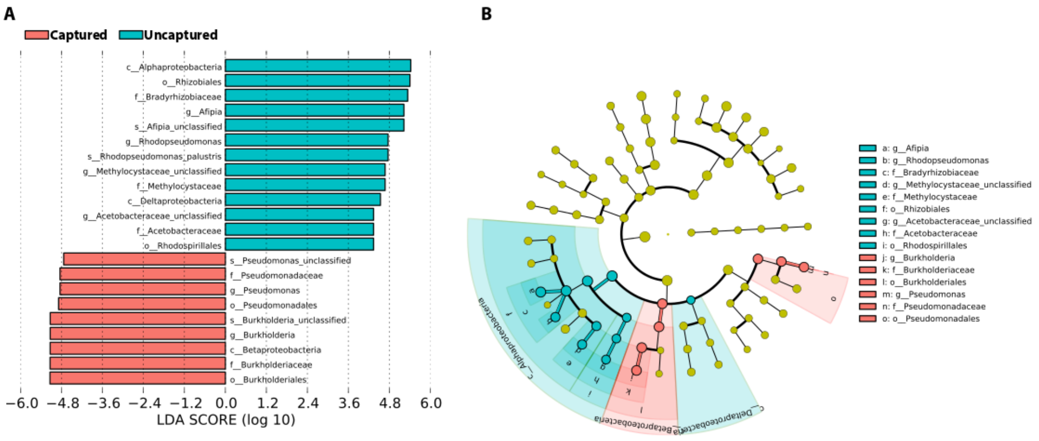

SUPPLEMENTARY TABLES LEGENDS

Supplementary Table S1. Details on the laboratory procedures pre- and post-enrichment.

Supplementary Table S2. (1) Endogenous content expressed as percentage per sample / fraction (MBD+ and MBD-) and relative amount in the 2 fractions. Both values for mapping quality filtered (Q25) and not-filtered (Q0) reads are reported. Differential representation in Khroma's bone over other tissues. (2) Percentage of clonality (number of reads originating from PCR duplicates) per sample / fraction and relative amount in the 2 fractions. Both values for mapping quality filtered (Q25) and not-filtered (Q0) reads are reported. (3) Number of hits in the nuclear DNA and mitochondrial DNA per sample / tissue and relative amount of mitochondrial DNA reads in the MBD- fraction. Both values for mapping quality filtered (Q25) and not-filtered (Q0) reads are reported. (4) Nuclear genome coverage (based on Q25 reads) per sample / fraction and average per group (EQ=Equids, M=Mammoth and PB=Polar bear). (5) Number of mitochondrial DNA copies per cell in samples with a minimum of 20 reads per fraction (based on Q25 reads). Differential representation in Zhenya's liver over other tissues.

Supplementary Table S3. (1) Coverage of CpG islands compared to the CpG frequency of MBD+ and MBD- fractions for equids, calculated on only collapsed reads above mapping quality 25. (2) Number of non-unique reads in the two fractions, calculated on only collapsed reads without filtering for mapping quality (Q0). (3) Coverage on repeated and non-repeated elements for equids and woolly mammoths and comparison between the two fractions (collapsed reads Q0). (4) Number of reads without CpGs per sample, fraction and groups (collapsed reads Q0). (5) Minimum, 1st quartile, median, mean, 4th quartile, maximum and average methylation scores (Ms), as defined in [89] for both thresholds tested on HSSR (i.e. a minimum of 50 and 60 reads where read starts overlap the reference genome at CpG sites). (6) Details on the calculation of the relative amount of CpG -> TpG over CpG to CpN. All data from this table have been calculated on samples that showed a minimum of 2,000 reads, as detailed in (7).

Supplementary Table S4. (1) Average length (avgLen), difference between MBD+ and MDB- insert size, minimum (min), median (mid), maximum (max), quantile 25 (low) and 75 (top) of insert size (Len), GC content normalized over the reads length (GC) and CpG

fraction normalized over the reads length (CpG) per sample and fraction for the nuclear DNA; calculated on collapsed reads above mapping quality 25 (Q25). (2) Statistical differences in read length, percentage of GC content and CpG density between MBD+ and MBD- fractions. The p-values were computed using linear mixed models. (3) Minimum (min), median (mid), maximum (max), quantile 25 (low) and 75 (top) of cytosine deamination probability in single strand context (δ_s, DeltaS), double strand context (δ_d, DeltaD) and overhang length calculated from the value of λ ($1/\lambda$ - 1, Overhang); calculated on all reads above mapping quality 25 (Q25). (4) Khroma's δ_d and δ_s compared between bone and other samples. All minimum, median, maximum, quantile 25 and 75 have been calculated excluding 1% outliers. (5) Statistical differences in δ_s, δ_d, and λ (used to calculate the overhang length) between MBD+ and MBD- fractions with respect to three species analyzed: *Mammuthus primigenius* (Khroma, Zhenya), *Ursus maritimus* (PB9, PB25, PB44), and *Equus caballus* (EQ26, EQ34, EQ36).

Supplementary Table S5. (1) Relative representation of dimers for each sample / fraction, calculated on samples that showed a minimum of 2,000 collapsed reads (Q25). (2) Differential representation of dimers between the MBD+ and MBD- fractions by Species.

Supplementary Table S6. MetaPhlAn microbial DNA profiles.

Supplementary Table S7. Characteristics of the microbial DNA profiles.

Supplementary Table S8. Genome reference sequences of *Burkholderia* sp. used for alignment reads from ancient equid DNA extracts.

TableS1 methods details

ID	Label	Material	Extraction Method	Capture Kit	Volume for capture (µl)	Purification prior to library building	Fraction	PCR cycles	sequencing
PB9	UM9_CGG_1_011578	bone <0.09 g	column	E2600	25 out of 200	minelute	MBD+	12	MiSeq 250 PE
							MBD-	12+10	MiSeq 250 PE
PB25	UM25_CGG_1_011594	bone <0.09 g	column	E2600	25 out of 200	minelute	MBD+	12	MiSeq 250 PE
							MBD-	12+10	MiSeq 250 PE
PB44	UM44_CGG_1_011613	bone <0.09 g	column	E2612	50 out of 200	EtOH precipitation	MBD+	12	MiSeq 250 PE
							MBD-	12+10	MiSeq 250 PE
EQ26	CGG_1_010026	bone, 2.31g	column	E2612	21.25 out of 150	minelute	MBD+	9+8	MiSeq 150 PE
							MBD-	12+11	MiSeq 150 PE
EQ34	CGG_1_010034	bone, 2.04g	column	E2612	21.25 out of 150	minelute	MBD+	9+9	MiSeq 150 PE
							MBD-	12+12	MiSeq 150 PE
EQ36	CGG_1_010036	bone, 1.74g	column	E2612	21.25 out of 150	minelute	MBD+	9+9	MiSeq 150 PE
							MBD-	12+12	MiSeq 150 PE
EQA	CGG_1_014640	bone, 0.174g	silica pellets	E2600	21.25 out of 150	minelute	MBD+	10+8	HiSeq 150 RPE
							MBD-	13+10	HiSeq 150 RPE
EQB	CGG_1_015509	tooth, 0.341g	silica pellets	E2600	21.25 out of 150	minelute	MBD+	10+8	HiSeq 150 RPE
							MBD-	13+10	HiSeq 150 RPE
EQC	CGG_1_015508	tooth, 0.372g	silica pellets	E2600	21.25 out of 150	minelute	MBD+	10+8	HiSeq 150 RPE
							MBD-	13+10	HiSeq 150 RPE
EQE	CGG_1_015510	tooth, 0.341g	silica pellets	E2600	21.25 out of 150	minelute	MBD+	10+8	HiSeq 150 RPE
							MBD-	13+10	HiSeq 150 RPE
EQF	CGG_1_014638	tooth, 0.144g	silica pellets	E2600	21.25 out of 150	minelute	MBD+	10+8	HiSeq 150 RPE
							MBD-	13+10	HiSeq 150 RPE
MZh	m1_heart_Zhenya	heart	silica pellets	E2600	21.25 out of 150	minelute	MBD+	12+8	HiSeq 150 RPE
							MBD-	14+11	HiSeq 150 RPE
							MBD+	8+6	Gallx
							MBD-	12+9	Gallx
MZl	m2_liver_Zhenya	liver	silica pellets	E2600	21.25 out of 150	minelute	MBD+	12+8	HiSeq 150 RPE
							MBD-	14+11	HiSeq 150 RPE
							MBD+	8+6	
							MBD-	12+9	
MZm	m3_muscle_Zhenya	muscle	silica pellets	E2600	21.25 out of 150	minelute	MBD+	12+8	HiSeq 150 RPE
							MBD-	14+11	HiSeq 150 RPE
							MBD+	8+6	Gallx
							MBD-	12+9	Gallx
MKm	m4_muscle_Kroma	muscle	silica pellets	E2600	21.25 out of 150	minelute	MBD+	12+8	HiSeq 150 RPE
							MBD-	14+11	HiSeq 150 RPE
							MBD+	8+6	Gallx
							MBD-	12+9	Gallx
MKs	m5_skin_Kroma	skin	silica pellets	E2600	21.25 out of 150	minelute	MBD+	12+8	HiSeq 150 RPE
							MBD-	14+11	HiSeq 150 RPE
							MBD+	8+6	Gallx
							MBD-	12+9	Gallx
MKhs	m6_hair_skin_Kroma	hair and skin	silica pellets	E2600	21.25 out of 150	minelute	MBD+	12+8	HiSeq 150 RPE
							MBD-	14+11	HiSeq 150 RPE
							MBD+	8+6	Gallx
							MBD-	12+9	Gallx
MKb2	m7_bone2_Kroma	bone	silica pellets	E2600	21.25 out of 150	minelute	MBD+	12+8	HiSeq 150 RPE
							MBD-	14+11	HiSeq 150 RPE
							MBD+	8+6	Gallx
							MBD-	12+9	Gallx
MKb1	m8_bone_pectoral_vertebra_Kroma	bone	silica pellets	E2600	21.25 out of 150	minelute	MBD+	12+8	HiSeq 150 RPE
							MBD-	14+11	HiSeq 150 RPE
							MBD+	8+6	Gallx
							MBD-	12+9	Gallx
HSSR	Saqqaq	hair, 230 mg	Phenol/chloroform + column	E2600	70 out of 80	minelute	MBD+	15+7	HiSeq 75RPE
							MBD-	10	HiSeq 75RPE
							MBD+	12+10	HiSeq 75RPE
							MBD-	8	HiSeq 75RPE

TableS2.1_endogenous content

Sample	Fraction	Endogenous content Q0 (%)	Endogenous content MBD- Q0/ Endogenous content MBD+ Q0	Endogenous content Q25 (%)	Endogenous content MBD- Q25/ Endogenous content MBD+ Q25
EQ26	MBD+	4.26	8.77	0.83	37.26
EQ26	MBD-	37.35		30.77	
EQ34	MBD+	1.60	4.06	0.28	17.14
EQ34	MBD-	6.49		4.72	
EQ36	MBD+	1.46	3.31	0.26	14.36
EQ36	MBD-	4.84		3.70	
EQA	MBD+	0.12	22.30	0.02	89.82
EQA	MBD-	2.72		2.22	
HSS	MBD+	1.91	38.19	0.94	57.01
HSS	MBD-	73.13		53.73	
HSSR	MBD+	18.49	4.91	7.08	8.51
HSSR	MBD-	90.84		60.25	
MKb1_1	MBD+	24.55	2.66	3.77	12.57
MKb1_1	MBD-	65.31		47.33	
MKb1_2	MBD+	19.89	3.30	5.03	10.08
MKb1_2	MBD-	65.72		50.73	
MKb2_1	MBD+	2.62	21.17	0.54	77.47
MKb2_1	MBD-	55.40		41.57	
MKb2_2	MBD+	2.47	22.98	1.27	34.42
MKb2_2	MBD-	56.76		43.69	
MKhs_1	MBD+	0.89	36.50	0.21	106.97
MKhs_1	MBD-	32.48		21.95	
MKhs_2	MBD+	0.32	81.82	0.14	137.56
MKhs_2	MBD-	26.00		18.83	
MKm_1	MBD+	0.17	2.38	0.07	4.38
MKm_1	MBD-	0.41		0.31	
MKm_2	MBD+	0.10	4.52	0.05	7.46
MKm_2	MBD-	0.46		0.35	
MKs_1	MBD+	0.20	3.39	0.08	5.87
MKs_1	MBD-	0.68		0.46	
MKs_2	MBD+	0.15	6.24	0.08	8.29
MKs_2	MBD-	0.91		0.66	
MZh_1	MBD+	0.10	0.66	0.05	0.84
MZh_1	MBD-	0.07		0.04	
MZl_1	MBD+	0.27	25.43	0.13	32.52
MZl_1	MBD-	6.95		4.22	
MZm_1	MBD+	0.23	0.58	0.13	0.65
MZm_1	MBD-	0.14		0.09	
PB25	MBD+	40.74	1.28	35.30	1.32
PB25	MBD-	52.09		46.69	
PB44	MBD+	54.29	0.72	23.32	1.28
PB44	MBD-	38.85		29.97	
PB9	MBD+	41.40	1.16	33.68	1.21
PB9	MBD-	48.05		40.69	
		minimum	0.58	minimum	0.65
		maximum	81.82	maximum	137.56
		average	13.47	average	30.32
		median	4.29	median	11.32

KHROMA	Fraction	Average Endogenous Content Q25	Endogenous contnent Bone / Other tissue
Muscle	MBD+	0.12	22.74
Skin	MBD+	0.08	33.59
Hair skin	MBD+	0.17	15.50
Bone	MBD+	2.65	
Muscle	MBD-	0.33	140.00
Skin	MBD-	0.46	98.84
Hair skin	MBD-	29.24	1.57
Bone	MBD-	45.83	
		**min**	1.57
		**max**	140.00
		**average**	52.04
		**median**	28.17

TableS2.2_clonality

Sample	*Fraction*	*Clonality Q0 (%)*	Clonality MBD+ Q0/ Clonality MBD- Q0	*Further Clonality Q25 (%)*	Clonality MBD+ Q25/ Clonality MBD- Q25
EQ26	MBD+	18.85	24.17	84.25	4.62
EQ26	MBD-	0.78		18.24	
EQ34	MBD+	1.92	6.00	83.09	3.03
EQ34	MBD-	0.32		27.43	
EQ36	MBD+	3.09	7.23	82.92	3.47
EQ36	MBD-	0.43		23.93	
EQA	MBD+	57.86	14.79	91.47	4.22
EQA	MBD-	3.91		21.65	
HSSR	MBD+	72.72	86.04	89.55	2.62
HSSR	MBD-	0.85		34.23	
MKb1_1	MBD+	30.61	51.88	89.35	3.20
MKb1_1	MBD-	0.59		27.96	
MKb1_2	MBD+	9.44	45.06	77.08	3.35
MKb1_2	MBD-	0.21		22.98	
MKb2_1	MBD+	67.93	40.37	93.43	3.56
MKb2_1	MBD-	1.68		26.22	
MKb2_2	MBD+	21.26	43.63	59.54	2.54
MKb2_2	MBD-	0.49		23.41	
MKhs_1	MBD+	59.71	67.12	90.71	2.75
MKhs_1	MBD-	0.89		33.03	
MKhs_2	MBD+	27.62	106.80	68.81	2.48
MKhs_2	MBD-	0.26		27.75	
MKm_1	MBD+	28.67	231.86	71.40	2.71
MKm_1	MBD-	0.12		26.39	
MKm_2	MBD+	33.44	391.40	69.14	2.92
MKm_2	MBD-	0.09		23.66	
MKs_1	MBD+	38.76	174.20	75.92	2.36
MKs_1	MBD-	0.22		32.11	
MKs_2	MBD+	10.01	103.64	51.01	1.84
MKs_2	MBD-	0.10		27.66	
MZh_1	MBD+	24.39	12.09	66.46	1.50
MZh_1	MBD-	2.02		44.35	
MZl_1	MBD+	64.27	6.59	83.03	1.84
MZl_1	MBD-	9.75		45.19	
MZm_1	MBD+	14.92	19.24	50.98	1.41
MZm_1	MBD-	0.78		36.08	
PB25	MBD+	31.84	41.93	40.94	3.70
PB25	MBD-	0.76		11.06	
PB44	MBD+	20.05	11.14	65.65	2.71
PB44	MBD-	1.80		24.25	
PB9	MBD+	22.61	37.87	37.03	2.34
PB9	MBD-	0.60		15.81	
		minimum	6.00	minimum	1.41
		maximum	391.40	maximum	4.62
		average	72.53	average	2.82
		median	41.93	median	2.71

TableS2.3_mt_proportion

Sample	Fraction	Total Reads	mtDNA Uniquely mapped Q0	mtDNA Uniquely mapped Q25	nuDNA Uniquely mapped Q0	nuDNA Uniquely mapped Q25	(N reads mtDNA MBD- / N reads nuDNA MBD-) / (N reads mtDNA MBD+ / N reads nuDNA MBD+)
HSSR	MBD+	43,779,216	12,381	12,380	8,081,030	3,086,948	0.91
HSSR	MBD-	19,393,362	42,430	42,427	17,574,431	11,642,589	
MKb1_1	MBD+	10,461,328	387	363	2,568,020	393,701	2.96
MKb1_1	MBD-	12,872,086	17,089	16,564	8,390,055	6,076,225	
MKb1_2	MBD+	2,385,434	117	116	474,281	119,978	3.10
MKb1_2	MBD-	2,770,052	4,257	4,201	1,816,283	1,400,949	
MKb2_1	MBD+	11,878,035	163	130	310,726	63,605	1.88
MKb2_1	MBD-	11,636,012	18,991	18,545	6,427,106	4,818,528	
MKb2_2	MBD+	2,800,110	54	52	69,117	35,486	2.75
MKb2_2	MBD-	2,989,196	5,316	5,239	1,691,452	1,300,723	
MKhs_1	MBD+	11,628,856	225	212	103,253	23,649	1.08
MKhs_1	MBD-	12,679,846	27,511	26,742	4,091,309	2,756,454	
MKhs_2	MBD+	5,239,477	96	94	16,555	7,080	1.08
MKhs_2	MBD-	2,836,386	7,618	7,519	729,900	526,695	
MKm_1	MBD+	11,966,724	64	58	20,756	8,290	2.79
MKm_1	MBD-	16,177,223	982	946	66,056	48,462	
MKm_2	MBD+	15,533,439	90	89	15,604	7,187	0.55
MKm_2	MBD-	3,069,468	76	73	13,956	10,648	
MKs_1	MBD+	12,949,142	338	316	25,664	9,907	2.82
MKs_1	MBD-	20,004,345	8,040	7,645	128,276	85,109	
MKs_2	MBD+	2,890,744	101	96	4,135	2,210	2.04
MKs_2	MBD-	2,717,680	1,507	1,462	23,327	16,521	
MZh_1	MBD+	7,171,605	50	48	7,478	3,291	23.61
MZh_1	MBD-	8,067,779	827	813	4,761	2,361	
MZl_1	MBD+	8,702,102	3,078	2,757	20,691	8,531	5.80
MZl_1	MBD-	9,004,754	254,683	247,730	370,837	132,143	
MZm_1	MBD+	10,819,017	237	230	24,963	14,288	15.62
MZm_1	MBD-	13,728,406	2,468	2,418	16,216	9,618	
PB25	MBD+	2,107,980	63	57	858,663	744,109	16.24
PB25	MBD-	1,569,088	920	910	816,483	731,666	
PB9	MBD+	1,859,912	197	194	769,747	626,263	8.82
PB9	MBD-	1,538,441	1,717	1,706	737,452	624,321	
						minimum	0.55
						maximum	23.61
						average	6.78
						median	2.82

TableS2.4_genome coverage

Sample	Library	nuDNA coverage (Q25)
EQ26	MBD+	0.0005970
EQ26	MBD-	0.0019836
EQ34	MBD+	0.0001018
EQ34	MBD-	0.0007857
EQ36	MBD+	0.0001022
EQ36	MBD-	0.0006359
EQA	MBD+	0.0000508
EQA	MBD-	0.0030969
EQB	MBD+	0.0000180
EQB	MBD-	0.0000235
EQC	MBD+	0.0000331
EQC	MBD-	0.0000470
EQE	MBD+	0.0000165
EQE	MBD-	0.0000206
EQF	MBD+	0.0000213
EQF	MBD-	0.0003628
HSSR	MBD+	0.0592719
HSSR	MBD-	0.2026680
MKb1_1	MBD+	0.0123614
MKb1_1	MBD-	0.1230580
MKb1_2	MBD+	0.0028261
MKb1_2	MBD-	0.0318464
MKb2_1	MBD+	0.0017618
MKb2_1	MBD-	0.1084837
MKb2_2	MBD+	0.0008387
MKb2_2	MBD-	0.0291845
MKhs_1	MBD+	0.0005857
MKhs_1	MBD-	0.0490238
MKhs_2	MBD+	0.0001402
MKhs_2	MBD-	0.0105957
MKm_1	MBD+	0.0002046
MKm_1	MBD-	0.0009363
MKm_2	MBD+	0.0001551
MKm_2	MBD-	0.0002212
MKs_1	MBD+	0.0001883
MKs_1	MBD-	0.0012954
MKs_2	MBD+	0.0000429
MKs_2	MBD-	0.0003066
MZh_1	MBD+	0.0000590
MZh_1	MBD-	0.0000438
MZh_2	MBD+	0.0000244
MZh_2	MBD-	0.0000049
MZl_1	MBD+	0.0001721
MZl_1	MBD-	0.0033100
MZl_2	MBD+	0.0000206
MZl_2	MBD-	0.0007074
MZm_1	MBD+	0.0002535
MZm_1	MBD-	0.0001851
MZm_2	MBD+	0.0000236
MZm_2	MBD-	0.0000557
PB25	MBD+	0.0564850
PB25	MBD-	0.0326214
PB44	MBD+	0.0217095
PB44	MBD-	0.0222526
PB9	MBD+	0.0456420
PB9	MBD-	0.0274122

Group	Average nuDNA coverage
average EQ MBD+	0.0001176
average EQ MBD-	0.0008695
average HS MBD+	0.0592719
average HS MBD-	0.2026680
average M MBD+	0.0012286
average M MBD-	0.0224536
average PB MBD+	0.0412788
average PB MBD-	0.0274287

TableS2.5_mtDNA per cell

Sample	Library	Size	M	Coverage (Q25)	Genome	mtDNA copies per cell
EQ26	MBD+	16,690	563	0.033732774	mtDNA	113.01
EQ26	MBD+	2,484,515,402	1,483,209	0.000596981	nuDNA	
EQ26	MBD-	16,690	12,766	0.764889155	mtDNA	771.20
EQ26	MBD-	2,484,515,402	4,928,353	0.001983628	nuDNA	
EQ36	MBD+	16,690	59	0.003535051	mtDNA	69.20
EQ36	MBD+	2,484,515,402	253,858	0.000102176	nuDNA	
EQ36	MBD-	16,690	1,014	0.060754943	mtDNA	191.09
EQ36	MBD-	2,484,515,402	1,579,848	0.000635878	nuDNA	
EQA	MBD+	16,690	25	0.001497903	mtDNA	59.02
EQA	MBD+	2,484,515,402	126,116	5.07608E-05	nuDNA	
EQA	MBD-	16,690	3,095	0.185440384	mtDNA	119.76
EQA	MBD-	2,484,515,402	7,694,366	0.003096928	nuDNA	
HSSR	MBD+	16,599	392,532	23.6479306	mtDNA	2.33
HSSR	MBD+	16,599	336,912	20.29712633	mtDNA	
HSSR	MBD-	3,095,693,981	99,620,831	0.032180452	nuDNA	2.38
HSSR	MBD-	3,095,693,981	83,866,952	0.027091487	nuDNA	
HSSR	MBD+	16,599	1,281,793	77.22109766	mtDNA	2.50
HSSR	MBD+	16,599	1,023,394	61.65395506	mtDNA	
HSSR	MBD-	3,095,693,981	345,427,095	0.111583088	nuDNA	2.45
HSSR	MBD-	3,095,693,981	281,971,075	0.091084932	nuDNA	
HSSU	MBD+	16,599	147,462	8.88378818	mtDNA	3.57
HSSU	MBD+	16,599	82,562	4.973914091	mtDNA	
HSSU	MBD-	3,095,693,981	25,124,623	0.008115991	nuDNA	3.66
HSSU	MBD-	3,095,693,981	13,732,079	0.004435865	nuDNA	
MKb1_1	MBD+	16,842	32,603	1.935815224	mtDNA	313.20
MKb1_1	MBD+	3,196,760,833	39,516,453	0.012361404	nuDNA	
MKb1_1	MBD-	16,842	1,095,277	65.03247833	mtDNA	1,056.94
MKb1_1	MBD-	3,196,760,833	393,387,065	0.123058022	nuDNA	
MKb1_2	MBD+	16,842	8,188	0.486165539	mtDNA	344.06
MKb1_2	MBD+	3,196,760,833	9,034,298	0.002826079	nuDNA	
MKb1_2	MBD-	16,842	306,172	18.17907612	mtDNA	1,141.67
MKb1_2	MBD-	3,196,760,833	101,805,287	0.031846388	nuDNA	
MKb2_1	MBD+	16,842	9,134	0.54233464	mtDNA	615.65
MKb2_1	MBD+	3,196,760,833	5,632,109	0.001761817	nuDNA	
MKb2_1	MBD-	16,842	1,318,596	78.29212683	mtDNA	1,443.39
MKb2_1	MBD-	3,196,760,833	346,796,322	0.108483662	nuDNA	
MKb2_2	MBD+	16,842	3,904	0.231801449	mtDNA	552.77
MKb2_2	MBD+	3,196,760,833	2,681,095	0.000838691	nuDNA	
MKb2_2	MBD-	16,842	366,106	21.73767961	mtDNA	1,489.68
MKb2_2	MBD-	3,196,760,833	93,295,722	0.029184455	nuDNA	
MKhs_1	MBD+	16,842	16,760	0.99513122	mtDNA	3,397.81
MKhs_1	MBD+	3,196,760,833	1,872,499	0.000585749	nuDNA	
MKhs_1	MBD-	16,842	1,871,631	111.1287852	mtDNA	4,533.67
MKhs_1	MBD-	3,196,760,833	156,717,351	0.049023796	nuDNA	
MKhs_2	MBD+	16,842	5,808	0.344852155	mtDNA	4,918.43
MKhs_2	MBD+	3,196,760,833	448,277	0.000140229	nuDNA	
MKhs_2	MBD-	16,842	546,528	32.45030281	mtDNA	6,125.19
MKhs_2	MBD-	3,196,760,833	33,871,868	0.010595684	nuDNA	
MKm_1	MBD+	16,842	5,044	0.299489372	mtDNA	2,927.07
MKm_1	MBD+	3,196,760,833	654,167	0.000204634	nuDNA	
MKm_1	MBD-	16,842	70,179	4.166904168	mtDNA	8,900.61
MKm_1	MBD-	3,196,760,833	2,993,186	0.000936318	nuDNA	
MKm_2	MBD+	16,842	6,250	0.371096069	mtDNA	4,785.16
MKm_2	MBD+	3,196,760,833	495,827	0.000155103	nuDNA	
MKm_2	MBD-	16,842	4,709	0.279598623	mtDNA	2,528.06
MKm_2	MBD-	3,196,760,833	707,110	0.000221196	nuDNA	
MKs_1	MBD+	16,842	20,463	1.214998219	mtDNA	12,906.92
MKs_1	MBD+	3,196,760,833	601,857	0.000188271	nuDNA	
MKs_1	MBD-	16,842	404,544	24.01995012	mtDNA	37,083.98
MKs_1	MBD-	3,196,760,833	4,141,197	0.001295435	nuDNA	
MKhs_2	MBD+	16,842	5,808	0.344852155	mtDNA	1,229.61
MKhs_2	MBD+	3,196,760,833	448,277	0.000140229	nuDNA	
MKhs_2	MBD-	16,842	546,528	32.45030281	mtDNA	1,531.30
MKhs_2	MBD-	3,196,760,833	33,871,868	0.010595684	nuDNA	
MZh_1	MBD+	16,842	3,356	0.199263745	mtDNA	6,760.11
MZh_1	MBD+	3,196,760,833	188,458	5.89528E-05	nuDNA	
MZh_1	MBD-	16,842	89,153	5.293492459	mtDNA	241,458.70
MZh_1	MBD-	3,196,760,833	140,165	4.38459E-05	nuDNA	
MZl_1	MBD+	16,842	332,999	19.7719392	mtDNA	229,761.63
MZl_1	MBD+	3,196,760,833	550,189	0.000172108	nuDNA	
MZl_1	MBD-	16,842	20,627,374	1224.757986	mtDNA	740,045.97
MZl_1	MBD-	3,196,760,833	10,581,122	0.003309951	nuDNA	
MZm_1	MBD+	16,842	18,112	1.075406721	mtDNA	8,484.07
MZm_1	MBD+	3,196,760,833	810,417	0.000253512	nuDNA	
MZm_1	MBD-	16,842	168,824	10.02398765	mtDNA	108,334.97
MZm_1	MBD-	3,196,760,833	591,578	0.000185055	nuDNA	
PB25	MBD+	16,898	12,282	0.726831578	mtDNA	25.74
PB25	MBD+	2,308,415,131	130,390,854	0.056485011	nuDNA	
PB25	MBD-	16,898	107,867	6.383418156	mtDNA	391.36
PB25	MBD-	2,308,415,131	75,303,621	0.032621351	nuDNA	
PB44	MBD+	16,898	294	0.017398509	mtDNA	1.60
PB44	MBD+	2,308,415,131	50,114,527	0.021709495	nuDNA	
PB44	MBD-	16,898	83,126	4.919280388	mtDNA	442.13
PB44	MBD-	2,308,415,131	51,368,258	0.022252608	nuDNA	
PB9	MBD+	16,898	37,729	2.232749438	mtDNA	97.84
PB9	MBD+	2,308,415,131	105,360,759	0.045642033	nuDNA	
PB9	MBD-	16,898	165,750	9.808853119	mtDNA	715.66
PB9	MBD-	2,308,415,131	63,278,814	0.027412233	nuDNA	
					**minimum**	**1.60**
					**maximum**	**740,045.97**
					**average**	**32,629.21**
					**median**	**914.07**

ZHENYA	Fraction	mtDNA copies per cell	mtDNA copies per cell liver / other tissue
Heart	MBD+	6,760	34.0
Muscle	MBD+	8,484	27.1
Liver	MBD+	229,762	
Heart	MBD-	241,459	3.1
Muscle	MBD-	108,335	6.8
Liver	MBD-	740,046	
		**min**	3.1
		**max**	34.0
		**average**	17.7

TableS3.1_CpG islands

ID	Fraction	Contig	Total Reads	Coverage CpG islands	Normalized coverage CpG islands	MBD+/MBD- normalized coverage	N reads CpG islands	Average CpGs per read	CpG density MBD+ / MBD-	CpG desnsity / CpG islands density
EQA	MBD+	CpG island	8,894,455	0.000540082	0.00035	0.09	469	0.1034	2.76	30.13
EQA	MBD-	CpG island	5,776,769	0.003828062	0.00383		4,425	0.0375		
EQ26	MBD+	CpG island	1,545,555	0.009946004	0.00148	0.88	5,075	0.1415	5.39	6.10
EQ26	MBD-	CpG island	230,002	0.001674115	0.00167		1,498	0.0262		
EQ34	MBD+	CpG island	989,551	0.001728083	0.00142	1.62	1,033	0.1335	4.36	2.68
EQ34	MBD-	CpG island	810,958	0.000872016	0.00087		1,044	0.0306		
EQ36	MBD+	CpG island	924,313	0.001810958	0.00168	2.70	911	0.1284	4.47	1.65
EQ36	MBD-	CpG island	855,798	0.000620896	0.00062		757	0.0287		
HSSR	MBD+	CpGIsland	43,779,216	0.365698804	0.16200	0.20	88,693	0.0485	1.21	6.05
HSSR	MBD-	CpGIsland	19,393,362	0.806191386	0.80619		251,844	0.0399		

minimum	1.65
maximum	30.13
average	9.32
median	6.05

TableS3.2_Non Unique reads

Sample	Library	N reads nuDNA Q0 (duplicates included)	N unique hits	N Non-unique hits	% N not uniquely mapped reads	MDB+ / MBD - N not uniquely mapped reads
EQ26	MBD+	65,036	12,452	52,584	80.85	4.58
EQ26	MBD-	85,669	70,546	15,123	17.65	
EQ34	MBD+	15,731	2,697	13,034	82.86	3.04
EQ34	MBD-	52,612	38,293	14,319	27.22	
EQ36	MBD+	13,373	2,318	11,055	82.67	3.50
EQ36	MBD-	41,413	31,629	9,784	23.63	
EQA	MBD+	10,316	2,038	8,278	80.24	4.34
EQA	MBD-	156,757	127,794	28,963	18.48	
HSSR	MBD+	7,870,985	2,921,566	4,949,419	62.88	1.85
HSSR	MBD-	17,378,429	11,467,585	5,910,844	34.01	
MKb1_1	MBD+	2,489,937	371,216	2,118,721	85.09	3.08
MKb1_1	MBD-	8,374,224	6,061,916	2,312,308	27.61	
MKb1_2	MBD+	232,093	56,334	175,759	75.73	3.16
MKb1_2	MBD-	1,503,187	1,142,914	360,273	23.97	
MKb2_1	MBD+	303,333	59,735	243,598	80.31	3.20
MKb2_1	MBD-	6,409,472	4,802,769	1,606,703	25.07	
MKb2_2	MBD+	50,668	24,897	25,771	50.86	2.10
MKb2_2	MBD-	1,492,012	1,131,396	360,616	24.17	
MKhs_1	MBD+	94,060	19,651	74,409	79.11	2.42
MKhs_1	MBD-	4,084,118	2,750,259	1,333,859	32.66	
MKhs_2	MBD+	7,400	2,667	4,733	63.96	2.18
MKhs_2	MBD-	641,243	452,718	188,525	29.40	
MKm_1	MBD+	19,413	7,674	11,739	60.47	2.30
MKm_1	MBD-	65,015	47,901	17,114	26.32	
MKm_2	MBD+	10,089	4,305	5,784	57.33	2.47
MKm_2	MBD-	13,426	10,304	3,122	23.25	
MKs_1	MBD+	23,997	9,077	14,920	62.17	1.86
MKs_1	MBD-	126,837	84,332	42,505	33.51	
MZh_1	MBD+	6,021	2,663	3,358	55.77	1.11
MZh_1	MBD-	4,082	2,034	2,048	50.17	
MZl_1	MBD+	17,153	6,893	10,260	59.81	0.93
MZl_1	MBD-	368,817	131,122	237,695	64.45	
MZm_1	MBD+	20,873	12,261	8,612	41.26	1.03
MZm_1	MBD-	15,421	9,243	6,178	40.06	
PB25	MBD+	827,556	714,143	113,413	13.70	1.31
PB25	MBD-	813,880	729,049	84,831	10.42	
PB44	MBD+	860,521	368,138	492,383	57.22	2.49
PB44	MBD-	655,810	505,391	150,419	22.94	
PB9	MBD+	754,424	611,460	142,964	18.95	1.23
PB9	MBD-	735,791	622,587	113,204	15.39	
					min	0.93
					max	4.58
					average	2.41
					median	2.36

TableS3.3_Repeated Elements

Sample	Sample	Contig	Fraction	N Bases Sample	N Bases Contig	Coverage	Relative Repeated elements coverage	Relative Repeated Elements coverage MBD+ / MBD-
EQ26	EQ26	Repeats	MBD+	3,806,530	1,811,090,450	0.002102	3.25	4.34
EQ26	EQ26	NonRepeats	MBD+	884,631	1,369,484,561	0.000646		
EQ26	EQ26	Repeats	MBD-	2,912,328	1,811,090,450	0.001608	0.75	
EQ26	EQ26	NonRepeats	MBD-	2,937,856	1,369,484,561	0.002145		
EQ34	EQ34	Repeats	MBD+	1,111,840	1,811,090,450	0.000614	5.27	5.98
EQ34	EQ34	NonRepeats	MBD+	159,466	1,369,484,561	0.000116		
EQ34	EQ34	Repeats	MBD-	1,404,581	1,811,090,450	0.000776	0.88	
EQ34	EQ34	NonRepeats	MBD-	1,204,916	1,369,484,561	0.000880		
EQ36	EQ36	Repeats	MBD+	1,053,801	1,811,090,450	0.000582	5.48	6.85
EQ36	EQ36	NonRepeats	MBD+	145,479	1,369,484,561	0.000106		
EQ36	EQ36	Repeats	MBD-	1,042,496	1,811,090,450	0.000576	0.80	
EQ36	EQ36	NonRepeats	MBD-	986,411	1,369,484,561	0.000720		
EQA	EQA	Repeats	MBD+	409,736	1,811,090,450	0.000226	3.08	4.49
EQA	EQA	NonRepeats	MBD+	100,536	1,369,484,561	0.000073		
EQA	EQA	Repeats	MBD-	4,324,658	1,811,090,450	0.002388	0.69	
EQA	EQA	NonRepeats	MBD-	4,766,846	1,369,484,561	0.003481		
HSSR	HSSR	Repeats	MBD+	197,097,136	6,574,197,005	0.029980	0.63	1.73
HSSR	HSSR	NonRepeats	MBD+	58,875,328	1,242,291,910	0.047393		
HSSR	HSSR	Repeats	MBD-	429,457,237	6,574,197,005	0.065325	0.37	
HSSR	HSSR	NonRepeats	MBD-	221,359,394	1,242,291,910	0.178186		
MKb1_1	MKb1_1	Repeats	MBD+	21,780,036	2,527,360,781	0.008618	0.79	1.05
MKb1_1	MKb1_1	NonRepeats	MBD+	16,647,212	1,528,205,064	0.010893		
MKb1_1	MKb1_1	Repeats	MBD-	245,904,563	2,527,360,781	0.097297	0.75	
MKb1_1	MKb1_1	NonRepeats	MBD-	198,201,113	1,528,205,064	0.129695		
MKb1_2	MKb1_2	Repeats	MBD+	3,755,865	2,527,360,781	0.001486	0.92	1.17
MKb1_2	MKb1_2	NonRepeats	MBD+	2,478,952	1,528,205,064	0.001622		
MKb1_2	MKb1_2	Repeats	MBD-	56,676,232	2,527,360,781	0.022425	0.79	
MKb1_2	MKb1_2	NonRepeats	MBD-	43,650,518	1,528,205,064	0.028563		
MKb2_1	MKb2_1	Repeats	MBD+	5,432,335	2,527,360,781	0.002149	0.68	0.89
MKb2_1	MKb2_1	NonRepeats	MBD+	4,814,759	1,528,205,064	0.003151		
MKb2_1	MKb2_1	Repeats	MBD-	217,562,660	2,527,360,781	0.086083	0.77	
MKb2_1	MKb2_1	NonRepeats	MBD-	171,931,278	1,528,205,064	0.112505		
MKb2_2	MKb2_2	Repeats	MBD+	1,697,047	2,527,360,781	0.000671	0.77	0.97
MKb2_2	MKb2_2	NonRepeats	MBD+	1,335,208	1,528,205,064	0.000874		
MKb2_2	MKb2_2	Repeats	MBD-	55,609,561	2,527,360,781	0.022003	0.79	
MKb2_2	MKb2_2	NonRepeats	MBD-	42,546,276	1,528,205,064	0.027841		
MKhs_1	MKhs_1	Repeats	MBD+	1,933,970	2,527,360,781	0.000765	0.69	0.96
MKhs_1	MKhs_1	NonRepeats	MBD+	1,701,515	1,528,205,064	0.001113		
MKhs_1	MKhs_1	Repeats	MBD-	105,221,449	2,527,360,781	0.041633	0.71	
MKhs_1	MKhs_1	NonRepeats	MBD-	89,031,331	1,528,205,064	0.058259		
MKhs_2	MKhs_2	Repeats	MBD+	280,439	2,527,360,781	0.000111	0.98	1.29
MKhs_2	MKhs_2	NonRepeats	MBD+	172,548	1,528,205,064	0.000113		
MKhs_2	MKhs_2	Repeats	MBD-	20,519,466	2,527,360,781	0.008119	0.76	
MKhs_2	MKhs_2	NonRepeats	MBD-	16,241,223	1,528,205,064	0.010628		
MKm_1	MKm_1	Repeats	MBD+	648,228	2,527,360,781	0.000256	0.77	1.10
MKm_1	MKm_1	NonRepeats	MBD+	511,335	1,528,205,064	0.000335		
MKm_1	MKm_1	Repeats	MBD-	2,055,304	2,527,360,781	0.000813	0.70	
MKm_1	MKm_1	NonRepeats	MBD-	1,776,488	1,528,205,064	0.001162		
MKm_2	MKm_2	Repeats	MBD+	387,022	2,527,360,781	0.000153	0.91	1.21
MKm_2	MKm_2	NonRepeats	MBD+	256,447	1,528,205,064	0.000168		
MKm_2	MKm_2	Repeats	MBD-	481,503	2,527,360,781	0.000191	0.75	
MKm_2	MKm_2	NonRepeats	MBD-	387,508	1,528,205,064	0.000254		
MKs_1	MKs_1	Repeats	MBD+	663,965	2,527,360,781	0.000263	0.87	1.22
MKs_1	MKs_1	NonRepeats	MBD+	464,123	1,528,205,064	0.000304		
MKs_1	MKs_1	Repeats	MBD-	3,026,639	2,527,360,781	0.001198	0.71	
MKs_1	MKs_1	NonRepeats	MBD-	2,570,743	1,528,205,064	0.001682		
MZh_1	MZh_1	Repeats	MBD+	164,676	2,527,360,781	0.000065	1.04	1.57
MZh_1	MZh_1	NonRepeats	MBD+	95,527	1,528,205,064	0.000063		
MZh_1	MZh_1	Repeats	MBD-	98,957	2,527,360,781	0.000039	0.66	
MZh_1	MZh_1	NonRepeats	MBD-	90,309	1,528,205,064	0.000059		
MZl_1	MZl_1	Repeats	MBD+	387,860	2,527,360,781	0.000153	0.75	1.09
MZl_1	MZl_1	NonRepeats	MBD+	313,478	1,528,205,064	0.000205		
MZl_1	MZl_1	Repeats	MBD-	5,591,317	2,527,360,781	0.002212	0.68	
MZl_1	MZl_1	NonRepeats	MBD-	4,940,508	1,528,205,064	0.003233		
MZm_1	MZm_1	Repeats	MBD+	599,222	2,527,360,781	0.000237	0.82	1.24
MZm_1	MZm_1	NonRepeats	MBD+	442,484	1,528,205,064	0.000290		
MZm_1	MZm_1	Repeats	MBD-	392,951	2,527,360,781	0.000155	0.66	
MZm_1	MZm_1	NonRepeats	MBD-	360,292	1,528,205,064	0.000236		
PB25	PB25	Repeats	MBD+	31,963,696	1,997,874,316	0.015999	0.31	0.76
PB25	PB25	NonRepeats	MBD+	77,143,763	1,496,108,866	0.051563		
PB25	PB25	Repeats	MBD-	27,013,999	1,997,874,316	0.013521	0.41	
PB25	PB25	NonRepeats	MBD-	49,422,943	1,496,108,866	0.033034		
PB44	PB44	Repeats	MBD+	20,326,376	1,997,874,316	0.010174	0.64	1.70
PB44	PB44	NonRepeats	MBD+	23,601,442	1,496,108,866	0.015775		
PB44	PB44	Repeats	MBD-	17,850,462	1,997,874,316	0.008935	0.38	
PB44	PB44	NonRepeats	MBD-	35,329,209	1,496,108,866	0.023614		
PB9	PB9	Repeats	MBD+	31,852,254	1,997,874,316	0.015943	0.38	0.81
PB9	PB9	NonRepeats	MBD+	63,130,459	1,496,108,866	0.042196		
PB9	PB9	Repeats	MBD-	25,154,980	1,997,874,316	0.012591	0.47	
PB9	PB9	NonRepeats	MBD-	40,496,036	1,496,108,866	0.027068		
							min	0.76
							max	6.85
							average	2.08
							median	1.21

TableS3.4_no CpG reads

Sample	Library	Ntot	N reads No-CpGs	% reads No-CpGs
EQ26	MBD+	12,467	359	2.88
EQ26	MBD-	70,561	36,367	51.54
EQ34	MBD+	2,700	230	8.52
EQ34	MBD-	38,301	20,844	54.42
EQ36	MBD+	2,325	164	7.05
EQ36	MBD-	31,633	18,145	57.36
EQA	MBD+	2,041	631	30.92
EQA	MBD-	127,821	56,369	44.10
HSSR	MBD+	2,923,436	1,352,845	46.28
HSSR	MBD-	11,469,740	5,806,157	50.62
MKb1_1	MBD+	372,725	49,889	13.38
MKb1_1	MBD-	6,064,924	3,055,142	50.37
MKb1_2	MBD+	56,553	10,045	17.76
MKb1_2	MBD-	1,143,569	590,194	51.61
MKb2_1	MBD+	59,822	11,841	19.79
MKb2_1	MBD-	4,805,331	2,438,052	50.74
MKb2_2	MBD+	24,916	5,835	23.42
MKb2_2	MBD-	1,132,076	597,201	52.75
MKhs_1	MBD+	19,689	5,004	25.42
MKhs_1	MBD-	2,751,525	1,331,259	48.38
MKhs_2	MBD+	2,672	834	31.21
MKhs_2	MBD-	452,970	229,760	50.72
MKm_1	MBD+	7,681	2,278	29.66
MKm_1	MBD-	47,930	24,998	52.16
MKm_2	MBD+	4,310	1,630	37.82
MKm_2	MBD-	10,311	5,618	54.49
MKs_1	MBD+	9,087	3,611	39.74
MKs_1	MBD-	84,371	46,350	54.94
MZh_1	MBD+	2,664	1,300	48.80
MZh_1	MBD-	2,038	918	45.04
MZl_1	MBD+	6,899	3,188	46.21
MZl_1	MBD-	131,278	58,580	44.62
MZm_1	MBD+	12,269	5,916	48.22
MZm_1	MBD-	9,259	3,877	41.87
PB25	MBD+	714,331	10,191	1.43
PB25	MBD-	729,183	300,046	41.15
PB44	MBD+	368,682	3,644	0.99
PB44	MBD-	505,586	124,649	24.65
PB9	MBD+	611,655	19,696	3.22
PB9	MBD-	622,807	268,876	43.17

		% reads No-CpGs MBD+
**Equids**	min	2.88
	max	30.92
	average	12.34
**Saqqaq**	min	46.28
	max	46.28
	avg	46.28
**Mammoth**	min	13.38
	max	48.80
	avg	31.79
**Polar Bear**	min	0.99
	max	3.22
	avg	1.88

TableS3.5 Ms Saqqaq

			Ms					
Sample	Library	Threshold density	Minimum	1st quantile	Median	Mean	2nd quantile	Maximum
HSSR	MBD+	50	0.000	0.000	0.019	0.029	0.050	0.736
HSSR	MBD-	50	0.000	0.000	0.015	0.023	0.037	0.736
HSSR	MBD+	60	0.000	0.012	0.032	0.034	0.056	0.736
HSSR	MBD-	60	0.000	0.000	0.017	0.028	0.048	0.736

TableS3.6_CpG.CpT

Sample	MBD	CG>TG	CG>CG	CG>AG	CG>GG	CA>TA	CT>TT	CC>TC	CN>TN	(CG>TG)/(CN>TN)	(CG>TG)/(CN>TN) MBD+/MBD-
EQA	MBD+	26	536	2	0	26	29	35	116	0.2241	6.31
EQA	MBD-	278	4,180	12	18	2,598	3,060	1,890	7,826	0.0355	
EQ26	MBD+	212	6,562	13	6	254	284	408	1,158	0.1831	7.51
EQ26	MBD-	108	1,665	1	3	1,585	1,803	932	4,428	0.0244	
EQ34	MBD+	15	1,281	1	0	11	10	11	47	0.3191	8.26
EQ34	MBD-	17	941	3	5	139	162	122	440	0.0386	
EQ36	MBD+	28	1,036	0	3	32	39	46	145	0.1931	7.75
EQ36	MBD-	25	676	0	3	359	367	252	1,003	0.0249	
MKm_2	MBD+	6	603	2	6	43	36	29	114	0.0526	0.96
MKm_2	MBD-	6	124	0	1	41	46	16	109	0.0550	
MKs_2	MBD+	5	169	0	1	20	17	12	54	0.0926	1.62
MKs_2	MBD-	13	158	1	0	84	80	50	227	0.0573	
MKhs_2	MBD+	11	791	4	3	32	36	24	103	0.1068	2.20
MKhs_2	MBD-	415	7,465	53	42	2,988	3,050	2,108	8,561	0.0485	
MKb2_2	MBD+	98	3,446	10	8	214	240	156	708	0.1384	3.60
MKb2_2	MBD-	877	14,272	123	85	8,897	8,514	4,494	22,782	0.0385	
MKb1_2	MBD+	570	12,837	345	366	726	953	843	3,092	0.1843	4.20
MKb1_2	MBD-	936	15,798	140	103	7,892	7,916	4,588	21,332	0.0439	
MKb2_1	MBD+	265	11,932	42	64	541	520	506	1,832	0.1447	3.46
MKb2_1	MBD-	4,369	73,542	578	519	38,732	38,273	23,094	104,468	0.0418	
MKb1_1	MBD+	3,404	88,370	2,417	2,926	3,207	5,277	5,236	17,124	0.1988	3.02
MKb1_1	MBD-	14,216	84,834	10,443	10,967	65,083	79,129	57,220	215,648	0.0659	
MKhs_1	MBD+	112	7,527	17	47	136	143	173	564	0.1986	4.10
MKhs_1	MBD-	2,438	63,122	395	427	16,666	16,344	14,893	50,341	0.0484	
MKm_1	MBD+	30	2,114	5	6	33	61	50	174	0.1724	1.95
MKm_1	MBD-	54	1,044	6	2	192	220	146	612	0.0882	
MKs_1	MBD+	30	1,281	1	3	67	65	77	239	0.1255	2.80
MKs_1	MBD-	73	1,759	11	14	520	588	447	1,628	0.0448	
PB25	MBD+	9,224	85,796	8,562	11,346	7,568	13,014	19,002	48,808	0.1890	3.19
PB25	MBD-	1,479	14,191	1,417	1,476	6,772	10,072	6,651	24,974	0.0592	
PB44	MBD+	6,977	57,760	6,061	10,162	2,982	5,243	11,861	27,063	0.2578	3.78
PB44	MBD-	920	23,477	511	640	3,161	4,449	4,977	13,507	0.0681	
PB9	MBD+	3,362	105,794	2,910	3,950	4,114	5,953	7,666	21,095	0.1594	2.74
PB9	MBD-	2,220	6,197	2,108	1,957	10,971	15,797	9,204	38,192	0.0581	
MZh_1	MBD+	5	114	1	1	17	24	12	58	0.0862	3.19
MZh_1	MBD-	1	113	2	4	14	13	9	37	0.0270	
MZl_1	MBD+	12	306	2	2	52	63	40	167	0.0719	1.71
MZl_1	MBD-	184	3,034	14	12	1,475	1,734	977	4,370	0.0421	
MZm_1	MBD+	8	425	2	4	70	98	59	235	0.0340	0.58
MZm_1	MBD-	10	513	6	2	46	66	49	171	0.0585	
HSSR	MBD+	2,361	252,940	255	306	9,064	8,532	11,703	31,660	0.0746	1.37
HSSR	MBD-	6,274	511,100	727	615	35,026	31,062	42,579	114,941	0.0546	
										min	0.58
										max	8.26
										average	3.49
										median	3.10

TableS3.7_Number of collapsed r

			Collapsed Reads			
			mtDNA		nuDNA	
Sample	Fraction	Total Reads	Uniquely mapped Q0	Uniquely mapped Q25	Uniquely mapped Q0	Uniquely mapped Q25
EQ26	MBD+	1,545,555	5	5	65,036	12,467
EQ26	MBD-	230,002	192	190	85,669	70,561
EQ34	MBD+	989,551	0	0	15,731	2,700
EQ34	MBD-	810,958	4	4	52,612	38,301
EQ36	MBD+	924,313	1	1	13,373	2,325
EQ36	MBD-	855,798	22	22	41,413	31,633
EQA	MBD+	8,894,455	1	1	10,316	2,041
EQA	MBD-	5,776,769	48	48	156,757	127,821
EQB	MBD+	10,136,112	3	3	4,104	942
EQB	MBD-	7,000,618	2	2	2,797	1,303
EQC	MBD+	10,509,471	4	4	6,450	1,661
EQC	MBD-	7,065,549	1	1	4,173	2,667
EQE	MBD+	8,435,891	0	0	3,526	855
EQE	MBD-	9,195,063	1	1	2,568	1,603
EQF	MBD+	10,060,504	0	0	3,628	1,078
EQF	MBD-	7,550,930	93	93	20,181	16,329
HSSR	MBD+	43,779,216	11,720	11,719	7,870,985	2,923,436
HSSR	MBD-	19,393,362	41,760	41,758	17,378,429	11,469,740
MKb1_1	MBD+	10,461,328	360	337	2,489,937	372,725
MKb1_1	MBD-	12,872,086	17,030	16,509	8,374,224	6,064,924
MKb1_2	MBD+	2,385,434	69	68	232,093	56,553
MKb1_2	MBD-	2,770,052	3,324	3,271	1,503,187	1,143,569
MKb2_1	MBD+	11,878,035	141	110	303,333	59,822
MKb2_1	MBD-	11,636,012	18,899	18,458	6,409,472	4,805,331
MKb2_2	MBD+	2,800,110	32	30	50,668	24,916
MKb2_2	MBD-	2,989,196	4,531	4,455	1,492,012	1,132,076
MKhs_1	MBD+	11,628,856	161	149	94,060	19,689
MKhs_1	MBD-	12,679,846	27,430	26,666	4,084,118	2,751,525
MKhs_2	MBD+	5,239,477	21	21	7,400	2,672
MKhs_2	MBD-	2,836,386	5,538	5,441	641,243	452,970
MKm_1	MBD+	11,966,724	48	43	19,413	7,681
MKm_1	MBD-	16,177,223	961	928	65,015	47,930
MKm_2	MBD+	15,533,439	49	49	10,089	4,310
MKm_2	MBD-	3,069,468	70	67	13,426	10,311
MKs_1	MBD+	12,949,142	316	296	23,997	9,087
MKs_1	MBD-	20,004,345	8,022	7,627	126,837	84,371
MKs_2	MBD+	2,890,744	83	78	3,277	1,717
MKs_2	MBD-	2,717,680	1,446	1,401	22,037	15,583
MZh_1	MBD+	7,171,605	31	29	6,021	2,664
MZh_1	MBD-	8,067,779	737	724	4,082	2,038
MZh_2	MBD+	2,035,979	4	4	882	505
MZh_2	MBD-	903,182	51	51	290	162
MZl_1	MBD+	8,702,102	2,808	2,499	17,153	6,899
MZl_1	MBD-	9,004,754	253,458	246,537	368,817	131,278
MZl_2	MBD+	2,516,242	526	517	1,187	423
MZl_2	MBD-	1,901,793	43,626	42,895	60,427	21,078
MZm_1	MBD+	10,819,017	176	172	20,873	12,269
MZm_1	MBD-	13,728,406	2,457	2,407	15,421	9,259
MZm_2	MBD+	2,135,223	16	15	529	283
MZm_2	MBD-	3,103,006	523	519	2,586	1,653
PB25	MBD+	2,107,980	59	53	827,556	714,331
PB25	MBD-	1,569,088	911	901	813,880	729,183
PB44	MBD+	1,590,587	6	5	860,521	368,682
PB44	MBD-	1,692,855	712	690	655,810	505,586
PB9	MBD+	1,859,912	186	183	754,424	611,655
PB9	MBD-	1,538,441	1,713	1,702	735,791	622,807

TableS4.1_Len CpG CG >2,000

Sample	Library	Nreads	avgLen	avgLn MBD+ - avgLen MBD-	sdLen	minLen	lowLen	midLen	topLen	maxLen	avgGC	avgGC MBD+ / avgGC MBD-	sdGC	minGC	lowGC	midGC	topGC	maxGC	avgCpG	avgCpG MBD+ / avgCpG MBD-	sdCpG	minCpG	lowCpG	midCpG	topCpG	maxCpG
EQ26	MBD+	12,466	115.47	45.67	39.4762	34	88	113	140	234	0.6262	1.46	0.0820	0.3471	0.5882	0.6316	0.6754	0.7964	0.1415	5.39	0.0665	0.0000	0.1017	0.1379	0.1778	0.3333
EQ26	MBD-	70,560	69.80		24.5144	29	52	66	84	141	0.4288		0.1094	0.2154	0.3478	0.4202	0.5000	0.6944	0.0262		0.0376	0.0000	0.0000	0.0129	0.0400	0.1644
EQ34	MBD+	2,699	92.14	41.17	41.0345	25	64	85	112	229	0.6063	1.33	0.1014	0.3091	0.5580	0.6190	0.6667	0.8205	0.1335	4.36	0.0847	0.0000	0.0714	0.1351	0.1856	0.3590
EQ34	MBD-	38,300	50.97		17.1994	25	38	48	60	105	0.4543		0.1138	0.2246	0.3714	0.4483	0.5319	0.7308	0.0306		0.0464	0.0000	0.0000	0.0000	0.0488	0.2000
EQ36	MBD+	2,324	105.48	55.55	45.3997	25	73	101	132	234	0.6005	1.37	0.0921	0.3094	0.5624	0.6120	0.6538	0.8070	0.1284	4.47	0.0767	0.0000	0.0800	0.1300	0.1754	0.3254
EQ36	MBD-	31,632	49.94		17.5906	25	38	46	58	110	0.4384		0.1146	0.2105	0.3542	0.4297	0.5152	0.7200	0.0287		0.0452	0.0000	0.0000	0.0000	0.0476	0.2032
EQA	MBD+	2,040	58.33	-1.83	32.7125	24	33	50	73	162	0.5547	1.15	0.1624	0.2108	0.4348	0.5600	0.6776	0.8925	0.1034	2.76	0.1118	0.0000	0.0000	0.0769	0.1639	0.4733
EQA	MBD-	127,820	60.16		23.6274	26	43	55	72	136	0.4815		0.1119	0.2576	0.4000	0.4750	0.5577	0.7586	0.0375		0.0511	0.0000	0.0000	0.0263	0.0541	0.2308
HSSR	MBD+	2,923,435	58.59	5.06	25.9706	25	39	52	72	138	0.4961	1.01	0.1282	0.2500	0.4000	0.4872	0.5784	0.8378	0.0485	1.21	0.0774	0.0000	0.0000	0.0227	0.0625	0.3778
HSSR	MBD-	11,469,739	53.53		22.9235	25	36	48	65	129	0.4921		0.1214	0.2549	0.4035	0.4857	0.5714	0.8182	0.0399		0.0653	0.0000	0.0000	0.0000	0.0556	0.3256
MKb1_1	MBD+	372,724	99.01	34.26	45.7377	29	64	92	125	239	0.5142	1.18	0.1218	0.2430	0.4314	0.5119	0.5972	0.8298	0.0961	3.69	0.0863	0.0000	0.0351	0.0808	0.1364	0.4400
MKb1_1	MBD-	6,064,923	64.74		26.4893	28	46	59	78	151	0.4366		0.0965	0.2353	0.3684	0.4324	0.5000	0.6842	0.0261		0.0376	0.0000	0.0000	0.0000	0.0417	0.1622
MKb1_2	MBD+	56,552	83.75	10.62	27.4326	31	62	84	105	136	0.4928	1.22	0.1184	0.2267	0.4110	0.4940	0.5802	0.7949	0.0854	4.02	0.0780	0.0000	0.0260	0.0734	0.1280	0.3824
MKb1_2	MBD-	1,143,568	73.12		26.2175	31	52	69	91	134	0.4055		0.0911	0.2143	0.3412	0.4016	0.4656	0.6341	0.0212		0.0299	0.0000	0.0000	0.0000	0.0351	0.1250
MKb2_1	MBD+	59,821	87.75	15.78	41.8768	26	55	81	113	217	0.4919	1.16	0.1268	0.2200	0.4074	0.4835	0.5673	0.8302	0.0793	3.45	0.0830	0.0000	0.0230	0.0625	0.1073	0.4286
MKb2_1	MBD-	4,805,330	71.97		30.1808	28	49	66	89	161	0.4238		0.0954	0.2222	0.3571	0.4200	0.4857	0.6667	0.0230		0.0332	0.0000	0.0000	0.0000	0.0370	0.1429
MKb2_2	MBD+	24,915	78.50	6.64	28.5026	30	55	76	101	135	0.4655	1.14	0.1116	0.2154	0.3917	0.4648	0.5345	0.7447	0.0659	3.20	0.0645	0.0000	0.0169	0.0556	0.0980	0.2857
MKb2_2	MBD-	1,132,075	71.86		26.2645	30	51	68	90	134	0.4093		0.0917	0.2128	0.3451	0.4063	0.4706	0.6364	0.0206		0.0293	0.0000	0.0000	0.0000	0.0345	0.1231
MKhs_1	MBD+	19,688	78.65	21.81	47.1439	25	45	65	97	245	0.5331	1.13	0.1634	0.2241	0.4167	0.5059	0.6333	0.9211	0.1101	3.38	0.1388	0.0000	0.0000	0.0571	0.1429	0.5532
MKhs_1	MBD-	2,751,524	56.84		26.0761	26	38	50	68	149	0.4711		0.1034	0.2569	0.4000	0.4658	0.5360	0.7400	0.0326		0.0473	0.0000	0.0000	0.0179	0.0513	0.2162
MKhs_2	MBD+	2,671	70.58	7.22	27.7027	29	47	67	90	135	0.4969	1.13	0.1495	0.2179	0.3889	0.4805	0.5862	0.8684	0.0862	3.36	0.1182	0.0000	0.0000	0.0435	0.1134	0.5268
MKhs_2	MBD-	452,969	63.35		25.5967	29	43	58	78	132	0.4394		0.0963	0.2373	0.3714	0.4359	0.5000	0.6829	0.0257		0.0364	0.0000	0.0000	0.0000	0.0426	0.1579
MKm_1	MBD+	7,680	79.37	17.65	39.3545	24	50	72	101	202	0.502	1.19	0.1530	0.2000	0.3913	0.4872	0.6000	0.8732	0.0853	3.29	0.1150	0.0000	0.0000	0.0421	0.1132	0.5000
MKm_1	MBD-	47,929	61.72		19.1777	28	48	59	72	120	0.4236		0.1022	0.2206	0.3500	0.4167	0.4894	0.6889	0.0260		0.0394	0.0000	0.0000	0.0000	0.0400	0.1724
MKm_2	MBD+	4,309	75.95	9.22	26.9377	28	54	73	96	135	0.4514	1.15	0.1285	0.1842	0.3600	0.4444	0.5294	0.7953	0.0533	2.57	0.0799	0.0000	0.0000	0.0274	0.0732	0.4090
MKm_2	MBD-	10,310	66.73		20.1297	33	52	64	78	124	0.3939		0.0955	0.2027	0.3261	0.3878	0.4583	0.6292	0.0207		0.0301	0.0000	0.0000	0.0000	0.0345	0.1224
MKs_1	MBD+	9,086	60.57	12.05	31.2869	24	38	53	74	171	0.4695	1.06	0.1401	0.1923	0.3738	0.4565	0.5484	0.8462	0.0627	2.07	0.0943	0.0000	0.0000	0.0323	0.0833	0.4659
MKs_1	MBD-	84,370	48.51		17.2765	25	37	45	57	106	0.4411		0.1081	0.2200	0.3659	0.4340	0.5085	0.7407	0.0302		0.0495	0.0000	0.0000	0.0000	0.0488	0.2308
MKs_2	MBD+	1,716	63.14	4.15	25.7963	27	42	58	79	132	0.4374	1.09	0.1237	0.1752	0.3500	0.4303	0.5167	0.7629	0.0506	2.33	0.0709	0.0000	0.0000	0.0276	0.0759	0.3099
MKs_2	MBD-	15,582	58.98		22.2003	28	43	54	70	127	0.402		0.0959	0.2045	0.3333	0.3966	0.4667	0.6452	0.0217		0.0328	0.0000	0.0000	0.0000	0.0385	0.1379
MZh_1	MBD+	2,663	57.27	-0.21	26.9253	24	37	51	71	142	0.4609	0.99	0.1223	0.2167	0.3810	0.4524	0.5263	0.8468	0.0374	0.93	0.0700	0.0000	0.0000	0.0000	0.0493	0.3750
MZh_1	MBD-	2,037	57.48		34.2539	24	34	49	69	176	0.4655		0.1237	0.2314	0.3768	0.4545	0.5385	0.8400	0.0401		0.0701	0.0000	0.0000	0.0159	0.0526	0.3945
MZl_1	MBD+	6,898	65.68	-14.24	33.1126	25	42	58	80	181	0.4452	1.05	0.1134	0.2222	0.3684	0.4348	0.5102	0.7943	0.0343	1.28	0.0600	0.0000	0.0000	0.0171	0.0465	0.3243
MZl_1	MBD-	131,277	79.92		38.1731	28	51	70	100	194	0.4243		0.0929	0.2407	0.3604	0.4154	0.4805	0.6812	0.0268		0.0391	0.0000	0.0000	0.0179	0.0408	0.1667
MZm_1	MBD+	12,268	57.00	-4.24	26.0464	25	38	50	69	147	0.467	0.98	0.1119	0.2418	0.3902	0.4600	0.5341	0.7895	0.0350	0.76	0.0588	0.0000	0.0000	0.0146	0.0513	0.3063
MZm_1	MBD-	9,258	61.24		31.5552	24	39	53	75	166	0.4774		0.1222	0.2500	0.3939	0.4643	0.5385	0.8772	0.0463		0.0781	0.0000	0.0000	0.0249	0.0571	0.4231
PB25	MBD+	714,330	172.64	70.06	90.3444	43	104	152	223	437	0.5672	1.32	0.0903	0.3623	0.5028	0.5703	0.6333	0.7600	0.0849	3.35	0.0537	0.0000	0.0461	0.0741	0.1129	0.2564
PB25	MBD-	729,182	102.57		60.1037	32	61	86	126	322	0.4287		0.1083	0.2151	0.3488	0.4189	0.5000	0.6912	0.0253		0.0337	0.0000	0.0000	0.0165	0.0375	0.1515
PB44	MBD+	368,681	134.62	33.39	64.6116	39	90	120	164	353	0.6358	1.28	0.0825	0.3727	0.5933	0.6475	0.6891	0.8000	0.1274	3.27	0.0612	0.0000	0.0845	0.1205	0.1613	0.3088
PB44	MBD-	505,585	101.23		53.5881	36	64	87	122	296	0.4986		0.0969	0.2953	0.4273	0.4961	0.5676	0.7213	0.0389		0.0418	0.0000	0.0079	0.0292	0.0548	0.1923
PB9	MBD+	611,654	166.67	65.52	83.2414	42	106	152	207	422	0.5408	1.30	0.0996	0.3214	0.4678	0.5443	0.6154	0.7463	0.0775	3.24	0.0519	0.0000	0.0399	0.0674	0.1053	0.2400
PB9	MBD-	622,806	101.15		54.9277	33	62	87	125	300	0.4147		0.1071	0.2020	0.3371	0.4046	0.4853	0.6786	0.0239		0.0324	0.0000	0.0000	0.0152	0.0357	0.1458

**average Equids**	35.14
**average Mammoth**	9.29
**average Polar Bear**	56.32
**Saqqaq**	5.06
**Overall Average**	20.73

99.4	Difference in the verage length of endogenous DNA inserts in MBD+ of Polar Bear and Paleo-Eskimo
48.1	Difference in the verage length of endogenous DNA inserts in MBD- of Polar Bear and Paleo-Eskimo

TableS4.2_p-values lmm

		p-value		
Specie	Sample	read length	%GC	CpG density
Mammuthus primigenius	MKb2_1	2.5E-05	6.0E-06	4.2E-06
	MKb2_2	2.8E-04	1.7E-05	9.8E-06
	MKb1_1	1.4E-06	3.0E-06	1.4E-06
	MKb1_2	3.3E-05	3.9E-06	2.9E-06
	MKhs_1	4.2E-05	3.2E-05	1.9E-05
	MKhs_2	1.0E-04	2.1E-05	2.6E-06
	MKm_1	2.3E-05	2.8E-05	1.2E-05
	MKm_2	2.7E-04	1.5E-04	1.0E-04
	MKs_1	1.4E-04	2.1E-04	1.1E-04
	MZh_1	8.1E-01	2.1E-01	2.0E-01
	MZl_1	6.6E-05	2.0E-04	2.9E-04
	MZm_1	1.4E-03	4.4E-03	1.1E-03
Ursus maritimus	PB25	1.1E-06	5.2E-07	5.2E-07
	PB44	2.5E-06	4.8E-07	3.5E-07
	PB9	1.1E-06	7.1E-07	6.3E-07
Equus caballus	EQ26	2.9E-06	2.3E-06	1.1E-06
	EQ34	5.6E-06	1.4E-05	5.7E-06
	EQ36	4.0E-06	1.5E-05	6.7E-06
Equus Lambei	EQA	4.0E-03	4.0E-05	1.0E-05
Homo sapiens	Saqqaq	7.3E-06	5.7E-06	1.1E-04

TableS4.3_DeltaS DeltaD Overhan

Sample	Library	Genome	minDeltaD	lowDeltaD	midDeltaD	midDeltaD MBD- / midDeltaD MBD+	topDeltaD	maxDeltaD	minDeltaS	lowDeltaS	midDeltaS	midDeltaS MBD- / midDeltaS MBD+	topDeltaS	maxDeltaS	minOverhang	lowOverhang	midOverhang	topOverhang	maxOverhang
EQ26	MBD+	Nuclear	0.031107	0.033763	0.034808	1.548	0.035811	0.038177	0.295088	0.321148	0.332993	1.124	0.345306	0.379554	1.409888286	1.681884103	1.80329599	1.930228668	2.278626085
EQ26	MBD-	Nuclear	0.051994	0.053358	0.053884		0.054404	0.055704	0.349815	0.367207	0.374329		0.382017	0.402014	1.359542087	1.505901828	1.563862561	1.623389287	1.79486835
EQ34	MBD+	Nuclear	0.000256	0.001785	0.002515	1.893	0.003233	0.005082	0.145641	0.189597	0.21244	1.177	0.238747	0.323062	0.671424708	0.968415396	1.116486998	1.283827943	1.77889432
EQ34	MBD-	Nuclear	0.003995	0.00454	0.004762		0.004982	0.005517	0.226091	0.242586	0.250117		0.257433	0.277969	0.974878929	1.068625492	1.108097813	1.151107078	1.258808457
EQ36	MBD+	Nuclear	0.015547	0.019753	0.021304	1.043	0.022746	0.026087	0.19177	0.244646	0.271088	1.074	0.302389	0.399771	0.739305656	1.114665126	1.309183226	1.545127114	2.347169071
EQ36	MBD-	Nuclear	0.020402	0.021697	0.022226		0.022744	0.023969	0.264793	0.283258	0.291238		0.29948	0.321236	1.385514297	1.549468502	1.621468589	1.695728773	1.896958809
EQA	MBD+	Nuclear	0.013556	0.022455	0.025253	1.618	0.02775	0.03311	0.224094	0.288155	0.321831	1.397	0.361445	0.505453	0.751713142	1.289661125	1.567506436	1.933336621	3.28251488
EQA	MBD-	Nuclear	0.039598	0.040494	0.040849		0.041209	0.04203	0.432438	0.444436	0.449696		0.455042	0.468858	1.51648757	1.59825286	1.632283531	1.666661956	1.751456753
HSSR	MBD+	Nuclear	0.004346	0.004397	0.004418	0.973	0.004439	0.00449	0.194761	0.197425	0.198506	0.921	0.199594	0.202387	0.730018857	0.742449661	0.74737441	0.752536251	0.76478047
HSSR	MBD-	Nuclear	0.004261	0.004285	0.004296		0.004307	0.004334	0.181412	0.182433	0.182863		0.183306	0.184316	0.959961053	0.966382014	0.969359944	0.972185027	0.978755844
MKb1_1	MBD+	Nuclear	0.002146	0.002647	0.002856	3.377	0.003069	0.003539	0.187185	0.190832	0.192392	1.057	0.193926	0.197718	2.167302091	2.251564416	2.287919332	2.325417712	2.416922389
MKb1_1	MBD-	Nuclear	0.009538	0.009616	0.009645		0.009677	0.009755	0.201702	0.202911	0.2034		0.203884	0.205123	1.490294943	1.504953667	1.510932791	1.516681297	1.531312985
MKb1_2	MBD+	Nuclear	0.004953	0.006307	0.006815	1.863	0.007317	0.008373	0.097168	0.102269	0.104417	1.605	0.106746	0.112665	2.397718178	2.734105456	2.897154869	3.063948598	3.532325111
MKb1_2	MBD-	Nuclear	0.012425	0.012618	0.012694		0.012769	0.012954	0.163907	0.166468	0.167561		0.168581	0.171073	1.554464043	1.596108094	1.61365938	1.631484554	1.677421683
MKb2_1	MBD+	Nuclear	0.007333	0.00812	0.008436	1.536	0.008735	0.009472	0.257826	0.274347	0.28101	1.040	0.2878	0.30665	1.065913722	1.171521161	1.214131783	1.258968975	1.382579301
MKb2_1	MBD-	Nuclear	0.012831	0.01292	0.012957		0.012991	0.013079	0.289437	0.291362	0.292206		0.293072	0.29514	1.183240812	1.196306342	1.201619808	1.207025265	1.219707941
MKb2_2	MBD+	Nuclear	0.00783	0.009201	0.009705	1.379	0.010188	0.011306	0.143487	0.158925	0.165928	1.225	0.173295	0.193841	1.180390291	1.420126093	1.533185726	1.653758063	1.992800116
MKb2_2	MBD-	Nuclear	0.0131	0.013299	0.013378		0.013458	0.013667	0.199112	0.202048	0.203263		0.204501	0.207647	1.494308245	1.534656773	1.550994214	1.567288326	1.606673251
MKhs_1	MBD+	Nuclear	0.000713	0.002199	0.002779	2.401	0.003368	0.004754	0.15133	0.168813	0.176758	1.056	0.184836	0.207961	1.249471627	1.511815522	1.629795788	1.75797452	2.110075843
MKhs_1	MBD-	Nuclear	0.006474	0.006613	0.006671		0.006726	0.00687	0.184363	0.185941	0.186593		0.187255	0.188923	1.990612663	2.020864819	2.033004089	2.045178154	2.075160351
MKhs_2	MBD+	Nuclear	0.001984	0.005094	0.006156	1.476	0.007191	0.009482	0.074565	0.095428	0.106449	1.437	0.119288	0.162012	0.878229418	1.413884383	1.711674904	2.077454747	3.405837913
MKhs_2	MBD-	Nuclear	0.008546	0.008934	0.009087		0.009241	0.009609	0.14833	0.151677	0.153008		0.154343	0.1577	2.114241606	2.205203779	2.242301229	2.280021523	2.383661447
MKm_1	MBD+	Nuclear	0.003417	0.005578	0.00641	1.414	0.00721	0.009192	0.184056	0.224269	0.243897	0.788	0.266579	0.336584	0.67719737	0.942719581	1.071570591	1.219117052	1.662539811
MKm_1	MBD-	Nuclear	0.007863	0.008728	0.009061		0.009397	0.010191	0.171186	0.185674	0.192254		0.199149	0.21811	1.100576668	1.266203249	1.336717737	1.41362124	1.609777917
MKm_2	MBD+	Nuclear	0.006967	0.009238	0.01013	0.880	0.010994	0.013127	0.125505	0.160748	0.181244	0.890	0.206188	0.297824	0.495265671	0.832563163	1.006043181	1.210057781	1.773620455
MKm_2	MBD-	Nuclear	0.006096	0.008171	0.008913		0.009667	0.011357	0.127114	0.150378	0.161266		0.172947	0.206356	1.108070884	1.443151332	1.605656773	1.791118414	2.391839067
MKs_1	MBD+	Nuclear	0.006971	0.010019	0.011116	0.983	0.012128	0.014586	0.156619	0.17995	0.190361	1.072	0.201867	0.235476	1.34085507	1.743913481	1.939657741	2.151047879	2.838034371
MKs_1	MBD-	Nuclear	0.009884	0.010617	0.010923		0.011218	0.011937	0.190801	0.200086	0.204082		0.208203	0.218699	1.55750438	1.687161556	1.743118908	1.802296222	1.952956478
MZh_1	MBD+	Nuclear	0.000296	0.003633	0.005681	1.556	0.007653	0.012041	0.093273	0.11833	0.131459	1.423	0.146748	0.202444	1.013176799	1.900239015	2.367708091	2.932742543	4.404519825
MZh_1	MBD-	Nuclear	0.001814	0.007054	0.008838		0.010514	0.014419	0.11069	0.156394	0.187076		0.224739	0.384605	0.402939219	0.886912161	1.185605947	1.593328868	3.084387453
MZl_1	MBD+	Nuclear	0.003012	0.007108	0.008501	1.719	0.009819	0.012663	0.138296	0.15805	0.167332	1.751	0.177715	0.207898	1.546417657	2.074790749	2.339700885	2.646586973	3.589829061
MZl_1	MBD-	Nuclear	0.013654	0.014329	0.014609		0.014887	0.015564	0.280164	0.289078	0.29293		0.296859	0.306782	1.558713177	1.642467465	1.679730006	1.716421954	1.809332091
MZm_1	MBD+	Nuclear	0.00425	0.006552	0.007358	0.894	0.008116	0.009907	0.147493	0.168361	0.177917	1.185	0.187949	0.21568	1.304316548	1.643372398	1.803349852	1.984155598	2.555519428
MZm_1	MBD-	Nuclear	0.00302	0.005584	0.006579		0.007493	0.009635	0.171575	0.197524	0.210852		0.225971	0.272117	1.041786011	1.404613075	1.576094988	1.770096308	2.310095918
PB25	MBD+	Nuclear	0.001127	0.001213	0.001249	1.862	0.001284	0.00137	0.038317	0.040408	0.041345	0.937	0.042275	0.044783	0.888648723	0.962726934	0.993153667	1.024504141	1.105907713
PB25	MBD-	Nuclear	0.002173	0.002281	0.002326		0.002369	0.002469	0.036373	0.03802	0.038727		0.039478	0.041356	1.33517448	1.436037041	1.478849016	1.524740071	1.640955031
PB44	MBD+	Nuclear	0.000651	0.000846	0.000923	5.738	0.001002	0.001189	0.255458	0.261165	0.263656	1.106	0.266064	0.272096	1.053613393	1.083598461	1.095781154	1.108552265	1.139954991
PB44	MBD-	Nuclear	0.005129	0.005243	0.005294		0.005342	0.005456	0.283705	0.289151	0.291501		0.293776	0.299513	0.912835452	0.933094001	0.941680724	0.950363832	0.971165545
PB9	MBD+	Nuclear	0.002209	0.002354	0.002409	2.019	0.002466	0.002602	0.081474	0.083696	0.084689	1.242	0.085644	0.088144	1.436638889	1.503781363	1.529755961	1.556082532	1.624289959
PB9	MBD-	Nuclear	0.004647	0.004802	0.004865		0.004928	0.005078	0.101377	0.104013	0.105176		0.106349	0.109146	1.380228844	1.434871617	1.459876745	1.484918732	1.547565698
					AVERAGE	1.808					AVERAGE	1.175							
					MIN	0.880					MIN	0.788							
					MAX	5.738					MAX	1.751							

TableS4.4_Khroma DeltaS and Del

ID	Fraction	midDeltaD
MKb1_1	MBD+	0.003
MKb1_1	MBD-	0.010
MKb1_2	MBD+	0.007
MKb1_2	MBD-	0.013
MKb2_1	MBD+	0.008
MKb2_1	MBD-	0.013
MKb2_2	MBD+	0.010
MKb2_2	MBD-	0.013
MKhs_1	MBD+	0.003
MKhs_1	MBD-	0.007
MKhs_2	MBD+	0.007
MKhs_2	MBD-	0.009
MKm_1	MBD+	0.010
MKm_1	MBD-	0.009
MKm_2	MBD+	0.011
MKm_2	MBD-	0.011
MKs_1	MBD+	0.017
MKs_1	MBD-	0.014

KHROMA	Fraction	Average midDeltaD	midDeltaD Other tissue/ Bone
Muscle	MBD+	0.011	1.53
Skin	MBD+	0.017	2.43
Hair skin	MBD+	0.005	0.68
Bone	MBD+	0.007	
Muscle	MBD-	0.010	0.83
Skin	MBD-	0.014	1.12
Hair skin	MBD-	0.008	0.64
Bone	MBD-	0.012	
		min	0.64
		max	2.43
		average	1.20

ID	Fraction	midDeltaS
MKb1_1	MBD+	0.192
MKb1_1	MBD-	0.203
MKb1_2	MBD+	0.104
MKb1_2	MBD-	0.167
MKb2_1	MBD+	0.282
MKb2_1	MBD-	0.292
MKb2_2	MBD+	0.165
MKb2_2	MBD-	0.204
MKhs_1	MBD+	0.182
MKhs_1	MBD-	0.187
MKhs_2	MBD+	0.246
MKhs_2	MBD-	0.192
MKm_1	MBD+	0.184
MKm_1	MBD-	0.162
MKm_2	MBD+	0.192
MKm_2	MBD-	0.204
MKs_1	MBD+	0.201
MKs_1	MBD-	0.232

KHROMA	Fraction	Average midDeltaS	midDeltaS Other tissue/ Bone
Muscle	MBD+	0.188	1.01
Skin	MBD+	0.201	1.08
Hair skin	MBD+	0.214	1.15
Bone	MBD+	0.186	
Muscle	MBD-	0.183	0.84
Skin	MBD-	0.232	1.07
Hair skin	MBD-	0.189	0.87
Bone	MBD-	0.217	
		**min**	0.84
		**max**	1.15
		**average**	1.01

ID	Fraction	midDeltaS
MKb1_1	MBD+	0.192392
MKb1_1	MBD-	0.2034
MKb1_2	MBD+	0.104417
MKb1_2	MBD-	0.167561
MKb2_1	MBD+	0.28101
MKb2_1	MBD-	0.292206
MKb2_2	MBD+	0.165928
MKb2_2	MBD-	0.203263

KHROMA	Fraction	Average midDeltaS	midDeltaS bone 2 / bone 1	
MKb1	MBD+	0.148		
MKb2	MBD+	0.223	1.51	
MKb1	MBD-	0.185		
MKb2	MBD-	0.248	1.34	
		0.15	1.34	**min**
		0.25	1.51	**max**
		0.20	1.42	**average**

ID	Fraction	midDeltaD
MKb1_1	MBD+	0.002856
MKb1_1	MBD-	0.009645
MKb1_2	MBD+	0.006815
MKb1_2	MBD-	0.012694
MKb2_1	MBD+	0.008436
MKb2_1	MBD-	0.012957
MKb2_2	MBD+	0.009705
MKb2_2	MBD-	0.013378

KHROMA	Fraction	Average midDeltaD	
MKb1	MBD+	0.005	
MKb2	MBD+	0.009	
MKb1	MBD-	0.011	
MKb2	MBD-	0.013	
		0.005	**min**
		0.013	**max**
		0.010	**average**

ID	Fraction	midDeltaD
PB25	MBD+	0.001249
PB25	MBD-	0.002326
PB44	MBD+	0.000923
PB44	MBD-	0.005294
PB9	MBD+	0.002409
PB9	MBD-	0.004865

PB	Fraction	Average midDeltaD	
PB	MBD+	0.002	
PB	MBD-	0.004	
		0.002	**min**
		0.004	**max**
		0.003	**average**

TableS4.5_p-values

Specie	p-values DeltaD	p-values DeltaS	p-values Lambda
Ursus maritimus	8.6E-02	8.9E-01	7.4E-01
Equus caballus	6.9E-01	5.5E-01	9.4E-01
Mammuthus primigenius	4.0E-02	1.3E-01	3.3E-01

TableS5.1_dimers relative amoun

		Dimers relative abundancy																Relative abundancy of CG over:															
Sample	Library	AA	AC	AG	AT	CA	CC	**CG**	CT	GA	GC	GG	GT	TA	TC	TG	TT	AA	AC	AG	AT	CA	CC	**CG**	CT	GA	GC	GG	GT	TA	TC	TG	TT
EQ26	MBD+	0.035	0.057	0.071	0.034	0.078	0.082	**0.071**	0.071	0.061	0.102	0.083	0.058	0.023	0.062	0.079	0.036	1.997	1.250	0.995	2.098	0.905	0.866	**1.000**	0.996	1.153	0.695	0.854	1.217	3.120	1.147	0.893	1.979
EQ26	MBD-	0.075	0.055	0.077	0.080	0.078	0.044	**0.013**	0.077	0.067	0.047	0.044	0.055	0.068	0.067	0.078	0.075	0.175	0.241	0.172	0.164	0.169	0.298	**1.000**	0.172	0.197	0.282	0.297	0.240	0.195	0.198	0.168	0.175
EQ34	MBD+	0.037	0.055	0.073	0.036	0.077	0.077	**0.069**	0.070	0.063	0.097	0.081	0.060	0.024	0.063	0.079	0.039	1.841	1.249	0.944	1.904	0.892	0.891	**1.000**	0.976	1.093	0.705	0.841	1.144	2.816	1.085	0.867	1.743
EQ34	MBD-	0.067	0.055	0.080	0.071	0.081	0.050	**0.016**	0.080	0.068	0.053	0.050	0.055	0.057	0.068	0.082	0.067	0.237	0.291	0.200	0.225	0.197	0.320	**1.000**	0.199	0.235	0.299	0.320	0.289	0.279	0.236	0.195	0.237
EQ36	MBD+	0.039	0.058	0.071	0.038	0.079	0.076	**0.066**	0.070	0.063	0.093	0.078	0.060	0.025	0.064	0.080	0.040	1.692	1.141	0.929	1.746	0.833	0.862	**1.000**	0.938	1.048	0.709	0.843	1.094	2.632	1.028	0.827	1.651
EQ36	MBD-	0.073	0.055	0.078	0.077	0.080	0.046	**0.015**	0.078	0.067	0.050	0.046	0.054	0.062	0.067	0.080	0.072	0.202	0.269	0.187	0.191	0.183	0.317	**1.000**	0.189	0.219	0.294	0.316	0.269	0.236	0.220	0.184	0.203
EQA	MBD+	0.047	0.055	0.073	0.046	0.075	0.069	**0.058**	0.078	0.063	0.088	0.070	0.053	0.036	0.068	0.073	0.049	1.233	1.064	0.801	1.267	0.779	0.844	**1.000**	0.748	0.925	0.658	0.836	1.101	1.602	0.853	0.797	1.193
EQA	MBD-	0.066	0.055	0.079	0.068	0.081	0.053	**0.018**	0.080	0.068	0.055	0.053	0.055	0.055	0.068	0.080	0.066	0.267	0.318	0.222	0.260	0.219	0.330	**1.000**	0.221	0.260	0.319	0.331	0.318	0.320	0.259	0.219	0.266
HSSR	MBD+	0.062	0.054	0.078	0.063	0.081	0.058	**0.024**	0.079	0.067	0.063	0.057	0.054	0.048	0.067	0.081	0.063	0.386	0.447	0.307	0.380	0.296	0.416	**1.000**	0.305	0.361	0.380	0.419	0.444	0.496	0.358	0.295	0.384
HSSR	MBD-	0.063	0.054	0.080	0.065	0.083	0.057	**0.019**	0.080	0.067	0.061	0.056	0.054	0.049	0.067	0.083	0.063	0.294	0.344	0.233	0.286	0.222	0.327	**1.000**	0.232	0.278	0.305	0.328	0.343	0.375	0.277	0.222	0.292
MKb1_1	MBD+	0.062	0.056	0.080	0.057	0.073	0.059	**0.049**	0.070	0.073	0.070	0.059	0.054	0.048	0.066	0.068	0.056	0.796	0.878	0.609	0.856	0.674	0.825	**1.000**	0.698	0.671	0.704	0.824	0.902	1.018	0.740	0.724	0.875
MKb1_1	MBD-	0.074	0.056	0.078	0.074	0.081	0.047	**0.013**	0.078	0.068	0.047	0.047	0.056	0.060	0.068	0.081	0.074	0.180	0.235	0.171	0.180	0.164	0.284	**1.000**	0.171	0.196	0.280	0.284	0.235	0.221	0.196	0.164	0.180
MKb1_2	MBD+	0.065	0.056	0.081	0.061	0.072	0.057	**0.046**	0.069	0.074	0.065	0.057	0.055	0.051	0.066	0.067	0.059	0.711	0.827	0.570	0.759	0.639	0.809	**1.000**	0.672	0.624	0.712	0.813	0.845	0.902	0.700	0.691	0.789
MKb1_2	MBD-	0.082	0.055	0.075	0.082	0.078	0.042	**0.011**	0.075	0.067	0.042	0.042	0.056	0.068	0.067	0.078	0.082	0.136	0.200	0.148	0.135	0.143	0.266	**1.000**	0.148	0.166	0.267	0.266	0.200	0.163	0.166	0.143	0.136
MKb2_1	MBD+	0.061	0.059	0.074	0.061	0.076	0.055	**0.042**	0.073	0.067	0.064	0.056	0.059	0.052	0.066	0.075	0.060	0.682	0.701	0.564	0.675	0.547	0.749	**1.000**	0.572	0.618	0.648	0.745	0.707	0.801	0.626	0.551	0.692
MKb2_1	MBD-	0.076	0.056	0.077	0.078	0.079	0.045	**0.012**	0.077	0.066	0.045	0.045	0.056	0.066	0.066	0.080	0.076	0.155	0.210	0.154	0.152	0.149	0.261	**1.000**	0.154	0.178	0.263	0.261	0.210	0.180	0.178	0.148	0.155
MKb2_2	MBD+	0.064	0.060	0.073	0.067	0.076	0.051	**0.037**	0.072	0.068	0.057	0.051	0.060	0.056	0.067	0.076	0.065	0.568	0.611	0.499	0.549	0.483	0.717	**1.000**	0.505	0.542	0.637	0.713	0.608	0.648	0.544	0.484	0.567
MKb2_2	MBD-	0.080	0.056	0.076	0.081	0.078	0.043	**0.011**	0.075	0.066	0.042	0.043	0.056	0.069	0.066	0.078	0.080	0.137	0.194	0.144	0.133	0.139	0.254	**1.000**	0.144	0.164	0.257	0.254	0.193	0.158	0.164	0.139	0.136
MKhs_1	MBD+	0.057	0.058	0.073	0.055	0.074	0.061	**0.052**	0.072	0.066	0.075	0.063	0.056	0.046	0.064	0.073	0.056	0.917	0.902	0.712	0.951	0.705	0.850	**1.000**	0.725	0.782	0.689	0.821	0.931	1.136	0.809	0.715	0.924
MKhs_1	MBD-	0.067	0.057	0.079	0.066	0.082	0.054	**0.016**	0.078	0.067	0.053	0.054	0.057	0.054	0.067	0.082	0.067	0.240	0.282	0.205	0.243	0.197	0.299	**1.000**	0.205	0.239	0.302	0.298	0.281	0.295	0.239	0.197	0.240
MKhs_2	MBD+	0.063	0.057	0.074	0.061	0.074	0.057	**0.043**	0.072	0.067	0.067	0.058	0.056	0.052	0.066	0.072	0.061	0.683	0.759	0.584	0.704	0.587	0.763	**1.000**	0.599	0.648	0.649	0.748	0.766	0.830	0.656	0.601	0.707
MKhs_2	MBD-	0.074	0.057	0.077	0.074	0.079	0.048	**0.013**	0.077	0.067	0.047	0.048	0.057	0.061	0.067	0.079	0.074	0.177	0.231	0.171	0.177	0.165	0.272	**1.000**	0.171	0.196	0.281	0.271	0.230	0.215	0.196	0.165	0.176
MKm_1	MBD+	0.061	0.056	0.075	0.060	0.075	0.056	**0.043**	0.075	0.067	0.070	0.056	0.055	0.049	0.066	0.074	0.062	0.698	0.773	0.576	0.714	0.574	0.763	**1.000**	0.574	0.644	0.611	0.760	0.784	0.873	0.647	0.577	0.694
MKm_1	MBD-	0.077	0.054	0.078	0.074	0.079	0.045	**0.014**	0.078	0.069	0.047	0.045	0.055	0.058	0.068	0.080	0.078	0.177	0.251	0.175	0.185	0.172	0.302	**1.000**	0.176	0.199	0.288	0.304	0.249	0.234	0.200	0.172	0.175
MKm_2	MBD+	0.070	0.055	0.076	0.069	0.075	0.049	**0.029**	0.075	0.068	0.056	0.050	0.057	0.057	0.067	0.076	0.071	0.410	0.522	0.378	0.419	0.382	0.588	**1.000**	0.385	0.425	0.511	0.577	0.504	0.506	0.426	0.378	0.406
MKm_2	MBD-	0.085	0.054	0.075	0.082	0.077	0.040	**0.011**	0.075	0.069	0.042	0.040	0.054	0.065	0.068	0.077	0.085	0.132	0.210	0.150	0.138	0.147	0.279	**1.000**	0.151	0.164	0.271	0.279	0.210	0.173	0.167	0.146	0.133
MKs_1	MBD+	0.068	0.057	0.074	0.067	0.076	0.051	**0.034**	0.074	0.067	0.060	0.051	0.057	0.055	0.066	0.076	0.067	0.498	0.590	0.454	0.506	0.446	0.662	**1.000**	0.457	0.502	0.564	0.656	0.592	0.610	0.512	0.443	0.503
MKs_1	MBD-	0.074	0.056	0.077	0.073	0.081	0.047	**0.015**	0.077	0.067	0.049	0.047	0.056	0.058	0.067	0.080	0.075	0.206	0.275	0.199	0.210	0.191	0.326	**1.000**	0.199	0.228	0.311	0.327	0.276	0.264	0.228	0.192	0.206
MZh_1	MBD+	0.070	0.058	0.079	0.068	0.081	0.050	**0.018**	0.077	0.067	0.052	0.050	0.058	0.057	0.066	0.081	0.069	0.259	0.317	0.232	0.268	0.227	0.366	**1.000**	0.236	0.272	0.351	0.368	0.315	0.319	0.276	0.227	0.263
MZh_1	MBD-	0.072	0.056	0.077	0.073	0.080	0.050	**0.019**	0.075	0.065	0.052	0.049	0.055	0.061	0.066	0.077	0.072	0.269	0.347	0.253	0.267	0.244	0.391	**1.000**	0.258	0.300	0.371	0.396	0.354	0.319	0.295	0.253	0.268
MZl_1	MBD+	0.073	0.057	0.075	0.074	0.080	0.049	**0.017**	0.077	0.065	0.049	0.047	0.055	0.063	0.067	0.077	0.073	0.236	0.303	0.231	0.235	0.218	0.357	**1.000**	0.226	0.269	0.353	0.367	0.314	0.276	0.258	0.226	0.237
MZl_1	MBD-	0.076	0.060	0.074	0.080	0.080	0.046	**0.014**	0.078	0.063	0.044	0.043	0.054	0.072	0.067	0.074	0.075	0.179	0.226	0.184	0.169	0.170	0.293	**1.000**	0.175	0.215	0.309	0.316	0.251	0.189	0.203	0.183	0.182
MZm_1	MBD+	0.068	0.056	0.078	0.068	0.080	0.052	**0.017**	0.078	0.067	0.053	0.053	0.057	0.056	0.068	0.081	0.069	0.254	0.311	0.222	0.257	0.216	0.332	**1.000**	0.224	0.260	0.330	0.330	0.302	0.313	0.257	0.216	0.252
MZm_1	MBD-	0.069	0.059	0.076	0.068	0.082	0.052	**0.021**	0.077	0.066	0.055	0.051	0.056	0.056	0.066	0.080	0.067	0.299	0.350	0.269	0.302	0.251	0.396	**1.000**	0.265	0.312	0.373	0.406	0.366	0.366	0.310	0.257	0.307
PB25	MBD+	0.047	0.056	0.082	0.046	0.080	0.070	**0.039**	0.081	0.068	0.076	0.070	0.056	0.035	0.068	0.080	0.047	0.840	0.704	0.483	0.862	0.492	0.564	**1.000**	0.483	0.574	0.515	0.563	0.707	1.136	0.575	0.494	0.844
PB25	MBD-	0.076	0.055	0.077	0.079	0.077	0.047	**0.012**	0.077	0.067	0.045	0.047	0.054	0.066	0.067	0.077	0.075	0.163	0.225	0.159	0.155	0.159	0.263	**1.000**	0.159	0.182	0.273	0.263	0.226	0.185	0.182	0.160	0.163
PB44	MBD+	0.035	0.052	0.079	0.031	0.077	0.086	**0.063**	0.078	0.063	0.103	0.087	0.052	0.022	0.062	0.077	0.034	1.806	1.199	0.789	2.039	0.811	0.729	**1.000**	0.798	0.988	0.608	0.722	1.212	2.870	1.002	0.817	1.856
PB44	MBD-	0.061	0.055	0.083	0.062	0.082	0.058	**0.018**	0.083	0.069	0.057	0.058	0.055	0.049	0.069	0.081	0.061	0.297	0.331	0.220	0.295	0.223	0.316	**1.000**	0.220	0.264	0.317	0.316	0.333	0.370	0.264	0.224	0.298
PB9	MBD+	0.052	0.056	0.080	0.052	0.079	0.065	**0.037**	0.080	0.069	0.071	0.065	0.056	0.041	0.068	0.079	0.052	0.708	0.657	0.462	0.710	0.467	0.570	**1.000**	0.462	0.539	0.520	0.570	0.662	0.908	0.540	0.469	0.713
PB9	MBD-	0.079	0.054	0.076	0.082	0.077	0.045	**0.012**	0.076	0.067	0.043	0.045	0.054	0.069	0.067	0.076	0.078	0.153	0.221	0.158	0.146	0.157	0.269	**1.000**	0.158	0.179	0.279	0.270	0.222	0.174	0.180	0.158	0.154
																	**max**							3.120									

	p-values		
Dimer	*Equus caballus*	*Mammuthus primigenius*	*Ursus maritimus*
ApA	1.4E-03	3.8E-05	2.1E-02
ApC	2.0E-01	3.0E-01	9.7E-01
ApG	1.0E-01	3.4E-01	5.2E-01
ApT	1.3E-03	3.6E-05	2.4E-02
CpA	1.9E-01	5.3E-04	9.0E-01
CpC	1.7E-04	3.1E-04	4.4E-02
CpG	3.0E-05	1.6E-06	5.2E-02
CpT	1.3E-02	2.6E-03	6.3E-01
GpA	2.6E-03	3.6E-01	1.0E+00
GpC	1.7E-04	4.2E-05	5.3E-02
GpG	1.1E-04	2.2E-04	4.6E-02
GpT	1.7E-02	5.2E-02	7.0E-01
TpA	4.2E-03	4.0E-04	2.6E-02
TpC	2.0E-02	1.4E-01	1.0E+00
TpG	1.0E+00	5.2E-03	9.5E-01
TpT	9.3E-04	6.8E-05	2.2E-02

TableS6 MetaPhlAn

[illegible]

TableS7 Microbial profiles

Equid samples[a]	trimmed reads	mapped reads[a]	genera	Shannon diversity[c]
EQA_MBD+	8894455	7303	5	1.43
EQA_MBD-	5776769	5683	9	1.72
EQB_MBD+	10136112	13338	6	1.46
EQB_MBD-	7000618	29875	10	1.47
EQC_MBD+	10509471	9397	7	1.59
EQC_MBD-	7065549	31402	15	1.56
EQE_MBD+	8435891	7598	10	1.91
EQE_MBD-	9195063	30192	12	1.49
EQF_MBD+	10060504	5788	8	1.74
EQF_MBD-	7550930	4076	12	2.03
**Kroma mammoth samples[a]**	**trimmed reads**	**mapped reads[b]**	**genera**	**Shannon diversity[c]**
MKb1_1_MBD+	10463207	3861	4	0.17
MKb1_1_MBD-	12872625	35883	6	0.05
MKb1_2_MBD+	2770052	4639	1	0
MKb1_2_MBD-	2385434	389	1	0
MKb2_1_MBD+	11880340	9435	6	0.89
MKb2_1_MBD-	11635626	13941	10	0.21
MKb2_2_MBD+	2989196	2883	3	0.09
MKb2_2_MBD-	2800110	733	2	0.51
MKhs_1_MBD+	11628153	8823	7	1.5
MKhs_1_MBD-	12680425	11795	16	1.33
MKhs_2_MBD+	2834480	2403	9	1.06
MKhs_2_MBD-	5239477	498	2	0.58
MKm_1_MBD+	11971341	14910	4	0.58
MKm_1_MBD-	16178263	36508	6	0.75
MKm_2_MBD+	3069468	7568	4	0.78
MKm_2_MBD-	15533439	22737	8	0.76
MKs_1_MBD+	12949713	17314	9	0.33
MKs_1_MBD-	20004888	35332	5	0.06
MKs_2_MBD+	2715457	4574	4	0.26
MKs_2_MBD-	2890744	2021	4	0.24
**Zhenya mammoth samples[a]**	**trimmed reads**	**mapped reads[b]**	**genera**	**Shannon diversity[c]**
MZh_1_MBD+	7171605	5557	9	1.47
MZh_1_MBD-	8067779	18631	9	1.08
MZh_2_MBD+	903182	2495	4	0.99
MZh_2_MBD-	2035979	339	2	0.68
MZl_1_MBD+	8702102	8180	9	1.26
MZl_1_MBD-	9004754	11227	16	1.81
MZl_2_MBD+	1901793	2654	11	1.65
MZl_2_MBD-	2516242	367	2	0.43
MZm_1_MBD+	10819017	8508	9	1.04
MZm_1_MBD-	13728406	21621	21	1.41
MZm_2_MBD+	3103006	6199	10	1.31
MZm_2_MBD-	2135223	555	2	0.69
**Polar bear samples[a]**	**trimmed reads**	**mapped reads[b]**	**genera**	**Shannon diversity[c]**
PB25_MBD+	2107980	1138	4	1.27
PB25_MBD-	1569088	1039	4	0.52
PB9_MBD+	1859912	1767	7	1.13
PB9_MBD-	1538441	2895	6	1.41

[a] "_MBD+" refers to the captured fraction, "_MBD-" refers to the uncaptured fraction; [b] to the MetaPhlAn database; [c] at the genus level.

Supplementary Table S6. MetaPhlAn microbial DNA profiles.

**Supplementary Table S7.** Ch		Number of non-equid mapped reads															
Accession r	Genome de	EQA_MBD+	EQA_MBD-	EQB_MBD+	EQB_MBD-	EQC_MBD+	EQC_MBD-	EQE_MBD+	EQE_MBD-	EQF_MBD+	EQF_MBD-	EQ26_MBD	EQ26_MBD	EQ34_MBD	EQ34_MBD	EQ36_MBD	EQ36_MBD-
Supplementar	Burkholderi	1724	1518	1508	3044	2398	5276	1950	4784	2820	5226	2944	108	1516	770	2736	568
CP001025.	Burkholderi	1732	1486	1488	2974	2312	5148	1936	4824	2678	5312	2860	96	1440	754	2530	540
CP000010.	Burkholderi	1308	1540	1174	2922	1806	5876	1590	5208	2028	5686	1912	88	1052	758	1610	652
CP000548.	Burkholderi	1286	1558	1158	2916	1796	5832	1582	5206	2034	5706	1906	86	1052	750	1618	648
CP000546.	Burkholderi	1282	1552	1152	2924	1800	5840	1570	5220	2020	5656	1902	86	1048	746	1614	642
BX571965.	Burkholderi	1308	1614	1110	2864	1682	5804	1470	5098	1912	5674	1864	68	1022	726	1602	606
CP000570.	Burkholderi	1274	1578	1080	2814	1626	5812	1432	5072	1904	5622	1866	74	1018	724	1600	594
CP000152.	Burkholderi	666	964	668	1696	1188	4364	964	3416	1350	3104	916	70	542	482	892	488
CP000441.	Burkholderi	612	824	652	1596	1158	3972	944	3104	1252	2800	908	66	520	468	848	494
CP000459.	Burkholderi	662	906	636	1684	1236	4296	992	3416	1342	2998	1008	56	536	452	980	500
CP003515.	Burkholderi	634	854	622	1492	1114	3914	894	3168	1196	2646	834	60	464	460	776	484
CP000869.	Burkholderi	610	880	610	1456	1160	3892	988	2856	1248	2644	914	58	514	418	890	444
CP000270.	Burkholderi	660	962	570	2058	846	4124	776	3722	1028	3430	634	40	350	586	622	458
CP002519.	Burkholderi	620	928	560	1870	776	3884	756	3428	942	3536	672	42	342	538	658	430
CP000573.	Burkholderi	586	872	538	1318	1044	4190	836	2952	1158	3072	716	64	396	436	644	504
CP002834.	Burkholderi	598	886	536	1310	1044	4206	818	2946	1156	3074	706	64	374	436	632	506
CP000125.	Burkholderi	596	894	534	1344	1060	4220	860	2960	1164	3072	722	64	392	436	654	504
CP000085.	Burkholderi	574	836	526	1272	1046	4022	840	2894	1126	2898	654	50	396	406	622	450
CP001043.	Burkholderi	566	882	520	1702	736	3444	624	3222	870	3232	656	36	378	482	622	402
CP000380.	Burkholderi	616	550	506	1112	990	2758	854	2266	1188	2278	802	62	454	362	734	412
CP003087.	Burkholderi	538	924	492	1972	754	4080	608	3634	888	3762	560	46	338	512	548	384
AP009387.	Burkholderi	386	360	410	752	770	2298	610	1580	852	1452	420	52	296	274	414	346
CP003775.	Burkholderi	384	736	388	1090	628	2520	496	2062	668	2060	670	24	276	254	580	220
CP000525.	Burkholderi	368	540	374	822	704	2252	536	1840	768	1886	484	30	258	264	444	288
CP000616.	Burkholderi	332	396	340	744	660	2464	582	1730	758	1586	312	54	238	314	340	358
CP003088.	Burkholderi	356	514	338	958	668	2892	564	2122	738	2008	314	44	220	344	334	364
CP000960.	Burkholderi	314	442	320	706	668	2322	574	1668	772	1570	386	46	234	302	376	336
AM747722	Burkholderi	308	352	306	678	650	2130	552	1600	724	1356	346	46	248	274	384	342
CP002600.	Burkholderi	298	946	298	1092	460	3300	322	2376	494	2678	238	20	156	330	178	266
CP003089.	Burkholderi	292	394	294	788	596	2402	524	1862	672	1496	252	46	196	266	282	320
CP000545.	Burkholderi	294	566	282	666	418	2062	312	1450	480	1768	378	14	178	190	322	184
CP001504.	Burkholderi	320	750	282	858	428	2704	316	1942	434	2180	310	22	170	260	264	252
FR687359.	Burkholderi	252	502	242	848	306	1830	296	1608	384	1882	228	12	140	280	204	240
CP001053.	Burkholderi	208	436	198	712	216	1760	188	1422	240	1316	146	14	62	222	88	136
CP002218.	Burkholderi	146	338	122	554	146	1360	142	1058	200	1082	76	12	56	136	70	122
CP002520.	Burkholderi	168	362	120	568	174	1504	130	1194	186	1072	108	12	40	138	78	120
CP002015.	Burkholderi	48	174	52	266	50	714	34	578	66	496	28	2	18	60	16	52

In pink read counts for the genomes selected for k-mer analyses of the captured fractions (genomes against which the largest number of reads were aligned in the captured fraction (in green)).
In blue, read counts for the genomes selected for k-mer analyses of the uncaptured fractions (genomes against which the largest number of reads were aligned in the uncaptured fraction (in green)).

"CA", captured fraction; "UC", uncaptured fraction.

TableS1 methods details

ID	Label	Material	Extraction Method	Capture Kit	Volume for capture (µl)	Purification prior to library building	Fraction	PCR cycles	sequencing
PB9	UM9_CGG_1_011578	bone <0.09 g	column	E2600	25 out of 200	minelute	MBD+	12	MiSeq 250 PE
							MBD-	12+10	MiSeq 250 PE
PB25	UM25_CGG_1_011594	bone <0.09 g	column	E2600	25 out of 200	minelute	MBD+	12	MiSeq 250 PE
							MBD-	12+10	MiSeq 250 PE
PB44	UM44_CGG_1_011613	bone <0.09 g	column	E2612	50 out of 200	EtOH precipitation	MBD+	12	MiSeq 250 PE
							MBD-	12+10	MiSeq 250 PE
EQ26	CGG_1_010026	bone, 2.31g	column	E2612	21.25 out of 150	minelute	MBD+	9+8	MiSeq 150 PE
							MBD-	12+11	MiSeq 150 PE
EQ34	CGG_1_010034	bone, 2.04g	column	E2612	21.25 out of 150	minelute	MBD+	9+9	MiSeq 150 PE
							MBD-	12+12	MiSeq 150 PE
EQ36	CGG_1_010036	bone, 1.74g	column	E2612	21.25 out of 150	minelute	MBD+	9+9	MiSeq 150 PE
							MBD-	12+12	MiSeq 150 PE
EQA	CGG_1_014640	bone, 0.174g	silica pellets	E2600	21.25 out of 150	minelute	MBD+	10+8	HiSeq 150 RPE
							MBD-	13+10	HiSeq 150 RPE
EQB	CGG_1_015509	tooth, 0.341g	silica pellets	E2600	21.25 out of 150	minelute	MBD+	10+8	HiSeq 150 RPE
							MBD-	13+10	HiSeq 150 RPE
EQC	CGG_1_015508	tooth, 0.372g	silica pellets	E2600	21.25 out of 150	minelute	MBD+	10+8	HiSeq 150 RPE
							MBD-	13+10	HiSeq 150 RPE
EQE	CGG_1_015510	tooth, 0.341g	silica pellets	E2600	21.25 out of 150	minelute	MBD+	10+8	HiSeq 150 RPE
							MBD-	13+10	HiSeq 150 RPE
EQF	CGG_1_014638	tooth, 0.144g	silica pellets	E2600	21.25 out of 150	minelute	MBD+	10+8	HiSeq 150 RPE
							MBD-	13+10	HiSeq 150 RPE
MZh	m1_heart_Zhenya	heart	silica pellets	E2600	21.25 out of 150	minelute	MBD+	12+8	HiSeq 150 RPE
							MBD-	14+11	HiSeq 150 RPE
							MBD+	8+6	Gallx
							MBD-	12+9	Gallx
MZl	m2_liver_Zhenya	liver	silica pellets	E2600	21.25 out of 150	minelute	MBD+	12+8	HiSeq 150 RPE
							MBD-	14+11	HiSeq 150 RPE
							MBD+	8+6	
							MBD-	12+9	
MZm	m3_muscle_Zhenya	muscle	silica pellets	E2600	21.25 out of 150	minelute	MBD+	12+8	HiSeq 150 RPE
							MBD-	14+11	HiSeq 150 RPE
							MBD+	8+6	Gallx
							MBD-	12+9	Gallx
MKm	m4_muscle_Kroma	muscle	silica pellets	E2600	21.25 out of 150	minelute	MBD+	12+8	HiSeq 150 RPE
							MBD-	14+11	HiSeq 150 RPE
							MBD+	8+6	Gallx
							MBD-	12+9	Gallx
MKs	m5_skin_Kroma	skin	silica pellets	E2600	21.25 out of 150	minelute	MBD+	12+8	HiSeq 150 RPE
							MBD-	14+11	HiSeq 150 RPE
							MBD+	8+6	Gallx
							MBD-	12+9	Gallx
MKhs	m6_hair_skin_Kroma	hair and skin	silica pellets	E2600	21.25 out of 150	minelute	MBD+	12+8	HiSeq 150 RPE
							MBD-	14+11	HiSeq 150 RPE
							MBD+	8+6	Gallx
							MBD-	12+9	Gallx
MKb2	m7_bone2_Kroma	bone	silica pellets	E2600	21.25 out of 150	minelute	MBD+	12+8	HiSeq 150 RPE
							MBD-	14+11	HiSeq 150 RPE
							MBD+	8+6	Gallx
							MBD-	12+9	Gallx
MKb1	m8_bone_pectoral_vertebra_Kroma	bone	silica pellets	E2600	21.25 out of 150	minelute	MBD+	12+8	HiSeq 150 RPE
							MBD-	14+11	HiSeq 150 RPE
							MBD+	8+6	Gallx
							MBD-	12+9	Gallx
HSSR	Saqqaq	hair, 230 mg	Phenol/chloroform + column	E2600	70 out of 80	minelute	MBD+	15+7	HiSeq 75RPE
							MBD-	10	HiSeq 75RPE
							MBD+	12+10	HiSeq 75RPE
							MBD-	8	HiSeq 75RPE

TableS2.1_endogenous content

Sample	Fraction	Endogenous content Q0 (%)	Endogenous content MBD- Q0/ Endogenous content MBD+ Q0	Endogenous content Q25 (%)	Endogenous content MBD- Q25/ Endogenous content MBD+ Q25
EQ26	MBD+	4.26	8.77	0.83	37.26
EQ26	MBD-	37.35		30.77	
EQ34	MBD+	1.60	4.06	0.28	17.14
EQ34	MBD-	6.49		4.72	
EQ36	MBD+	1.46	3.31	0.26	14.36
EQ36	MBD-	4.84		3.70	
EQA	MBD+	0.12	22.30	0.02	89.82
EQA	MBD-	2.72		2.22	
HSS	MBD+	1.91	38.19	0.94	57.01
HSS	MBD-	73.13		53.73	
HSSR	MBD+	18.49	4.91	7.08	8.51
HSSR	MBD-	90.84		60.25	
MKb1_1	MBD+	24.55	2.66	3.77	12.57
MKb1_1	MBD-	65.31		47.33	
MKb1_2	MBD+	19.89	3.30	5.03	10.08
MKb1_2	MBD-	65.72		50.73	
MKb2_1	MBD+	2.62	21.17	0.54	77.47
MKb2_1	MBD-	55.40		41.57	
MKb2_2	MBD+	2.47	22.98	1.27	34.42
MKb2_2	MBD-	56.76		43.69	
MKhs_1	MBD+	0.89	36.50	0.21	106.97
MKhs_1	MBD-	32.48		21.95	
MKhs_2	MBD+	0.32	81.82	0.14	137.56
MKhs_2	MBD-	26.00		18.83	
MKm_1	MBD+	0.17	2.38	0.07	4.38
MKm_1	MBD-	0.41		0.31	
MKm_2	MBD+	0.10	4.52	0.05	7.46
MKm_2	MBD-	0.46		0.35	
MKs_1	MBD+	0.20	3.39	0.08	5.87
MKs_1	MBD-	0.68		0.46	
MKs_2	MBD+	0.15	6.24	0.08	8.29
MKs_2	MBD-	0.91		0.66	
MZh_1	MBD+	0.10	0.66	0.05	0.84
MZh_1	MBD-	0.07		0.04	
MZl_1	MBD+	0.27	25.43	0.13	32.52
MZl_1	MBD-	6.95		4.22	
MZm_1	MBD+	0.23	0.58	0.13	0.65
MZm_1	MBD-	0.14		0.09	
PB25	MBD+	40.74	1.28	35.30	1.32
PB25	MBD-	52.09		46.69	
PB44	MBD+	54.29	0.72	23.32	1.28
PB44	MBD-	38.85		29.97	
PB9	MBD+	41.40	1.16	33.68	1.21
PB9	MBD-	48.05		40.69	
		minimum	0.58	minimum	0.65
		maximum	81.82	maximum	137.56
		average	13.47	average	30.32
		median	4.29	median	11.32

KHROMA	Fraction	Average Endogenous Content Q25	Endogenous contnent Bone / Other tissue
Muscle	MBD+	0.12	22.74
Skin	MBD+	0.08	33.59
Hair skin	MBD+	0.17	15.50
Bone	MBD+	2.65	
Muscle	MBD-	0.33	140.00
Skin	MBD-	0.46	98.84
Hair skin	MBD-	29.24	1.57
Bone	MBD-	45.83	
		**min**	1.57
		**max**	140.00
		**average**	52.04
		**median**	28.17

TableS2.2_clonality

Sample	*Fraction*	*Clonality Q0 (%)*	Clonality MBD+ Q0/ Clonality MBD- Q0	*Further Clonality Q25 (%)*	Clonality MBD+ Q25/ Clonality MBD- Q25
EQ26	MBD+	18.85	24.17	84.25	4.62
EQ26	MBD-	0.78		18.24	
EQ34	MBD+	1.92	6.00	83.09	3.03
EQ34	MBD-	0.32		27.43	
EQ36	MBD+	3.09	7.23	82.92	3.47
EQ36	MBD-	0.43		23.93	
EQA	MBD+	57.86	14.79	91.47	4.22
EQA	MBD-	3.91		21.65	
HSSR	MBD+	72.72	86.04	89.55	2.62
HSSR	MBD-	0.85		34.23	
MKb1_1	MBD+	30.61	51.88	89.35	3.20
MKb1_1	MBD-	0.59		27.96	
MKb1_2	MBD+	9.44	45.06	77.08	3.35
MKb1_2	MBD-	0.21		22.98	
MKb2_1	MBD+	67.93	40.37	93.43	3.56
MKb2_1	MBD-	1.68		26.22	
MKb2_2	MBD+	21.26	43.63	59.54	2.54
MKb2_2	MBD-	0.49		23.41	
MKhs_1	MBD+	59.71	67.12	90.71	2.75
MKhs_1	MBD-	0.89		33.03	
MKhs_2	MBD+	27.62	106.80	68.81	2.48
MKhs_2	MBD-	0.26		27.75	
MKm_1	MBD+	28.67	231.86	71.40	2.71
MKm_1	MBD-	0.12		26.39	
MKm_2	MBD+	33.44	391.40	69.14	2.92
MKm_2	MBD-	0.09		23.66	
MKs_1	MBD+	38.76	174.20	75.92	2.36
MKs_1	MBD-	0.22		32.11	
MKs_2	MBD+	10.01	103.64	51.01	1.84
MKs_2	MBD-	0.10		27.66	
MZh_1	MBD+	24.39	12.09	66.46	1.50
MZh_1	MBD-	2.02		44.35	
MZl_1	MBD+	64.27	6.59	83.03	1.84
MZl_1	MBD-	9.75		45.19	
MZm_1	MBD+	14.92	19.24	50.98	1.41
MZm_1	MBD-	0.78		36.08	
PB25	MBD+	31.84	41.93	40.94	3.70
PB25	MBD-	0.76		11.06	
PB44	MBD+	20.05	11.14	65.65	2.71
PB44	MBD-	1.80		24.25	
PB9	MBD+	22.61	37.87	37.03	2.34
PB9	MBD-	0.60		15.81	
		minimum	6.00	minimum	1.41
		maximum	391.40	maximum	4.62
		average	72.53	average	2.82
		median	41.93	median	2.71

TableS2.3_mt_proportion

			mtDNA		nuDNA		
Sample	Fraction	Total Reads	Uniquely mapped Q0	Uniquely mapped Q25	Uniquely mapped Q0	Uniquely mapped Q25	(N reads mtDNA MBD- / N reads nuDNA MBD-) / (N reads mtDNA MBD+ / N reads nuDNA MBD+)
HSSR	MBD+	43,779,216	12,381	12,380	8,081,030	3,086,948	0.91
HSSR	MBD-	19,393,362	42,430	42,427	17,574,431	11,642,589	
MKb1_1	MBD+	10,461,328	387	363	2,568,020	393,701	2.96
MKb1_1	MBD-	12,872,086	17,089	16,564	8,390,055	6,076,225	
MKb1_2	MBD+	2,385,434	117	116	474,281	119,978	3.10
MKb1_2	MBD-	2,770,052	4,257	4,201	1,816,283	1,400,949	
MKb2_1	MBD+	11,878,035	163	130	310,726	63,605	1.88
MKb2_1	MBD-	11,636,012	18,991	18,545	6,427,106	4,818,528	
MKb2_2	MBD+	2,800,110	54	52	69,117	35,486	2.75
MKb2_2	MBD-	2,989,196	5,316	5,239	1,691,452	1,300,723	
MKhs_1	MBD+	11,628,856	225	212	103,253	23,649	1.08
MKhs_1	MBD-	12,679,846	27,511	26,742	4,091,309	2,756,454	
MKhs_2	MBD+	5,239,477	96	94	16,555	7,080	1.08
MKhs_2	MBD-	2,836,386	7,618	7,519	729,900	526,695	
MKm_1	MBD+	11,966,724	64	58	20,756	8,290	2.79
MKm_1	MBD-	16,177,223	982	946	66,056	48,462	
MKm_2	MBD+	15,533,439	90	89	15,604	7,187	0.55
MKm_2	MBD-	3,069,468	76	73	13,956	10,648	
MKs_1	MBD+	12,949,142	338	316	25,664	9,907	2.82
MKs_1	MBD-	20,004,345	8,040	7,645	128,276	85,109	
MKs_2	MBD+	2,890,744	101	96	4,135	2,210	2.04
MKs_2	MBD-	2,717,680	1,507	1,462	23,327	16,521	
MZh_1	MBD+	7,171,605	50	48	7,478	3,291	23.61
MZh_1	MBD-	8,067,779	827	813	4,761	2,361	
MZl_1	MBD+	8,702,102	3,078	2,757	20,691	8,531	5.80
MZl_1	MBD-	9,004,754	254,683	247,730	370,837	132,143	
MZm_1	MBD+	10,819,017	237	230	24,963	14,288	15.62
MZm_1	MBD-	13,728,406	2,468	2,418	16,216	9,618	
PB25	MBD+	2,107,980	63	57	858,663	744,109	16.24
PB25	MBD-	1,569,088	920	910	816,483	731,666	
PB9	MBD+	1,859,912	197	194	769,747	626,263	8.82
PB9	MBD-	1,538,441	1,717	1,706	737,452	624,321	
						minimum	0.55
						maximum	23.61
						average	6.78
						median	2.82

TableS2.4_genome coverage

Sample	Library	nuDNA coverage (Q25)
EQ26	MBD+	0.0005970
EQ26	MBD-	0.0019836
EQ34	MBD+	0.0001018
EQ34	MBD-	0.0007857
EQ36	MBD+	0.0001022
EQ36	MBD-	0.0006359
EQA	MBD+	0.0000508
EQA	MBD-	0.0030969
EQB	MBD+	0.0000180
EQB	MBD-	0.0000235
EQC	MBD+	0.0000331
EQC	MBD-	0.0000470
EQE	MBD+	0.0000165
EQE	MBD-	0.0000206
EQF	MBD+	0.0000213
EQF	MBD-	0.0003628
HSSR	MBD+	0.0592719
HSSR	MBD-	0.2026680
MKb1_1	MBD+	0.0123614
MKb1_1	MBD-	0.1230580
MKb1_2	MBD+	0.0028261
MKb1_2	MBD-	0.0318464
MKb2_1	MBD+	0.0017618
MKb2_1	MBD-	0.1084837
MKb2_2	MBD+	0.0008387
MKb2_2	MBD-	0.0291845
MKhs_1	MBD+	0.0005857
MKhs_1	MBD-	0.0490238
MKhs_2	MBD+	0.0001402
MKhs_2	MBD-	0.0105957
MKm_1	MBD+	0.0002046
MKm_1	MBD-	0.0009363
MKm_2	MBD+	0.0001551
MKm_2	MBD-	0.0002212
MKs_1	MBD+	0.0001883
MKs_1	MBD-	0.0012954
MKs_2	MBD+	0.0000429
MKs_2	MBD-	0.0003066
MZh_1	MBD+	0.0000590
MZh_1	MBD-	0.0000438
MZh_2	MBD+	0.0000244
MZh_2	MBD-	0.0000049
MZl_1	MBD+	0.0001721
MZl_1	MBD-	0.0033100
MZl_2	MBD+	0.0000206
MZl_2	MBD-	0.0007074
MZm_1	MBD+	0.0002535
MZm_1	MBD-	0.0001851
MZm_2	MBD+	0.0000236
MZm_2	MBD-	0.0000557
PB25	MBD+	0.0564850
PB25	MBD-	0.0326214
PB44	MBD+	0.0217095
PB44	MBD-	0.0222526
PB9	MBD+	0.0456420
PB9	MBD-	0.0274122

Group	Average nuDNA coverage
average EQ MBD+	0.0001176
average EQ MBD-	0.0008695
average HS MBD+	0.0592719
average HS MBD-	0.2026680
average M MBD+	0.0012286
average M MBD-	0.0224536
average PB MBD+	0.0412788
average PB MBD-	0.0274287

TableS2.5_mtDNA per cell

Sample	Library	Size	M	Coverage (Q25)	Genome	mtDNA copies per cell
EQ26	MBD+	16,690	563	0.033732774	mtDNA	113.01
EQ26	MBD+	2,484,515,402	1,483,209	0.000596981	nuDNA	
EQ26	MBD-	16,690	12,766	0.764889155	mtDNA	771.20
EQ26	MBD-	2,484,515,402	4,928,353	0.001983628	nuDNA	
EQ36	MBD+	16,690	59	0.003535051	mtDNA	69.20
EQ36	MBD+	2,484,515,402	253,858	0.000102176	nuDNA	
EQ36	MBD-	16,690	1,014	0.060754943	mtDNA	191.09
EQ36	MBD-	2,484,515,402	1,579,848	0.000635878	nuDNA	
EQA	MBD+	16,690	25	0.001497903	mtDNA	59.02
EQA	MBD+	2,484,515,402	126,116	5.07608E-05	nuDNA	
EQA	MBD-	16,690	3,095	0.185440384	mtDNA	119.76
EQA	MBD-	2,484,515,402	7,694,366	0.003096928	nuDNA	
HSSR	MBD+	16,599	392,532	23.6479306	mtDNA	2.33
HSSR	MBD+	16,599	336,912	20.29712633	mtDNA	
HSSR	MBD-	3,095,693,981	99,620,831	0.032180452	nuDNA	2.38
HSSR	MBD-	3,095,693,981	83,866,952	0.027091487	nuDNA	
HSSR	MBD+	16,599	1,281,793	77.22109766	mtDNA	2.50
HSSR	MBD+	16,599	1,023,394	61.65395506	mtDNA	
HSSR	MBD-	3,095,693,981	345,427,095	0.111583088	nuDNA	2.45
HSSR	MBD-	3,095,693,981	281,971,075	0.091084932	nuDNA	
HSSU	MBD+	16,599	147,462	8.88378818	mtDNA	3.57
HSSU	MBD+	16,599	82,562	4.973914091	mtDNA	
HSSU	MBD-	3,095,693,981	25,124,623	0.008115991	nuDNA	3.66
HSSU	MBD-	3,095,693,981	13,732,079	0.004435865	nuDNA	
MKb1_1	MBD+	16,842	32,603	1.935815224	mtDNA	313.20
MKb1_1	MBD+	3,196,760,833	39,516,453	0.012361404	nuDNA	
MKb1_1	MBD-	16,842	1,095,277	65.03247833	mtDNA	1,056.94
MKb1_1	MBD-	3,196,760,833	393,387,065	0.123058022	nuDNA	
MKb1_2	MBD+	16,842	8,188	0.486165539	mtDNA	344.06
MKb1_2	MBD+	3,196,760,833	9,034,298	0.002826079	nuDNA	
MKb1_2	MBD-	16,842	306,172	18.17907612	mtDNA	1,141.67
MKb1_2	MBD-	3,196,760,833	101,805,287	0.031846388	nuDNA	
MKb2_1	MBD+	16,842	9,134	0.54233464	mtDNA	615.65
MKb2_1	MBD+	3,196,760,833	5,632,109	0.001761817	nuDNA	
MKb2_1	MBD-	16,842	1,318,596	78.29212683	mtDNA	1,443.39
MKb2_1	MBD-	3,196,760,833	346,796,322	0.108483662	nuDNA	
MKb2_2	MBD+	16,842	3,904	0.231801449	mtDNA	552.77
MKb2_2	MBD+	3,196,760,833	2,681,095	0.000838691	nuDNA	
MKb2_2	MBD-	16,842	366,106	21.73767961	mtDNA	1,489.68
MKb2_2	MBD-	3,196,760,833	93,295,722	0.029184455	nuDNA	
MKhs_1	MBD+	16,842	16,760	0.99513122	mtDNA	3,397.81
MKhs_1	MBD+	3,196,760,833	1,872,499	0.000585749	nuDNA	
MKhs_1	MBD-	16,842	1,871,631	111.1287852	mtDNA	4,533.67
MKhs_1	MBD-	3,196,760,833	156,717,351	0.049023796	nuDNA	
MKhs_2	MBD+	16,842	5,808	0.344852155	mtDNA	4,918.43
MKhs_2	MBD+	3,196,760,833	448,277	0.000140229	nuDNA	
MKhs_2	MBD-	16,842	546,528	32.45030281	mtDNA	6,125.19
MKhs_2	MBD-	3,196,760,833	33,871,868	0.010595684	nuDNA	
MKm_1	MBD+	16,842	5,044	0.299489372	mtDNA	2,927.07
MKm_1	MBD+	3,196,760,833	654,167	0.000204634	nuDNA	
MKm_1	MBD-	16,842	70,179	4.166904168	mtDNA	8,900.61
MKm_1	MBD-	3,196,760,833	2,993,186	0.000936318	nuDNA	
MKm_2	MBD+	16,842	6,250	0.371096069	mtDNA	4,785.16
MKm_2	MBD+	3,196,760,833	495,827	0.000155103	nuDNA	
MKm_2	MBD-	16,842	4,709	0.279598623	mtDNA	2,528.06
MKm_2	MBD-	3,196,760,833	707,110	0.000221196	nuDNA	
MKs_1	MBD+	16,842	20,463	1.214998219	mtDNA	12,906.92
MKs_1	MBD+	3,196,760,833	601,857	0.000188271	nuDNA	
MKs_1	MBD-	16,842	404,544	24.01995012	mtDNA	37,083.98
MKs_1	MBD-	3,196,760,833	4,141,197	0.001295435	nuDNA	
MKhs_2	MBD+	16,842	5,808	0.344852155	mtDNA	1,229.61
MKhs_2	MBD+	3,196,760,833	448,277	0.000140229	nuDNA	
MKhs_2	MBD-	16,842	546,528	32.45030281	mtDNA	1,531.30
MKhs_2	MBD-	3,196,760,833	33,871,868	0.010595684	nuDNA	
MZh_1	MBD+	16,842	3,356	0.199263745	mtDNA	6,760.11
MZh_1	MBD+	3,196,760,833	188,458	5.89528E-05	nuDNA	
MZh_1	MBD-	16,842	89,153	5.293492459	mtDNA	241,458.70
MZh_1	MBD-	3,196,760,833	140,165	4.38459E-05	nuDNA	
MZl_1	MBD+	16,842	332,999	19.7719392	mtDNA	229,761.63
MZl_1	MBD+	3,196,760,833	550,189	0.000172108	nuDNA	
MZl_1	MBD-	16,842	20,627,374	1224.757986	mtDNA	740,045.97
MZl_1	MBD-	3,196,760,833	10,581,122	0.003309951	nuDNA	
MZm_1	MBD+	16,842	18,112	1.075406721	mtDNA	8,484.07
MZm_1	MBD+	3,196,760,833	810,417	0.000253512	nuDNA	
MZm_1	MBD-	16,842	168,824	10.02398765	mtDNA	108,334.97
MZm_1	MBD-	3,196,760,833	591,578	0.000185055	nuDNA	
PB25	MBD+	16,898	12,282	0.726831578	mtDNA	25.74
PB25	MBD+	2,308,415,131	130,390,854	0.056485011	nuDNA	
PB25	MBD-	16,898	107,867	6.383418156	mtDNA	391.36
PB25	MBD-	2,308,415,131	75,303,621	0.032621351	nuDNA	
PB44	MBD+	16,898	294	0.017398509	mtDNA	1.60
PB44	MBD+	2,308,415,131	50,114,527	0.021709495	nuDNA	
PB44	MBD-	16,898	83,126	4.919280388	mtDNA	442.13
PB44	MBD-	2,308,415,131	51,368,258	0.022252608	nuDNA	
PB9	MBD+	16,898	37,729	2.232749438	mtDNA	97.84
PB9	MBD+	2,308,415,131	105,360,759	0.045642033	nuDNA	
PB9	MBD-	16,898	165,750	9.808853119	mtDNA	715.66
PB9	MBD-	2,308,415,131	63,278,814	0.027412233	nuDNA	
					**minimum**	**1.60**
					**maximum**	**740,045.97**
					**average**	**32,629.21**
					**median**	**914.07**

ZHENYA	Fraction	mtDNA copies per cell	mtDNA copies per cell liver / other tissue
Heart	MBD+	6,760	34.0
Muscle	MBD+	8,484	27.1
Liver	MBD+	229,762	
Heart	MBD-	241,459	3.1
Muscle	MBD-	108,335	6.8
Liver	MBD-	740,046	
		**min**	3.1
		**max**	34.0
		**average**	17.7

TableS3.1_CpG islands

ID	Fraction	Contig	Total Reads	Coverage CpG islands	Normalized coverage CpG islands	MBD+/MBD- normalized coverage	N reads CpG islands	Average CpGs per read	CpG density MBD+ / MBD-	CpG desnsity / CpG islands density
EQA	MBD+	CpG island	8,894,455	0.000540082	0.00035	0.09	469	0.1034	2.76	30.13
EQA	MBD-	CpG island	5,776,769	0.003828062	0.00383		4,425	0.0375		
EQ26	MBD+	CpG island	1,545,555	0.009946004	0.00148	0.88	5,075	0.1415	5.39	6.10
EQ26	MBD-	CpG island	230,002	0.001674115	0.00167		1,498	0.0262		
EQ34	MBD+	CpG island	989,551	0.001728083	0.00142	1.62	1,033	0.1335	4.36	2.68
EQ34	MBD-	CpG island	810,958	0.000872016	0.00087		1,044	0.0306		
EQ36	MBD+	CpG island	924,313	0.001810958	0.00168	2.70	911	0.1284	4.47	1.65
EQ36	MBD-	CpG island	855,798	0.000620896	0.00062		757	0.0287		
HSSR	MBD+	CpGIsland	43,779,216	0.365698804	0.16200	0.20	88,693	0.0485	1.21	6.05
HSSR	MBD-	CpGIsland	19,393,362	0.806191386	0.80619		251,844	0.0399		

minimum	1.65
maximum	30.13
average	9.32
median	6.05

TableS3.2_Non Unique reads

Sample	Library	N reads nuDNA Q0 (duplicates included)	N unique hits	N Non-unique hits	% N not uniquely mapped reads	MDB+ / MBD - N not uniquely mapped reads
EQ26	MBD+	65,036	12,452	52,584	80.85	4.58
EQ26	MBD-	85,669	70,546	15,123	17.65	
EQ34	MBD+	15,731	2,697	13,034	82.86	3.04
EQ34	MBD-	52,612	38,293	14,319	27.22	
EQ36	MBD+	13,373	2,318	11,055	82.67	3.50
EQ36	MBD-	41,413	31,629	9,784	23.63	
EQA	MBD+	10,316	2,038	8,278	80.24	4.34
EQA	MBD-	156,757	127,794	28,963	18.48	
HSSR	MBD+	7,870,985	2,921,566	4,949,419	62.88	1.85
HSSR	MBD-	17,378,429	11,467,585	5,910,844	34.01	
MKb1_1	MBD+	2,489,937	371,216	2,118,721	85.09	3.08
MKb1_1	MBD-	8,374,224	6,061,916	2,312,308	27.61	
MKb1_2	MBD+	232,093	56,334	175,759	75.73	3.16
MKb1_2	MBD-	1,503,187	1,142,914	360,273	23.97	
MKb2_1	MBD+	303,333	59,735	243,598	80.31	3.20
MKb2_1	MBD-	6,409,472	4,802,769	1,606,703	25.07	
MKb2_2	MBD+	50,668	24,897	25,771	50.86	2.10
MKb2_2	MBD-	1,492,012	1,131,396	360,616	24.17	
MKhs_1	MBD+	94,060	19,651	74,409	79.11	2.42
MKhs_1	MBD-	4,084,118	2,750,259	1,333,859	32.66	
MKhs_2	MBD+	7,400	2,667	4,733	63.96	2.18
MKhs_2	MBD-	641,243	452,718	188,525	29.40	
MKm_1	MBD+	19,413	7,674	11,739	60.47	2.30
MKm_1	MBD-	65,015	47,901	17,114	26.32	
MKm_2	MBD+	10,089	4,305	5,784	57.33	2.47
MKm_2	MBD-	13,426	10,304	3,122	23.25	
MKs_1	MBD+	23,997	9,077	14,920	62.17	1.86
MKs_1	MBD-	126,837	84,332	42,505	33.51	
MZh_1	MBD+	6,021	2,663	3,358	55.77	1.11
MZh_1	MBD-	4,082	2,034	2,048	50.17	
MZl_1	MBD+	17,153	6,893	10,260	59.81	0.93
MZl_1	MBD-	368,817	131,122	237,695	64.45	
MZm_1	MBD+	20,873	12,261	8,612	41.26	1.03
MZm_1	MBD-	15,421	9,243	6,178	40.06	
PB25	MBD+	827,556	714,143	113,413	13.70	1.31
PB25	MBD-	813,880	729,049	84,831	10.42	
PB44	MBD+	860,521	368,138	492,383	57.22	2.49
PB44	MBD-	655,810	505,391	150,419	22.94	
PB9	MBD+	754,424	611,460	142,964	18.95	1.23
PB9	MBD-	735,791	622,587	113,204	15.39	
					min	0.93
					max	4.58
					average	2.41
					median	2.36

TableS3.3_Repeated Elements

Sample	Sample	Contig	Fraction	N Bases Sample	N Bases Contig	Coverage	Relative Repeated elements coverage	Relative Repeated Elements coverage MBD+ / MBD-
EQ26	EQ26	Repeats	MBD+	3,806,530	1,811,090,450	0.002102	3.25	4.34
EQ26	EQ26	NonRepeats	MBD+	884,631	1,369,484,561	0.000646		
EQ26	EQ26	Repeats	MBD-	2,912,328	1,811,090,450	0.001608	0.75	
EQ26	EQ26	NonRepeats	MBD-	2,937,856	1,369,484,561	0.002145		
EQ34	EQ34	Repeats	MBD+	1,111,840	1,811,090,450	0.000614	5.27	5.98
EQ34	EQ34	NonRepeats	MBD+	159,466	1,369,484,561	0.000116		
EQ34	EQ34	Repeats	MBD-	1,404,581	1,811,090,450	0.000776	0.88	
EQ34	EQ34	NonRepeats	MBD-	1,204,916	1,369,484,561	0.000880		
EQ36	EQ36	Repeats	MBD+	1,053,801	1,811,090,450	0.000582	5.48	6.85
EQ36	EQ36	NonRepeats	MBD+	145,479	1,369,484,561	0.000106		
EQ36	EQ36	Repeats	MBD-	1,042,496	1,811,090,450	0.000576	0.80	
EQ36	EQ36	NonRepeats	MBD-	986,411	1,369,484,561	0.000720		
EQA	EQA	Repeats	MBD+	409,736	1,811,090,450	0.000226	3.08	4.49
EQA	EQA	NonRepeats	MBD+	100,536	1,369,484,561	0.000073		
EQA	EQA	Repeats	MBD-	4,324,658	1,811,090,450	0.002388	0.69	
EQA	EQA	NonRepeats	MBD-	4,766,846	1,369,484,561	0.003481		
HSSR	HSSR	Repeats	MBD+	197,097,136	6,574,197,005	0.029980	0.63	1.73
HSSR	HSSR	NonRepeats	MBD+	58,875,328	1,242,291,910	0.047393		
HSSR	HSSR	Repeats	MBD-	429,457,237	6,574,197,005	0.065325	0.37	
HSSR	HSSR	NonRepeats	MBD-	221,359,394	1,242,291,910	0.178186		
MKb1_1	MKb1_1	Repeats	MBD+	21,780,036	2,527,360,781	0.008618	0.79	1.05
MKb1_1	MKb1_1	NonRepeats	MBD+	16,647,212	1,528,205,064	0.010893		
MKb1_1	MKb1_1	Repeats	MBD-	245,904,563	2,527,360,781	0.097297	0.75	
MKb1_1	MKb1_1	NonRepeats	MBD-	198,201,113	1,528,205,064	0.129695		
MKb1_2	MKb1_2	Repeats	MBD+	3,755,865	2,527,360,781	0.001486	0.92	1.17
MKb1_2	MKb1_2	NonRepeats	MBD+	2,478,952	1,528,205,064	0.001622		
MKb1_2	MKb1_2	Repeats	MBD-	56,676,232	2,527,360,781	0.022425	0.79	
MKb1_2	MKb1_2	NonRepeats	MBD-	43,650,518	1,528,205,064	0.028563		
MKb2_1	MKb2_1	Repeats	MBD+	5,432,335	2,527,360,781	0.002149	0.68	0.89
MKb2_1	MKb2_1	NonRepeats	MBD+	4,814,759	1,528,205,064	0.003151		
MKb2_1	MKb2_1	Repeats	MBD-	217,562,660	2,527,360,781	0.086083	0.77	
MKb2_1	MKb2_1	NonRepeats	MBD-	171,931,278	1,528,205,064	0.112505		
MKb2_2	MKb2_2	Repeats	MBD+	1,697,047	2,527,360,781	0.000671	0.77	0.97
MKb2_2	MKb2_2	NonRepeats	MBD+	1,335,208	1,528,205,064	0.000874		
MKb2_2	MKb2_2	Repeats	MBD-	55,609,561	2,527,360,781	0.022003	0.79	
MKb2_2	MKb2_2	NonRepeats	MBD-	42,546,276	1,528,205,064	0.027841		
MKhs_1	MKhs_1	Repeats	MBD+	1,933,970	2,527,360,781	0.000765	0.69	0.96
MKhs_1	MKhs_1	NonRepeats	MBD+	1,701,515	1,528,205,064	0.001113		
MKhs_1	MKhs_1	Repeats	MBD-	105,221,449	2,527,360,781	0.041633	0.71	
MKhs_1	MKhs_1	NonRepeats	MBD-	89,031,331	1,528,205,064	0.058259		
MKhs_2	MKhs_2	Repeats	MBD+	280,439	2,527,360,781	0.000111	0.98	1.29
MKhs_2	MKhs_2	NonRepeats	MBD+	172,548	1,528,205,064	0.000113		
MKhs_2	MKhs_2	Repeats	MBD-	20,519,466	2,527,360,781	0.008119	0.76	
MKhs_2	MKhs_2	NonRepeats	MBD-	16,241,223	1,528,205,064	0.010628		
MKm_1	MKm_1	Repeats	MBD+	648,228	2,527,360,781	0.000256	0.77	1.10
MKm_1	MKm_1	NonRepeats	MBD+	511,335	1,528,205,064	0.000335		
MKm_1	MKm_1	Repeats	MBD-	2,055,304	2,527,360,781	0.000813	0.70	
MKm_1	MKm_1	NonRepeats	MBD-	1,776,488	1,528,205,064	0.001162		
MKm_2	MKm_2	Repeats	MBD+	387,022	2,527,360,781	0.000153	0.91	1.21
MKm_2	MKm_2	NonRepeats	MBD+	256,447	1,528,205,064	0.000168		
MKm_2	MKm_2	Repeats	MBD-	481,503	2,527,360,781	0.000191	0.75	
MKm_2	MKm_2	NonRepeats	MBD-	387,508	1,528,205,064	0.000254		
MKs_1	MKs_1	Repeats	MBD+	663,965	2,527,360,781	0.000263	0.87	1.22
MKs_1	MKs_1	NonRepeats	MBD+	464,123	1,528,205,064	0.000304		
MKs_1	MKs_1	Repeats	MBD-	3,026,639	2,527,360,781	0.001198	0.71	
MKs_1	MKs_1	NonRepeats	MBD-	2,570,743	1,528,205,064	0.001682		
MZh_1	MZh_1	Repeats	MBD+	164,676	2,527,360,781	0.000065	1.04	1.57
MZh_1	MZh_1	NonRepeats	MBD+	95,527	1,528,205,064	0.000063		
MZh_1	MZh_1	Repeats	MBD-	98,957	2,527,360,781	0.000039	0.66	
MZh_1	MZh_1	NonRepeats	MBD-	90,309	1,528,205,064	0.000059		
MZl_1	MZl_1	Repeats	MBD+	387,860	2,527,360,781	0.000153	0.75	1.09
MZl_1	MZl_1	NonRepeats	MBD+	313,478	1,528,205,064	0.000205		
MZl_1	MZl_1	Repeats	MBD-	5,591,317	2,527,360,781	0.002212	0.68	
MZl_1	MZl_1	NonRepeats	MBD-	4,940,508	1,528,205,064	0.003233		
MZm_1	MZm_1	Repeats	MBD+	599,222	2,527,360,781	0.000237	0.82	1.24
MZm_1	MZm_1	NonRepeats	MBD+	442,484	1,528,205,064	0.000290		
MZm_1	MZm_1	Repeats	MBD-	392,951	2,527,360,781	0.000155	0.66	
MZm_1	MZm_1	NonRepeats	MBD-	360,292	1,528,205,064	0.000236		
PB25	PB25	Repeats	MBD+	31,963,696	1,997,874,316	0.015999	0.31	0.76
PB25	PB25	NonRepeats	MBD+	77,143,763	1,496,108,866	0.051563		
PB25	PB25	Repeats	MBD-	27,013,999	1,997,874,316	0.013521	0.41	
PB25	PB25	NonRepeats	MBD-	49,422,943	1,496,108,866	0.033034		
PB44	PB44	Repeats	MBD+	20,326,376	1,997,874,316	0.010174	0.64	1.70
PB44	PB44	NonRepeats	MBD+	23,601,442	1,496,108,866	0.015775		
PB44	PB44	Repeats	MBD-	17,850,462	1,997,874,316	0.008935	0.38	
PB44	PB44	NonRepeats	MBD-	35,329,209	1,496,108,866	0.023614		
PB9	PB9	Repeats	MBD+	31,852,254	1,997,874,316	0.015943	0.38	0.81
PB9	PB9	NonRepeats	MBD+	63,130,459	1,496,108,866	0.042196		
PB9	PB9	Repeats	MBD-	25,154,980	1,997,874,316	0.012591	0.47	
PB9	PB9	NonRepeats	MBD-	40,496,036	1,496,108,866	0.027068		
							min	0.76
							max	6.85
							average	2.08
							median	1.21

TableS3.4_no CpG reads

Sample	Library	Ntot	N reads No-CpGs	% reads No-CpGs
EQ26	MBD+	12,467	359	2.88
EQ26	MBD-	70,561	36,367	51.54
EQ34	MBD+	2,700	230	8.52
EQ34	MBD-	38,301	20,844	54.42
EQ36	MBD+	2,325	164	7.05
EQ36	MBD-	31,633	18,145	57.36
EQA	MBD+	2,041	631	30.92
EQA	MBD-	127,821	56,369	44.10
HSSR	MBD+	2,923,436	1,352,845	46.28
HSSR	MBD-	11,469,740	5,806,157	50.62
MKb1_1	MBD+	372,725	49,889	13.38
MKb1_1	MBD-	6,064,924	3,055,142	50.37
MKb1_2	MBD+	56,553	10,045	17.76
MKb1_2	MBD-	1,143,569	590,194	51.61
MKb2_1	MBD+	59,822	11,841	19.79
MKb2_1	MBD-	4,805,331	2,438,052	50.74
MKb2_2	MBD+	24,916	5,835	23.42
MKb2_2	MBD-	1,132,076	597,201	52.75
MKhs_1	MBD+	19,689	5,004	25.42
MKhs_1	MBD-	2,751,525	1,331,259	48.38
MKhs_2	MBD+	2,672	834	31.21
MKhs_2	MBD-	452,970	229,760	50.72
MKm_1	MBD+	7,681	2,278	29.66
MKm_1	MBD-	47,930	24,998	52.16
MKm_2	MBD+	4,310	1,630	37.82
MKm_2	MBD-	10,311	5,618	54.49
MKs_1	MBD+	9,087	3,611	39.74
MKs_1	MBD-	84,371	46,350	54.94
MZh_1	MBD+	2,664	1,300	48.80
MZh_1	MBD-	2,038	918	45.04
MZl_1	MBD+	6,899	3,188	46.21
MZl_1	MBD-	131,278	58,580	44.62
MZm_1	MBD+	12,269	5,916	48.22
MZm_1	MBD-	9,259	3,877	41.87
PB25	MBD+	714,331	10,191	1.43
PB25	MBD-	729,183	300,046	41.15
PB44	MBD+	368,682	3,644	0.99
PB44	MBD-	505,586	124,649	24.65
PB9	MBD+	611,655	19,696	3.22
PB9	MBD-	622,807	268,876	43.17

		% reads No-CpGs MBD+
Equids	min	2.88
	max	30.92
	average	12.34
Saqqaq	min	46.28
	max	46.28
	avg	46.28
Mammoth	min	13.38
	max	48.80
	avg	31.79
Polar Bear	min	0.99
	max	3.22
	avg	1.88

TableS3.5 Ms Saqqaq

			Ms					
Sample	Library	Threshold density	Minimum	1st quantile	Median	Mean	2nd quantile	Maximum
HSSR	MBD+	50	0.000	0.000	0.019	0.029	0.050	0.736
HSSR	MBD-	50	0.000	0.000	0.015	0.023	0.037	0.736
HSSR	MBD+	60	0.000	0.012	0.032	0.034	0.056	0.736
HSSR	MBD-	60	0.000	0.000	0.017	0.028	0.048	0.736

TableS3.6_CpG.CpT

Sample	MBD	CG>TG	CG>CG	CG>AG	CG>GG	CA>TA	CT>TT	CC>TC	CN>TN	(CG>TG)/(CN>TN)	(CG>TG)/(CN>TN) MBD+/MBD-
EQA	MBD+	26	536	2	0	26	29	35	116	0.2241	6.31
EQA	MBD-	278	4,180	12	18	2,598	3,060	1,890	7,826	0.0355	
EQ26	MBD+	212	6,562	13	6	254	284	408	1,158	0.1831	7.51
EQ26	MBD-	108	1,665	1	3	1,585	1,803	932	4,428	0.0244	
EQ34	MBD+	15	1,281	1	0	11	10	11	47	0.3191	8.26
EQ34	MBD-	17	941	3	5	139	162	122	440	0.0386	
EQ36	MBD+	28	1,036	0	3	32	39	46	145	0.1931	7.75
EQ36	MBD-	25	676	0	3	359	367	252	1,003	0.0249	
MKm_2	MBD+	6	603	2	6	43	36	29	114	0.0526	0.96
MKm_2	MBD-	6	124	0	1	41	46	16	109	0.0550	
MKs_2	MBD+	5	169	0	1	20	17	12	54	0.0926	1.62
MKs_2	MBD-	13	158	1	0	84	80	50	227	0.0573	
MKhs_2	MBD+	11	791	4	3	32	36	24	103	0.1068	2.20
MKhs_2	MBD-	415	7,465	53	42	2,988	3,050	2,108	8,561	0.0485	
MKb2_2	MBD+	98	3,446	10	8	214	240	156	708	0.1384	3.60
MKb2_2	MBD-	877	14,272	123	85	8,897	8,514	4,494	22,782	0.0385	
MKb1_2	MBD+	570	12,837	345	366	726	953	843	3,092	0.1843	4.20
MKb1_2	MBD-	936	15,798	140	103	7,892	7,916	4,588	21,332	0.0439	
MKb2_1	MBD+	265	11,932	42	64	541	520	506	1,832	0.1447	3.46
MKb2_1	MBD-	4,369	73,542	578	519	38,732	38,273	23,094	104,468	0.0418	
MKb1_1	MBD+	3,404	88,370	2,417	2,926	3,207	5,277	5,236	17,124	0.1988	3.02
MKb1_1	MBD-	14,216	84,834	10,443	10,967	65,083	79,129	57,220	215,648	0.0659	
MKhs_1	MBD+	112	7,527	17	47	136	143	173	564	0.1986	4.10
MKhs_1	MBD-	2,438	63,122	395	427	16,666	16,344	14,893	50,341	0.0484	
MKm_1	MBD+	30	2,114	5	6	33	61	50	174	0.1724	1.95
MKm_1	MBD-	54	1,044	6	2	192	220	146	612	0.0882	
MKs_1	MBD+	30	1,281	1	3	67	65	77	239	0.1255	2.80
MKs_1	MBD-	73	1,759	11	14	520	588	447	1,628	0.0448	
PB25	MBD+	9,224	85,796	8,562	11,346	7,568	13,014	19,002	48,808	0.1890	3.19
PB25	MBD-	1,479	14,191	1,417	1,476	6,772	10,072	6,651	24,974	0.0592	
PB44	MBD+	6,977	57,760	6,061	10,162	2,982	5,243	11,861	27,063	0.2578	3.78
PB44	MBD-	920	23,477	511	640	3,161	4,449	4,977	13,507	0.0681	
PB9	MBD+	3,362	105,794	2,910	3,950	4,114	5,953	7,666	21,095	0.1594	2.74
PB9	MBD-	2,220	6,197	2,108	1,957	10,971	15,797	9,204	38,192	0.0581	
MZh_1	MBD+	5	114	1	1	17	24	12	58	0.0862	3.19
MZh_1	MBD-	1	113	2	4	14	13	9	37	0.0270	
MZl_1	MBD+	12	306	2	2	52	63	40	167	0.0719	1.71
MZl_1	MBD-	184	3,034	14	12	1,475	1,734	977	4,370	0.0421	
MZm_1	MBD+	8	425	2	4	70	98	59	235	0.0340	0.58
MZm_1	MBD-	10	513	6	2	46	66	49	171	0.0585	
HSSR	MBD+	2,361	252,940	255	306	9,064	8,532	11,703	31,660	0.0746	1.37
HSSR	MBD-	6,274	511,100	727	615	35,026	31,062	42,579	114,941	0.0546	
										min	0.58
										max	8.26
										average	3.49
										median	3.10

			Collapsed Reads			
			mtDNA		nuDNA	
Sample	Fraction	Total Reads	Uniquely mapped Q0	Uniquely mapped Q25	Uniquely mapped Q0	Uniquely mapped Q25
EQ26	MBD+	1,545,555	5	5	65,036	12,467
EQ26	MBD-	230,002	192	190	85,669	70,561
EQ34	MBD+	989,551	0	0	15,731	2,700
EQ34	MBD-	810,958	4	4	52,612	38,301
EQ36	MBD+	924,313	1	1	13,373	2,325
EQ36	MBD-	855,798	22	22	41,413	31,633
EQA	MBD+	8,894,455	1	1	10,316	2,041
EQA	MBD-	5,776,769	48	48	156,757	127,821
EQB	MBD+	10,136,112	3	3	4,104	942
EQB	MBD-	7,000,618	2	2	2,797	1,303
EQC	MBD+	10,509,471	4	4	6,450	1,661
EQC	MBD-	7,065,549	1	1	4,173	2,667
EQE	MBD+	8,435,891	0	0	3,526	855
EQE	MBD-	9,195,063	1	1	2,568	1,603
EQF	MBD+	10,060,504	0	0	3,628	1,078
EQF	MBD-	7,550,930	93	93	20,181	16,329
HSSR	MBD+	43,779,216	11,720	11,719	7,870,985	2,923,436
HSSR	MBD-	19,393,362	41,760	41,758	17,378,429	11,469,740
MKb1_1	MBD+	10,461,328	360	337	2,489,937	372,725
MKb1_1	MBD-	12,872,086	17,030	16,509	8,374,224	6,064,924
MKb1_2	MBD+	2,385,434	69	68	232,093	56,553
MKb1_2	MBD-	2,770,052	3,324	3,271	1,503,187	1,143,569
MKb2_1	MBD+	11,878,035	141	110	303,333	59,822
MKb2_1	MBD-	11,636,012	18,899	18,458	6,409,472	4,805,331
MKb2_2	MBD+	2,800,110	32	30	50,668	24,916
MKb2_2	MBD-	2,989,196	4,531	4,455	1,492,012	1,132,076
MKhs_1	MBD+	11,628,856	161	149	94,060	19,689
MKhs_1	MBD-	12,679,846	27,430	26,666	4,084,118	2,751,525
MKhs_2	MBD+	5,239,477	21	21	7,400	2,672
MKhs_2	MBD-	2,836,386	5,538	5,441	641,243	452,970
MKm_1	MBD+	11,966,724	48	43	19,413	7,681
MKm_1	MBD-	16,177,223	961	928	65,015	47,930
MKm_2	MBD+	15,533,439	49	49	10,089	4,310
MKm_2	MBD-	3,069,468	70	67	13,426	10,311
MKs_1	MBD+	12,949,142	316	296	23,997	9,087
MKs_1	MBD-	20,004,345	8,022	7,627	126,837	84,371
MKs_2	MBD+	2,890,744	83	78	3,277	1,717
MKs_2	MBD-	2,717,680	1,446	1,401	22,037	15,583
MZh_1	MBD+	7,171,605	31	29	6,021	2,664
MZh_1	MBD-	8,067,779	737	724	4,082	2,038
MZh_2	MBD+	2,035,979	4	4	882	505
MZh_2	MBD-	903,182	51	51	290	162
MZl_1	MBD+	8,702,102	2,808	2,499	17,153	6,899
MZl_1	MBD-	9,004,754	253,458	246,537	368,817	131,278
MZl_2	MBD+	2,516,242	526	517	1,187	423
MZl_2	MBD-	1,901,793	43,626	42,895	60,427	21,078
MZm_1	MBD+	10,819,017	176	172	20,873	12,269
MZm_1	MBD-	13,728,406	2,457	2,407	15,421	9,259
MZm_2	MBD+	2,135,223	16	15	529	283
MZm_2	MBD-	3,103,006	523	519	2,586	1,653
PB25	MBD+	2,107,980	59	53	827,556	714,331
PB25	MBD-	1,569,088	911	901	813,880	729,183
PB44	MBD+	1,590,587	6	5	860,521	368,682
PB44	MBD-	1,692,855	712	690	655,810	505,586
PB9	MBD+	1,859,912	186	183	754,424	611,655
PB9	MBD-	1,538,441	1,713	1,702	735,791	622,807

TableS4.1_Len CpG CG >2,000

Sample	Library	Nreads	avgLen	avgLn MBD+ - avgLen MBD-	sdLen	minLen	lowLen	midLen	topLen	maxLen	avgGC	avgGC MBD+ / avgGC MBD-	sdGC	minGC	lowGC	midGC	topGC	maxGC	avgCpG	avgCpG MBD+ / avgCpG MBD-	sdCpG	minCpG	lowCpG	midCpG	topCpG	maxCpG
EQ26	MBD+	12,466	115.47	45.67	39.4762	34	88	113	140	234	0.6262	1.46	0.0820	0.3471	0.5882	0.6316	0.6754	0.7964	0.1415	5.39	0.0665	0.0000	0.1017	0.1379	0.1778	0.3333
EQ26	MBD-	70,560	69.80		24.5144	29	52	66	84	141	0.4288		0.1094	0.2154	0.3478	0.4202	0.5000	0.6944	0.0262		0.0376	0.0000	0.0000	0.0129	0.0400	0.1644
EQ34	MBD+	2,699	92.14	41.17	41.0345	25	64	85	112	229	0.6063	1.33	0.1014	0.3091	0.5580	0.6190	0.6667	0.8205	0.1335	4.36	0.0847	0.0000	0.0714	0.1351	0.1856	0.3590
EQ34	MBD-	38,300	50.97		17.1994	25	38	48	60	105	0.4543		0.1138	0.2246	0.3714	0.4483	0.5319	0.7308	0.0306		0.0464	0.0000	0.0000	0.0000	0.0488	0.2000
EQ36	MBD+	2,324	105.48	55.55	45.3997	25	73	101	132	234	0.6005	1.37	0.0921	0.3094	0.5624	0.6120	0.6538	0.8070	0.1284	4.47	0.0767	0.0000	0.0800	0.1300	0.1754	0.3254
EQ36	MBD-	31,632	49.94		17.5906	25	38	46	58	110	0.4384		0.1146	0.2105	0.3542	0.4297	0.5152	0.7200	0.0287		0.0452	0.0000	0.0000	0.0000	0.0476	0.2032
EQA	MBD+	2,040	58.33	-1.83	32.7125	24	33	50	73	162	0.5547	1.15	0.1624	0.2108	0.4348	0.5600	0.6776	0.8925	0.1034	2.76	0.1118	0.0000	0.0000	0.0769	0.1639	0.4733
EQA	MBD-	127,820	60.16		23.6274	26	43	55	72	136	0.4815		0.1119	0.2576	0.4000	0.4750	0.5577	0.7586	0.0375		0.0511	0.0000	0.0000	0.0263	0.0541	0.2308
HSSR	MBD+	2,923,435	58.59	5.06	25.9706	25	39	52	72	138	0.4961	1.01	0.1282	0.2500	0.4000	0.4872	0.5784	0.8378	0.0485	1.21	0.0774	0.0000	0.0000	0.0227	0.0625	0.3778
HSSR	MBD-	11,469,739	53.53		22.9235	25	36	48	65	129	0.4921		0.1214	0.2549	0.4035	0.4857	0.5714	0.8182	0.0399		0.0653	0.0000	0.0000	0.0000	0.0556	0.3256
MKb1_1	MBD+	372,724	99.01	34.26	45.7377	29	64	92	125	239	0.5142	1.18	0.1218	0.2430	0.4314	0.5119	0.5972	0.8298	0.0961	3.69	0.0863	0.0000	0.0351	0.0808	0.1364	0.4400
MKb1_1	MBD-	6,064,923	64.74		26.4893	28	46	59	78	151	0.4366		0.0965	0.2353	0.3684	0.4324	0.5000	0.6842	0.0261		0.0376	0.0000	0.0000	0.0000	0.0417	0.1622
MKb1_2	MBD+	56,552	83.75	10.62	27.4326	31	62	84	105	136	0.4928	1.22	0.1184	0.2267	0.4110	0.4940	0.5802	0.7949	0.0854	4.02	0.0780	0.0000	0.0260	0.0734	0.1280	0.3824
MKb1_2	MBD-	1,143,568	73.12		26.2175	31	52	69	91	134	0.4055		0.0911	0.2143	0.3412	0.4016	0.4656	0.6341	0.0212		0.0299	0.0000	0.0000	0.0000	0.0351	0.1250
MKb2_1	MBD+	59,821	87.75	15.78	41.8768	26	55	81	113	217	0.4919	1.16	0.1268	0.2200	0.4074	0.4835	0.5673	0.8302	0.0793	3.45	0.0830	0.0000	0.0230	0.0625	0.1073	0.4286
MKb2_1	MBD-	4,805,330	71.97		30.1808	28	49	66	89	161	0.4238		0.0954	0.2222	0.3571	0.4200	0.4857	0.6667	0.0230		0.0332	0.0000	0.0000	0.0000	0.0370	0.1429
MKb2_2	MBD+	24,915	78.50	6.64	28.5026	30	55	76	101	135	0.4655	1.14	0.1116	0.2154	0.3917	0.4648	0.5345	0.7447	0.0659	3.20	0.0645	0.0000	0.0169	0.0556	0.0980	0.2857
MKb2_2	MBD-	1,132,075	71.86		26.2645	30	51	68	90	134	0.4093		0.0917	0.2128	0.3451	0.4063	0.4706	0.6364	0.0206		0.0293	0.0000	0.0000	0.0000	0.0345	0.1231
MKhs_1	MBD+	19,688	78.65	21.81	47.1439	25	45	65	97	245	0.5331	1.13	0.1634	0.2241	0.4167	0.5059	0.6333	0.9211	0.1101	3.38	0.1388	0.0000	0.0000	0.0571	0.1429	0.5532
MKhs_1	MBD-	2,751,524	56.84		26.0761	26	38	50	68	149	0.4711		0.1034	0.2569	0.4000	0.4658	0.5360	0.7400	0.0326		0.0473	0.0000	0.0000	0.0179	0.0513	0.2162
MKhs_2	MBD+	2,671	70.58	7.22	27.7027	29	47	67	90	135	0.4969	1.13	0.1495	0.2179	0.3889	0.4805	0.5862	0.8684	0.0862	3.36	0.1182	0.0000	0.0000	0.0435	0.1134	0.5268
MKhs_2	MBD-	452,969	63.35		25.5967	29	43	58	78	132	0.4394		0.0963	0.2373	0.3714	0.4359	0.5000	0.6829	0.0257		0.0364	0.0000	0.0000	0.0000	0.0426	0.1579
MKm_1	MBD+	7,680	79.37	17.65	39.3545	24	50	72	101	202	0.502	1.19	0.1530	0.2000	0.3913	0.4872	0.6000	0.8732	0.0853	3.29	0.1150	0.0000	0.0000	0.0421	0.1132	0.5000
MKm_1	MBD-	47,929	61.72		19.1777	28	48	59	72	120	0.4236		0.1022	0.2206	0.3500	0.4167	0.4894	0.6889	0.0260		0.0394	0.0000	0.0000	0.0000	0.0400	0.1724
MKm_2	MBD+	4,309	75.95	9.22	26.9377	28	54	73	96	135	0.4514	1.15	0.1285	0.1842	0.3600	0.4444	0.5294	0.7953	0.0533	2.57	0.0799	0.0000	0.0000	0.0274	0.0732	0.4090
MKm_2	MBD-	10,310	66.73		20.1297	33	52	64	78	124	0.3939		0.0955	0.2027	0.3261	0.3878	0.4583	0.6292	0.0207		0.0301	0.0000	0.0000	0.0000	0.0345	0.1224
MKs_1	MBD+	9,086	60.57	12.05	31.2869	24	38	53	74	171	0.4695	1.06	0.1401	0.1923	0.3738	0.4565	0.5484	0.8462	0.0627	2.07	0.0943	0.0000	0.0000	0.0323	0.0833	0.4659
MKs_1	MBD-	84,370	48.51		17.2765	25	37	45	57	106	0.4411		0.1081	0.2200	0.3659	0.4340	0.5085	0.7407	0.0302		0.0495	0.0000	0.0000	0.0000	0.0488	0.2308
MKs_2	MBD+	1,716	63.14	4.15	25.7963	27	42	58	79	132	0.4374	1.09	0.1237	0.1752	0.3500	0.4303	0.5167	0.7629	0.0506	2.33	0.0709	0.0000	0.0000	0.0276	0.0759	0.3099
MKs_2	MBD-	15,582	58.98		22.2003	28	43	54	70	127	0.402		0.0959	0.2045	0.3333	0.3966	0.4667	0.6452	0.0217		0.0328	0.0000	0.0000	0.0000	0.0385	0.1379
MZh_1	MBD+	2,663	57.27	-0.21	26.9253	24	37	51	71	142	0.4609	0.99	0.1223	0.2167	0.3810	0.4524	0.5263	0.8468	0.0374	0.93	0.0700	0.0000	0.0000	0.0000	0.0493	0.3750
MZh_1	MBD-	2,037	57.48		34.2539	24	34	49	69	176	0.4655		0.1237	0.2314	0.3768	0.4545	0.5385	0.8400	0.0401		0.0701	0.0000	0.0000	0.0159	0.0526	0.3945
MZl_1	MBD+	6,898	65.68	-14.24	33.1126	25	42	58	80	181	0.4452	1.05	0.1134	0.2222	0.3684	0.4348	0.5102	0.7943	0.0343	1.28	0.0600	0.0000	0.0000	0.0171	0.0465	0.3243
MZl_1	MBD-	131,277	79.92		38.1731	28	51	70	100	194	0.4243		0.0929	0.2407	0.3604	0.4154	0.4805	0.6812	0.0268		0.0391	0.0000	0.0000	0.0179	0.0408	0.1667
MZm_1	MBD+	12,268	57.00	-4.24	26.0464	25	38	50	69	147	0.467	0.98	0.1119	0.2418	0.3902	0.4600	0.5341	0.7895	0.0350	0.76	0.0588	0.0000	0.0000	0.0146	0.0513	0.3063
MZm_1	MBD-	9,258	61.24		31.5552	24	39	53	75	166	0.4774		0.1222	0.2500	0.3939	0.4643	0.5385	0.8772	0.0463		0.0781	0.0000	0.0000	0.0249	0.0571	0.4231
PB25	MBD+	714,330	172.64	70.06	90.3444	43	104	152	223	437	0.5672	1.32	0.0903	0.3623	0.5028	0.5703	0.6333	0.7600	0.0849	3.35	0.0537	0.0000	0.0461	0.0741	0.1129	0.2564
PB25	MBD-	729,182	102.57		60.1037	32	61	86	126	322	0.4287		0.1083	0.2151	0.3488	0.4189	0.5000	0.6912	0.0253		0.0337	0.0000	0.0000	0.0165	0.0375	0.1515
PB44	MBD+	368,681	134.62	33.39	64.6116	39	90	120	164	353	0.6358	1.28	0.0825	0.3727	0.5933	0.6475	0.6891	0.8000	0.1274	3.27	0.0612	0.0000	0.0845	0.1205	0.1613	0.3088
PB44	MBD-	505,585	101.23		53.5881	36	64	87	122	296	0.4986		0.0969	0.2953	0.4273	0.4961	0.5676	0.7213	0.0389		0.0418	0.0000	0.0079	0.0292	0.0548	0.1923
PB9	MBD+	611,654	166.67	65.52	83.2414	42	106	152	207	422	0.5408	1.30	0.0996	0.3214	0.4678	0.5443	0.6154	0.7463	0.0775	3.24	0.0519	0.0000	0.0399	0.0674	0.1053	0.2400
PB9	MBD-	622,806	101.15		54.9277	33	62	87	125	300	0.4147		0.1071	0.2020	0.3371	0.4046	0.4853	0.6786	0.0239		0.0324	0.0000	0.0000	0.0152	0.0357	0.1458

**average Equids**	35.14
**average Mammoth**	9.29
**average Polar Bear**	56.32
**Saqqaq**	5.06
**Overall Average**	20.73

99.4	Difference in the verage length of endogenous DNA inserts in MBD+ of Polar Bear and Paleo-Eskimo
48.1	Difference in the verage length of endogenous DNA inserts in MBD- of Polar Bear and Paleo-Eskimo

TableS4.2_p-values lmm

		p-value		
Specie	Sample	read length	%GC	CpG density
Mammuthus primigenius	MKb2_1	2.5E-05	6.0E-06	4.2E-06
	MKb2_2	2.8E-04	1.7E-05	9.8E-06
	MKb1_1	1.4E-06	3.0E-06	1.4E-06
	MKb1_2	3.3E-05	3.9E-06	2.9E-06
	MKhs_1	4.2E-05	3.2E-05	1.9E-05
	MKhs_2	1.0E-04	2.1E-05	2.6E-06
	MKm_1	2.3E-05	2.8E-05	1.2E-05
	MKm_2	2.7E-04	1.5E-04	1.0E-04
	MKs_1	1.4E-04	2.1E-04	1.1E-04
	MZh_1	8.1E-01	2.1E-01	2.0E-01
	MZl_1	6.6E-05	2.0E-04	2.9E-04
	MZm_1	1.4E-03	4.4E-03	1.1E-03
Ursus maritimus	PB25	1.1E-06	5.2E-07	5.2E-07
	PB44	2.5E-06	4.8E-07	3.5E-07
	PB9	1.1E-06	7.1E-07	6.3E-07
Equus caballus	EQ26	2.9E-06	2.3E-06	1.1E-06
	EQ34	5.6E-06	1.4E-05	5.7E-06
	EQ36	4.0E-06	1.5E-05	6.7E-06
Equus Lambei	EQA	4.0E-03	4.0E-05	1.0E-05
Homo sapiens	Saqqaq	7.3E-06	5.7E-06	1.1E-04

TableS4.3_DeltaS DeltaD Overhan

Sample	Library	Genome	minDeltaD	lowDeltaD	midDeltaD	midDeltaD MBD- / midDeltaD MBD+	topDeltaD	maxDeltaD	minDeltaS	lowDeltaS	midDeltaS	midDeltaS MBD- / midDeltaS MBD+	topDeltaS	maxDeltaS	minOverhang	lowOverhang	midOverhang	topOverhang	maxOverhang
EQ26	MBD+	Nuclear	0.031107	0.033763	0.034808	1.548	0.035811	0.038177	0.295088	0.321148	0.332993	1.124	0.345306	0.379554	1.409888286	1.681884103	1.80329599	1.930228668	2.278626085
EQ26	MBD-	Nuclear	0.051994	0.053358	0.053884		0.054404	0.055704	0.349815	0.367207	0.374329		0.382017	0.402014	1.359542087	1.505901828	1.563862561	1.623389287	1.79486835
EQ34	MBD+	Nuclear	0.000256	0.001785	0.002515	1.893	0.003233	0.005082	0.145641	0.189597	0.21244	1.177	0.238747	0.323062	0.671424708	0.968415396	1.116486998	1.283827943	1.77889432
EQ34	MBD-	Nuclear	0.003995	0.00454	0.004762		0.004982	0.005517	0.226091	0.242586	0.250117		0.257433	0.277969	0.974878929	1.068625492	1.108097813	1.151107078	1.258808457
EQ36	MBD+	Nuclear	0.015547	0.019753	0.021304	1.043	0.022746	0.026087	0.19177	0.244646	0.271088	1.074	0.302389	0.399771	0.739305656	1.114665126	1.309183226	1.545127114	2.347169071
EQ36	MBD-	Nuclear	0.020402	0.021697	0.022226		0.022744	0.023969	0.264793	0.283258	0.291238		0.29948	0.321236	1.385514297	1.549468502	1.621468589	1.695728773	1.896958809
EQA	MBD+	Nuclear	0.013556	0.022455	0.025253	1.618	0.02775	0.03311	0.224094	0.288155	0.321831	1.397	0.361445	0.505453	0.751713142	1.289661125	1.567506436	1.933336621	3.28251488
EQA	MBD-	Nuclear	0.039598	0.040494	0.040849		0.041209	0.04203	0.432438	0.444436	0.449696		0.455042	0.468858	1.51648757	1.59825286	1.632283531	1.666661956	1.751456753
HSSR	MBD+	Nuclear	0.004346	0.004397	0.004418	0.973	0.004439	0.00449	0.194761	0.197425	0.198506	0.921	0.199594	0.202387	0.730018857	0.742449661	0.74737441	0.752536251	0.76478047
HSSR	MBD-	Nuclear	0.004261	0.004285	0.004296		0.004307	0.004334	0.181412	0.182433	0.182863		0.183306	0.184316	0.959961053	0.966382014	0.969359944	0.972185027	0.978755844
MKb1_1	MBD+	Nuclear	0.002146	0.002647	0.002856	3.377	0.003069	0.003539	0.187185	0.190832	0.192392	1.057	0.193926	0.197718	2.167302091	2.251564416	2.287919332	2.325417712	2.416922389
MKb1_1	MBD-	Nuclear	0.009538	0.009616	0.009645		0.009677	0.009755	0.201702	0.202911	0.2034		0.203884	0.205123	1.490294943	1.504953667	1.510932791	1.516681297	1.531312985
MKb1_2	MBD+	Nuclear	0.004953	0.006307	0.006815	1.863	0.007317	0.008373	0.097168	0.102269	0.104417	1.605	0.106746	0.112665	2.397718178	2.734105456	2.897154869	3.063948598	3.532325111
MKb1_2	MBD-	Nuclear	0.012425	0.012618	0.012694		0.012769	0.012954	0.163907	0.166468	0.167561		0.168581	0.171073	1.554464043	1.596108094	1.61365938	1.631484554	1.677421683
MKb2_1	MBD+	Nuclear	0.007333	0.00812	0.008436	1.536	0.008735	0.009472	0.257826	0.274347	0.28101	1.040	0.2878	0.30665	1.065913722	1.171521161	1.214131783	1.258968975	1.382579301
MKb2_1	MBD-	Nuclear	0.012831	0.01292	0.012957		0.012991	0.013079	0.289437	0.291362	0.292206		0.293072	0.29514	1.183240812	1.196306342	1.201619808	1.207025265	1.219707941
MKb2_2	MBD+	Nuclear	0.00783	0.009201	0.009705	1.379	0.010188	0.011306	0.143487	0.158925	0.165928	1.225	0.173295	0.193841	1.180390291	1.420126093	1.533185726	1.653758063	1.992800116
MKb2_2	MBD-	Nuclear	0.0131	0.013299	0.013378		0.013458	0.013667	0.199112	0.202048	0.203263		0.204501	0.207647	1.494308245	1.534656773	1.550994214	1.567288326	1.606673251
MKhs_1	MBD+	Nuclear	0.000713	0.002199	0.002779	2.401	0.003368	0.004754	0.15133	0.168813	0.176758	1.056	0.184836	0.207961	1.249471627	1.511815522	1.629795788	1.75797452	2.110075843
MKhs_1	MBD-	Nuclear	0.006474	0.006613	0.006671		0.006726	0.00687	0.184363	0.185941	0.186593		0.187255	0.188923	1.990612663	2.020864819	2.033004089	2.045178154	2.075160351
MKhs_2	MBD+	Nuclear	0.001984	0.005094	0.006156	1.476	0.007191	0.009482	0.074565	0.095428	0.106449	1.437	0.119288	0.162012	0.878229418	1.413884383	1.711674904	2.077454747	3.405837913
MKhs_2	MBD-	Nuclear	0.008546	0.008934	0.009087		0.009241	0.009609	0.14833	0.151677	0.153008		0.154343	0.1577	2.114241606	2.205203779	2.242301229	2.280021523	2.383661447
MKm_1	MBD+	Nuclear	0.003417	0.005578	0.00641	1.414	0.00721	0.009192	0.184056	0.224269	0.243897	0.788	0.266579	0.336584	0.67719737	0.942719581	1.071570591	1.219117052	1.662539811
MKm_1	MBD-	Nuclear	0.007863	0.008728	0.009061		0.009397	0.010191	0.171186	0.185674	0.192254		0.199149	0.21811	1.100576668	1.266203249	1.336717737	1.41362124	1.609777917
MKm_2	MBD+	Nuclear	0.006967	0.009238	0.01013	0.880	0.010994	0.013127	0.125505	0.160748	0.181244	0.890	0.206188	0.297824	0.495265671	0.832563163	1.006043181	1.210057781	1.773620455
MKm_2	MBD-	Nuclear	0.006096	0.008171	0.008913		0.009667	0.011357	0.127114	0.150378	0.161266		0.172947	0.206356	1.108070884	1.443151332	1.605656773	1.791118414	2.391839067
MKs_1	MBD+	Nuclear	0.006971	0.010019	0.011116	0.983	0.012128	0.014586	0.156619	0.17995	0.190361	1.072	0.201867	0.235476	1.34085507	1.743913481	1.939657741	2.151047879	2.838034371
MKs_1	MBD-	Nuclear	0.009884	0.010617	0.010923		0.011218	0.011937	0.190801	0.200086	0.204082		0.208203	0.218699	1.55750438	1.687161556	1.743118908	1.802296222	1.952956478
MZh_1	MBD+	Nuclear	0.000296	0.003633	0.005681	1.556	0.007653	0.012041	0.093273	0.11833	0.131459	1.423	0.146748	0.202444	1.013176799	1.900239015	2.367708091	2.932742543	4.404519825
MZh_1	MBD-	Nuclear	0.001814	0.007054	0.008838		0.010514	0.014419	0.11069	0.156394	0.187076		0.224739	0.384605	0.402939219	0.886912161	1.185605947	1.593328868	3.084387453
MZl_1	MBD+	Nuclear	0.003012	0.007108	0.008501	1.719	0.009819	0.012663	0.138296	0.15805	0.167332	1.751	0.177715	0.207898	1.546417657	2.074790749	2.339700885	2.646586973	3.589829061
MZl_1	MBD-	Nuclear	0.013654	0.014329	0.014609		0.014887	0.015564	0.280164	0.289078	0.29293		0.296859	0.306782	1.558713177	1.642467465	1.679730006	1.716421954	1.809332091
MZm_1	MBD+	Nuclear	0.00425	0.006552	0.007358	0.894	0.008116	0.009907	0.147493	0.168361	0.177917	1.185	0.187949	0.21568	1.304316548	1.643372398	1.803349852	1.984155598	2.555519428
MZm_1	MBD-	Nuclear	0.00302	0.005584	0.006579		0.007493	0.009635	0.171575	0.197524	0.210852		0.225971	0.272117	1.041786011	1.404613075	1.576094988	1.770096308	2.310095918
PB25	MBD+	Nuclear	0.001127	0.001213	0.001249	1.862	0.001284	0.00137	0.038317	0.040408	0.041345	0.937	0.042275	0.044783	0.888648723	0.962726934	0.993153667	1.024504141	1.105907713
PB25	MBD-	Nuclear	0.002173	0.002281	0.002326		0.002369	0.002469	0.036373	0.03802	0.038727		0.039478	0.041356	1.33517448	1.436037041	1.478849016	1.524740071	1.640955031
PB44	MBD+	Nuclear	0.000651	0.000846	0.000923	5.738	0.001002	0.001189	0.255458	0.261165	0.263656	1.106	0.266064	0.272096	1.053613393	1.083598461	1.095781154	1.108552265	1.139954991
PB44	MBD-	Nuclear	0.005129	0.005243	0.005294		0.005342	0.005456	0.283705	0.289151	0.291501		0.293776	0.299513	0.912835452	0.933094001	0.941680724	0.950363832	0.971165545
PB9	MBD+	Nuclear	0.002209	0.002354	0.002409	2.019	0.002466	0.002602	0.081474	0.083696	0.084689	1.242	0.085644	0.088144	1.436638889	1.503781363	1.529755961	1.556082532	1.624289959
PB9	MBD-	Nuclear	0.004647	0.004802	0.004865		0.004928	0.005078	0.101377	0.104013	0.105176		0.106349	0.109146	1.380228844	1.434871617	1.459876745	1.484918732	1.547565698
					AVERAGE	1.808					AVERAGE	1.175							
					MIN	0.880					MIN	0.788							
					MAX	5.738					MAX	1.751							

TableS4.4_Khroma DeltaS and Del

ID	Fraction	midDeltaD
MKb1_1	MBD+	0.003
MKb1_1	MBD-	0.010
MKb1_2	MBD+	0.007
MKb1_2	MBD-	0.013
MKb2_1	MBD+	0.008
MKb2_1	MBD-	0.013
MKb2_2	MBD+	0.010
MKb2_2	MBD-	0.013
MKhs_1	MBD+	0.003
MKhs_1	MBD-	0.007
MKhs_2	MBD+	0.007
MKhs_2	MBD-	0.009
MKm_1	MBD+	0.010
MKm_1	MBD-	0.009
MKm_2	MBD+	0.011
MKm_2	MBD-	0.011
MKs_1	MBD+	0.017
MKs_1	MBD-	0.014

KHROMA	Fraction	Average midDeltaD	midDeltaD Other tissue/ Bone
Muscle	MBD+	0.011	1.53
Skin	MBD+	0.017	2.43
Hair skin	MBD+	0.005	0.68
Bone	MBD+	0.007	
Muscle	MBD-	0.010	0.83
Skin	MBD-	0.014	1.12
Hair skin	MBD-	0.008	0.64
Bone	MBD-	0.012	
		min	0.64
		max	2.43
		average	1.20

ID	Fraction	midDeltaS
MKb1_1	MBD+	0.192
MKb1_1	MBD-	0.203
MKb1_2	MBD+	0.104
MKb1_2	MBD-	0.167
MKb2_1	MBD+	0.282
MKb2_1	MBD-	0.292
MKb2_2	MBD+	0.165
MKb2_2	MBD-	0.204
MKhs_1	MBD+	0.182
MKhs_1	MBD-	0.187
MKhs_2	MBD+	0.246
MKhs_2	MBD-	0.192
MKm_1	MBD+	0.184
MKm_1	MBD-	0.162
MKm_2	MBD+	0.192
MKm_2	MBD-	0.204
MKs_1	MBD+	0.201
MKs_1	MBD-	0.232

KHROMA	Fraction	Average midDeltaS	midDeltaS Other tissue/ Bone
Muscle	MBD+	0.188	1.01
Skin	MBD+	0.201	1.08
Hair skin	MBD+	0.214	1.15
Bone	MBD+	0.186	
Muscle	MBD-	0.183	0.84
Skin	MBD-	0.232	1.07
Hair skin	MBD-	0.189	0.87
Bone	MBD-	0.217	
		**min**	0.84
		**max**	1.15
		**average**	1.01

ID	Fraction	midDeltaS
MKb1_1	MBD+	0.192392
MKb1_1	MBD-	0.2034
MKb1_2	MBD+	0.104417
MKb1_2	MBD-	0.167561
MKb2_1	MBD+	0.28101
MKb2_1	MBD-	0.292206
MKb2_2	MBD+	0.165928
MKb2_2	MBD-	0.203263

KHROMA	Fraction	Average midDeltaS	midDeltaS bone 2 / bone 1	
MKb1	MBD+	0.148		
MKb2	MBD+	0.223	1.51	
MKb1	MBD-	0.185		
MKb2	MBD-	0.248	1.34	
		0.15	1.34	**min**
		0.25	1.51	**max**
		0.20	1.42	**average**

ID	Fraction	midDeltaD
MKb1_1	MBD+	0.002856
MKb1_1	MBD-	0.009645
MKb1_2	MBD+	0.006815
MKb1_2	MBD-	0.012694
MKb2_1	MBD+	0.008436
MKb2_1	MBD-	0.012957
MKb2_2	MBD+	0.009705
MKb2_2	MBD-	0.013378

KHROMA	Fraction	Average midDeltaD	
MKb1	MBD+	0.005	
MKb2	MBD+	0.009	
MKb1	MBD-	0.011	
MKb2	MBD-	0.013	
		0.005	**min**
		0.013	**max**
		0.010	**average**

ID	Fraction	midDeltaD
PB25	MBD+	0.001249
PB25	MBD-	0.002326
PB44	MBD+	0.000923
PB44	MBD-	0.005294
PB9	MBD+	0.002409
PB9	MBD-	0.004865

PB	Fraction	Average midDeltaD	
PB	MBD+	0.002	
PB	MBD-	0.004	
		0.002	**min**
		0.004	**max**
		0.003	**average**

TableS4.5_p-values

Specie	p-values DeltaD	p-values DeltaS	p-values Lambda
Ursus maritimus	8.6E-02	8.9E-01	7.4E-01
Equus caballus	6.9E-01	5.5E-01	9.4E-01
Mammuthus primigenius	4.0E-02	1.3E-01	3.3E-01

TableS5.1_dimers relative amoun

		Dimers relative abundancy																Relative abundancy of CG over:															
Sample	Library	AA	AC	AG	AT	CA	CC	**CG**	CT	GA	GC	GG	GT	TA	TC	TG	TT	AA	AC	AG	AT	CA	CC	**CG**	CT	GA	GC	GG	GT	TA	TC	TG	TT
EQ26	MBD+	0.035	0.057	0.071	0.034	0.078	0.082	**0.071**	0.071	0.061	0.102	0.083	0.058	0.023	0.062	0.079	0.036	1.997	1.250	0.995	2.098	0.905	0.866	**1.000**	0.996	1.153	0.695	0.854	1.217	3.120	1.147	0.893	1.979
EQ26	MBD-	0.075	0.055	0.077	0.080	0.078	0.044	**0.013**	0.077	0.067	0.047	0.044	0.055	0.068	0.067	0.078	0.075	0.175	0.241	0.172	0.164	0.169	0.298	**1.000**	0.172	0.197	0.282	0.297	0.240	0.195	0.198	0.168	0.175
EQ34	MBD+	0.037	0.055	0.073	0.036	0.077	0.077	**0.069**	0.070	0.063	0.097	0.081	0.060	0.024	0.063	0.079	0.039	1.841	1.249	0.944	1.904	0.892	0.891	**1.000**	0.976	1.093	0.705	0.841	1.144	2.816	1.085	0.867	1.743
EQ34	MBD-	0.067	0.055	0.080	0.071	0.081	0.050	**0.016**	0.080	0.068	0.053	0.050	0.055	0.057	0.068	0.082	0.067	0.237	0.291	0.200	0.225	0.197	0.320	**1.000**	0.199	0.235	0.299	0.320	0.289	0.279	0.236	0.195	0.237
EQ36	MBD+	0.039	0.058	0.071	0.038	0.079	0.076	**0.066**	0.070	0.063	0.093	0.078	0.060	0.025	0.064	0.080	0.040	1.692	1.141	0.929	1.746	0.833	0.862	**1.000**	0.938	1.048	0.709	0.843	1.094	2.632	1.028	0.827	1.651
EQ36	MBD-	0.073	0.055	0.078	0.077	0.080	0.046	**0.015**	0.078	0.067	0.050	0.046	0.054	0.062	0.067	0.080	0.072	0.202	0.269	0.187	0.191	0.183	0.317	**1.000**	0.189	0.219	0.294	0.316	0.269	0.236	0.220	0.184	0.203
EQA	MBD+	0.047	0.055	0.073	0.046	0.075	0.069	**0.058**	0.078	0.063	0.088	0.070	0.053	0.036	0.068	0.073	0.049	1.233	1.064	0.801	1.267	0.779	0.844	**1.000**	0.748	0.925	0.658	0.836	1.101	1.602	0.853	0.797	1.193
EQA	MBD-	0.066	0.055	0.079	0.068	0.081	0.053	**0.018**	0.080	0.068	0.055	0.053	0.055	0.055	0.068	0.080	0.066	0.267	0.318	0.222	0.260	0.219	0.330	**1.000**	0.221	0.260	0.319	0.331	0.318	0.320	0.259	0.219	0.266
HSSR	MBD+	0.062	0.054	0.078	0.063	0.081	0.058	**0.024**	0.079	0.067	0.063	0.057	0.054	0.048	0.067	0.081	0.063	0.386	0.447	0.307	0.380	0.296	0.416	**1.000**	0.305	0.361	0.380	0.419	0.444	0.496	0.358	0.295	0.384
HSSR	MBD-	0.063	0.054	0.080	0.065	0.083	0.057	**0.019**	0.080	0.067	0.061	0.056	0.054	0.049	0.067	0.083	0.063	0.294	0.344	0.233	0.286	0.222	0.327	**1.000**	0.232	0.278	0.305	0.328	0.343	0.375	0.277	0.222	0.292
MKb1_1	MBD+	0.062	0.056	0.080	0.057	0.073	0.059	**0.049**	0.070	0.073	0.070	0.059	0.054	0.048	0.066	0.068	0.056	0.796	0.878	0.609	0.856	0.674	0.825	**1.000**	0.698	0.671	0.704	0.824	0.902	1.018	0.740	0.724	0.875
MKb1_1	MBD-	0.074	0.056	0.078	0.074	0.081	0.047	**0.013**	0.078	0.068	0.047	0.047	0.056	0.060	0.068	0.081	0.074	0.180	0.235	0.171	0.180	0.164	0.284	**1.000**	0.171	0.196	0.280	0.284	0.235	0.221	0.196	0.164	0.180
MKb1_2	MBD+	0.065	0.056	0.081	0.061	0.072	0.057	**0.046**	0.069	0.074	0.065	0.057	0.055	0.051	0.066	0.067	0.059	0.711	0.827	0.570	0.759	0.639	0.809	**1.000**	0.672	0.624	0.712	0.813	0.845	0.902	0.700	0.691	0.789
MKb1_2	MBD-	0.082	0.055	0.075	0.082	0.078	0.042	**0.011**	0.075	0.067	0.042	0.042	0.056	0.068	0.067	0.078	0.082	0.136	0.200	0.148	0.135	0.143	0.266	**1.000**	0.148	0.166	0.267	0.266	0.200	0.163	0.166	0.143	0.136
MKb2_1	MBD+	0.061	0.059	0.074	0.061	0.076	0.055	**0.042**	0.073	0.067	0.064	0.056	0.059	0.052	0.066	0.075	0.060	0.682	0.701	0.564	0.675	0.547	0.749	**1.000**	0.572	0.618	0.648	0.745	0.707	0.801	0.626	0.551	0.692
MKb2_1	MBD-	0.076	0.056	0.077	0.078	0.079	0.045	**0.012**	0.077	0.066	0.045	0.045	0.056	0.066	0.066	0.080	0.076	0.155	0.210	0.154	0.152	0.149	0.261	**1.000**	0.154	0.178	0.263	0.261	0.210	0.180	0.178	0.148	0.155
MKb2_2	MBD+	0.064	0.060	0.073	0.067	0.076	0.051	**0.037**	0.072	0.068	0.057	0.051	0.060	0.056	0.067	0.076	0.065	0.568	0.611	0.499	0.549	0.483	0.717	**1.000**	0.505	0.542	0.637	0.713	0.608	0.648	0.544	0.484	0.567
MKb2_2	MBD-	0.080	0.056	0.076	0.081	0.078	0.043	**0.011**	0.075	0.066	0.042	0.043	0.056	0.069	0.066	0.078	0.080	0.137	0.194	0.144	0.133	0.139	0.254	**1.000**	0.144	0.164	0.257	0.254	0.193	0.158	0.164	0.139	0.136
MKhs_1	MBD+	0.057	0.058	0.073	0.055	0.074	0.061	**0.052**	0.072	0.066	0.075	0.063	0.056	0.046	0.064	0.073	0.056	0.917	0.902	0.712	0.951	0.705	0.850	**1.000**	0.725	0.782	0.689	0.821	0.931	1.136	0.809	0.715	0.924
MKhs_1	MBD-	0.067	0.057	0.079	0.066	0.082	0.054	**0.016**	0.078	0.067	0.053	0.054	0.057	0.054	0.067	0.082	0.067	0.240	0.282	0.205	0.243	0.197	0.299	**1.000**	0.205	0.239	0.302	0.298	0.281	0.295	0.239	0.197	0.240
MKhs_2	MBD+	0.063	0.057	0.074	0.061	0.074	0.057	**0.043**	0.072	0.067	0.067	0.058	0.056	0.052	0.066	0.072	0.061	0.683	0.759	0.584	0.704	0.587	0.763	**1.000**	0.599	0.648	0.649	0.748	0.766	0.830	0.656	0.601	0.707
MKhs_2	MBD-	0.074	0.057	0.077	0.074	0.079	0.048	**0.013**	0.077	0.067	0.047	0.048	0.057	0.061	0.067	0.079	0.074	0.177	0.231	0.171	0.177	0.165	0.272	**1.000**	0.171	0.196	0.281	0.271	0.230	0.215	0.196	0.165	0.176
MKm_1	MBD+	0.061	0.056	0.075	0.060	0.075	0.056	**0.043**	0.075	0.067	0.070	0.056	0.055	0.049	0.066	0.074	0.062	0.698	0.773	0.576	0.714	0.574	0.763	**1.000**	0.574	0.644	0.611	0.760	0.784	0.873	0.647	0.577	0.694
MKm_1	MBD-	0.077	0.054	0.078	0.074	0.079	0.045	**0.014**	0.078	0.069	0.047	0.045	0.055	0.058	0.068	0.080	0.078	0.177	0.251	0.175	0.185	0.172	0.302	**1.000**	0.176	0.199	0.288	0.304	0.249	0.234	0.200	0.172	0.175
MKm_2	MBD+	0.070	0.055	0.076	0.069	0.075	0.049	**0.029**	0.075	0.068	0.056	0.050	0.057	0.057	0.067	0.076	0.071	0.410	0.522	0.378	0.419	0.382	0.588	**1.000**	0.385	0.425	0.511	0.577	0.504	0.506	0.426	0.378	0.406
MKm_2	MBD-	0.085	0.054	0.075	0.082	0.077	0.040	**0.011**	0.075	0.069	0.042	0.040	0.054	0.065	0.068	0.077	0.085	0.132	0.210	0.150	0.138	0.147	0.279	**1.000**	0.151	0.164	0.271	0.279	0.210	0.173	0.167	0.146	0.133
MKs_1	MBD+	0.068	0.057	0.074	0.067	0.076	0.051	**0.034**	0.074	0.067	0.060	0.051	0.057	0.055	0.066	0.076	0.067	0.498	0.590	0.454	0.506	0.446	0.662	**1.000**	0.457	0.502	0.564	0.656	0.592	0.610	0.512	0.443	0.503
MKs_1	MBD-	0.074	0.056	0.077	0.073	0.081	0.047	**0.015**	0.077	0.067	0.049	0.047	0.056	0.058	0.067	0.080	0.075	0.206	0.275	0.199	0.210	0.191	0.326	**1.000**	0.199	0.228	0.311	0.327	0.276	0.264	0.228	0.192	0.206
MZh_1	MBD+	0.070	0.058	0.079	0.068	0.081	0.050	**0.018**	0.077	0.067	0.052	0.050	0.058	0.057	0.066	0.081	0.069	0.259	0.317	0.232	0.268	0.227	0.366	**1.000**	0.236	0.272	0.351	0.368	0.315	0.319	0.276	0.227	0.263
MZh_1	MBD-	0.072	0.056	0.077	0.073	0.080	0.050	**0.019**	0.075	0.065	0.052	0.049	0.055	0.061	0.066	0.077	0.072	0.269	0.347	0.253	0.267	0.244	0.391	**1.000**	0.258	0.300	0.371	0.396	0.354	0.319	0.295	0.253	0.268
MZl_1	MBD+	0.073	0.057	0.075	0.074	0.080	0.049	**0.017**	0.077	0.065	0.049	0.047	0.055	0.063	0.067	0.077	0.073	0.236	0.303	0.231	0.235	0.218	0.357	**1.000**	0.226	0.269	0.353	0.367	0.314	0.276	0.258	0.226	0.237
MZl_1	MBD-	0.076	0.060	0.074	0.080	0.080	0.046	**0.014**	0.078	0.063	0.044	0.043	0.054	0.072	0.067	0.074	0.075	0.179	0.226	0.184	0.169	0.170	0.293	**1.000**	0.175	0.215	0.309	0.316	0.251	0.189	0.203	0.183	0.182
MZm_1	MBD+	0.068	0.056	0.078	0.068	0.080	0.052	**0.017**	0.078	0.067	0.053	0.053	0.057	0.056	0.068	0.081	0.069	0.254	0.311	0.222	0.257	0.216	0.332	**1.000**	0.224	0.260	0.330	0.330	0.302	0.313	0.257	0.216	0.252
MZm_1	MBD-	0.069	0.059	0.076	0.068	0.082	0.052	**0.021**	0.077	0.066	0.055	0.051	0.056	0.056	0.066	0.080	0.067	0.299	0.350	0.269	0.302	0.251	0.396	**1.000**	0.265	0.312	0.373	0.406	0.366	0.366	0.310	0.257	0.307
PB25	MBD+	0.047	0.056	0.082	0.046	0.080	0.070	**0.039**	0.081	0.068	0.076	0.070	0.056	0.035	0.068	0.080	0.047	0.840	0.704	0.483	0.862	0.492	0.564	**1.000**	0.483	0.574	0.515	0.563	0.707	1.136	0.575	0.494	0.844
PB25	MBD-	0.076	0.055	0.077	0.079	0.077	0.047	**0.012**	0.077	0.067	0.045	0.047	0.054	0.066	0.067	0.077	0.075	0.163	0.225	0.159	0.155	0.159	0.263	**1.000**	0.159	0.182	0.273	0.263	0.226	0.185	0.182	0.160	0.163
PB44	MBD+	0.035	0.052	0.079	0.031	0.077	0.086	**0.063**	0.078	0.063	0.103	0.087	0.052	0.022	0.062	0.077	0.034	1.806	1.199	0.789	2.039	0.811	0.729	**1.000**	0.798	0.988	0.608	0.722	1.212	2.870	1.002	0.817	1.856
PB44	MBD-	0.061	0.055	0.083	0.062	0.082	0.058	**0.018**	0.083	0.069	0.057	0.058	0.055	0.049	0.069	0.081	0.061	0.297	0.331	0.220	0.295	0.223	0.316	**1.000**	0.220	0.264	0.317	0.316	0.333	0.370	0.264	0.224	0.298
PB9	MBD+	0.052	0.056	0.080	0.052	0.079	0.065	**0.037**	0.080	0.069	0.071	0.065	0.056	0.041	0.068	0.079	0.052	0.708	0.657	0.462	0.710	0.467	0.570	**1.000**	0.462	0.539	0.520	0.570	0.662	0.908	0.540	0.469	0.713
PB9	MBD-	0.079	0.054	0.076	0.082	0.077	0.045	**0.012**	0.076	0.067	0.043	0.045	0.054	0.069	0.067	0.076	0.078	0.153	0.221	0.158	0.146	0.157	0.269	**1.000**	0.158	0.179	0.279	0.270	0.222	0.174	0.180	0.158	0.154
																	**max**	3.120															

	p-values		
Dimer	*Equus caballus*	*Mammuthus primigenius*	*Ursus maritimus*
ApA	1.4E-03	3.8E-05	2.1E-02
ApC	2.0E-01	3.0E-01	9.7E-01
ApG	1.0E-01	3.4E-01	5.2E-01
ApT	1.3E-03	3.6E-05	2.4E-02
CpA	1.9E-01	5.3E-04	9.0E-01
CpC	1.7E-04	3.1E-04	4.4E-02
CpG	3.0E-05	1.6E-06	5.2E-02
CpT	1.3E-02	2.6E-03	6.3E-01
GpA	2.6E-03	3.6E-01	1.0E+00
GpC	1.7E-04	4.2E-05	5.3E-02
GpG	1.1E-04	2.2E-04	4.6E-02
GpT	1.7E-02	5.2E-02	7.0E-01
TpA	4.2E-03	4.0E-04	2.6E-02
TpC	2.0E-02	1.4E-01	1.0E+00
TpG	1.0E+00	5.2E-03	9.5E-01
TpT	9.3E-04	6.8E-05	2.2E-02

TableS6 MetaPhlAn

Supplementary Table S6. MetaPhlAn microbial DNA profiles.

Supplementary Table S7. Characteristics of the microbial DNA profiles.

Supplementary Table S8. Genome reference sequences of *Burkholderia* sp. used for alignment reads from ancient equid DNA extracts.

	EQA_MBD+	EQB_MBD+ … (remaining sample columns)
k__Archaea	0	[illegible]
k__Archaea|p__Euryarchaeota|c__Halobacteria	0	[illegible]
k__Archaea|p__Euryarchaeota|c__Halobacteria|o__Halobacteriales|f__Halobacteriales_unclassified	0	[illegible]
k__Archaea|p__Euryarchaeota|c__Methanomicrobia	0	[illegible]
k__Archaea|p__Euryarchaeota|c__Methanomicrobia|o__Methanomicrobiales	0	[illegible]
k__Archaea|p__Euryarchaeota|c__Methanomicrobia|o__Methanomicrobiales|f__Methanoregulaceae	0	[illegible]
k__Archaea|p__Euryarchaeota|c__Methanomicrobia|o__Methanomicrobiales|f__Methanoregulaceae|g__Methanoregulaceae_unclassified	0	[illegible]
k__Archaea|p__Euryarchaeota|c__Methanomicrobia|o__Methanomicrobiales|f__Methanoregulaceae|g__Methanosphaerula	0	[illegible]
k__Archaea|p__Euryarchaeota|c__Methanomicrobia|o__Methanomicrobiales|f__Methanoregulaceae|g__Methanosphaerula|s__Methanosphaerula_Methanosphaerula	0	[illegible]
k__Archaea|p__Euryarchaeota|c__Methanomicrobia|o__Methanosarcinales	0	[illegible]
k__Archaea|p__Euryarchaeota|c__Methanomicrobia|o__Methanosarcinales|f__Methanosarcinaceae	0	[illegible]
k__Archaea|p__Euryarchaeota|c__Methanomicrobia|o__Methanosarcinales|f__Methanosarcinaceae|g__Methanococcoides	0	[illegible]
k__Archaea|p__Euryarchaeota|c__Methanomicrobia|o__Methanosarcinales|f__Methanosarcinaceae|g__Methanococcoides|s__Methanococcoides_burtonii	0	[illegible]
k__Bacteria	100	[illegible]
k__Bacteria|p__Acidobacteria	0	[illegible]
k__Bacteria|p__Acidobacteria|c__Acidobacteria	0	[illegible]
k__Bacteria|p__Acidobacteria|c__Acidobacteria|o__Acidobacteriales	0	[illegible]
k__Bacteria|p__Acidobacteria|c__Acidobacteria|o__Acidobacteriales|f__Acidobacteriaceae	0	[illegible]
k__Bacteria|p__Acidobacteria|c__Acidobacteria|o__Acidobacteriales|f__Acidobacteriaceae|g__Granulicella	0	[illegible]
k__Bacteria|p__Acidobacteria|c__Acidobacteria|o__Acidobacteriales|f__Acidobacteriaceae|g__Granulicella|s__Granulicella_unclassified	0	[illegible]
k__Bacteria|p__Actinobacteria	37.49973	[illegible]
k__Bacteria|p__Actinobacteria|c__Actinobacteria	37.49973	[illegible]
k__Bacteria|p__Actinobacteria|c__Actinobacteria|o__Actinomycetales	37.49973	[illegible]
k__Bacteria|p__Actinobacteria|c__Actinobacteria|o__Actinomycetales|f__Corynebacteriaceae	0	[illegible]
k__Bacteria|p__Actinobacteria|c__Actinobacteria|o__Actinomycetales|f__Corynebacteriaceae|g__Corynebacterium	0	[illegible]
k__Bacteria|p__Actinobacteria|c__Actinobacteria|o__Actinomycetales|f__Corynebacteriaceae|g__Corynebacterium|s__Corynebacterium_accolens	0	[illegible]
[illegible]	[illegible]	[illegible]

TableS7 Microbial profiles

**Equid samples**[a]	**trimmed reads**	**mapped reads**[a]	**genera**	**Shannon diversity**[c]
EQA_MBD+	8894455	7303	5	1.43
EQA_MBD-	5776769	5683	9	1.72
EQB_MBD+	10136112	13338	6	1.46
EQB_MBD-	7000618	29875	10	1.47
EQC_MBD+	10509471	9397	7	1.59
EQC_MBD-	7065549	31402	15	1.56
EQE_MBD+	8435891	7598	10	1.91
EQE_MBD-	9195063	30192	12	1.49
EQF_MBD+	10060504	5788	8	1.74
EQF_MBD-	7550930	4076	12	2.03
**Kroma mammoth samples**[a]	**trimmed reads**	**mapped reads**[b]	**genera**	**Shannon diversity**[c]
MKb1_1_MBD+	10463207	3861	4	0.17
MKb1_1_MBD-	12872625	35883	6	0.05
MKb1_2_MBD+	2770052	4639	1	0
MKb1_2_MBD-	2385434	389	1	0
MKb2_1_MBD+	11880340	9435	6	0.89
MKb2_1_MBD-	11635626	13941	10	0.21
MKb2_2_MBD+	2989196	2883	3	0.09
MKb2_2_MBD-	2800110	733	2	0.51
MKhs_1_MBD+	11628153	8823	7	1.5
MKhs_1_MBD-	12680425	11795	16	1.33
MKhs_2_MBD+	2834480	2403	9	1.06
MKhs_2_MBD-	5239477	498	2	0.58
MKm_1_MBD+	11971341	14910	4	0.58
MKm_1_MBD-	16178263	36508	6	0.75
MKm_2_MBD+	3069468	7568	4	0.78
MKm_2_MBD-	15533439	22737	8	0.76
MKs_1_MBD+	12949713	17314	9	0.33
MKs_1_MBD-	20004888	35332	5	0.06
MKs_2_MBD+	2715457	4574	4	0.26
MKs_2_MBD-	2890744	2021	4	0.24
**Zhenya mammoth samples**[a]	**trimmed reads**	**mapped reads**[b]	**genera**	**Shannon diversity**[c]
MZh_1_MBD+	7171605	5557	9	1.47
MZh_1_MBD-	8067779	18631	9	1.08
MZh_2_MBD+	903182	2495	4	0.99
MZh_2_MBD-	2035979	339	2	0.68
MZl_1_MBD+	8702102	8180	9	1.26
MZl_1_MBD-	9004754	11227	16	1.81
MZl_2_MBD+	1901793	2654	11	1.65
MZl_2_MBD-	2516242	367	2	0.43
MZm_1_MBD+	10819017	8508	9	1.04
MZm_1_MBD-	13728406	21621	21	1.41
MZm_2_MBD+	3103006	6199	10	1.31
MZm_2_MBD-	2135223	555	2	0.69
**Polar bear samples**[a]	**trimmed reads**	**mapped reads**[b]	**genera**	**Shannon diversity**[c]
PB25_MBD+	2107980	1138	4	1.27
PB25_MBD-	1569088	1039	4	0.52
PB9_MBD+	1859912	1767	7	1.13
PB9_MBD-	1538441	2895	6	1.41

[a] "_MBD+" refers to the captured fraction, "_MBD-" refers to the uncaptured fraction; [b] to the MetaPhlAn database; [c] at the genus level.

TableS8 Burkholderia

Supplementary Table S6. MetaPhlAn microbial DNA profiles.

Supplementary Table S7. Ch		Number of non-equid mapped reads															
Accession n	Genome de	EQA_MBD+	EQA_MBD-	EQB_MBD+	EQB_MBD-	EQC_MBD+	EQC_MBD-	EQE_MBD+	EQE_MBD-	EQF_MBD+	EQF_MBD-	EQ26_MBD	EQ26_MBD	EQ34_MBD	EQ34_MBD	EQ36_MBD	EQ36_MBD-
Supplementar	Burkholderi	1724	1518	1508	3044	2398	5276	1950	4784	2820	5226	2944	108	1516	770	2736	568
CP001025.	Burkholderi	1732	1486	1488	2974	2312	5148	1936	4824	2678	5312	2860	96	1440	754	2530	540
CP000010.	Burkholderi	1308	1540	1174	2922	1806	5876	1590	5208	2028	5686	1912	88	1052	758	1610	652
CP000548.	Burkholderi	1286	1558	1158	2916	1796	5832	1582	5206	2034	5706	1906	86	1052	750	1618	648
CP000546.	Burkholderi	1282	1552	1152	2924	1800	5840	1570	5220	2020	5656	1902	86	1048	746	1614	642
BX571965.	Burkholderi	1308	1614	1110	2864	1682	5804	1470	5098	1912	5674	1864	68	1022	726	1602	606
CP000570.	Burkholderi	1274	1578	1080	2814	1626	5812	1432	5072	1904	5622	1866	74	1018	724	1600	594
CP000152.	Burkholderi	666	964	668	1696	1188	4364	964	3416	1350	3104	916	70	542	482	892	488
CP000441.	Burkholderi	612	824	652	1596	1158	3972	944	3104	1252	2800	908	66	520	468	848	494
CP000459.	Burkholderi	662	906	636	1684	1236	4296	992	3416	1342	2998	1008	56	536	452	980	500
CP003515.	Burkholderi	634	854	622	1492	1114	3914	894	3168	1196	2646	834	60	464	460	776	484
CP000869.	Burkholderi	610	880	610	1456	1160	3892	988	2856	1248	2644	914	58	514	418	890	444
CP000270.	Burkholderi	660	962	570	2058	846	4124	776	3722	1028	3430	634	40	350	586	622	458
CP002519.	Burkholderi	620	928	560	1870	776	3884	756	3428	942	3536	672	42	342	538	658	430
CP000573.	Burkholderi	586	872	538	1318	1044	4190	836	2952	1158	3072	716	64	396	436	644	504
CP002834.	Burkholderi	598	886	536	1310	1044	4206	818	2946	1156	3074	706	64	374	436	632	506
CP000125.	Burkholderi	596	894	534	1344	1060	4220	860	2960	1164	3072	722	64	392	436	654	504
CP000085.	Burkholderi	574	836	526	1272	1046	4022	840	2894	1126	2898	654	50	396	406	622	450
CP001043.	Burkholderi	566	882	520	1702	736	3444	624	3222	870	3232	656	36	378	482	622	402
CP000380.	Burkholderi	616	550	506	1112	990	2758	854	2266	1188	2278	802	62	454	362	734	412
CP003087.	Burkholderi	538	924	492	1972	754	4080	608	3634	888	3762	560	46	338	512	548	384
AP009387.	Burkholderi	386	360	410	752	770	2298	610	1580	852	1452	420	52	296	274	414	346
CP003775.	Burkholderi	384	736	388	1090	628	2520	496	2062	668	2060	670	24	276	254	580	220
CP000525.	Burkholderi	368	540	374	822	704	2252	536	1840	768	1886	484	30	258	264	444	288
CP000616.	Burkholderi	332	396	340	744	660	2464	582	1730	758	1586	312	54	238	314	340	358
CP003088.	Burkholderi	356	514	338	958	668	2892	564	2122	738	2008	314	44	220	344	334	364
CP000960.	Burkholderi	314	442	320	706	668	2322	574	1668	772	1570	386	46	234	302	376	336
AM747722.	Burkholderi	308	352	306	678	650	2130	552	1600	724	1356	346	46	248	274	384	342
CP002600.	Burkholderi	298	946	298	1092	460	3300	322	2376	494	2678	238	20	156	330	178	266
CP003089.	Burkholderi	292	394	294	788	596	2402	524	1862	672	1496	252	46	196	266	282	320
CP000545.	Burkholderi	294	566	282	666	418	2062	312	1450	480	1768	378	14	178	190	322	184
CP001504.	Burkholderi	320	750	282	858	428	2704	316	1942	434	2180	310	22	170	260	264	252
FR687359.	Burkholderi	252	502	242	848	306	1830	296	1608	384	1882	228	12	140	280	204	240
CP001053.	Burkholderi	208	436	198	712	216	1760	188	1422	240	1316	146	14	62	222	88	136
CP002218.	Burkholderi	146	338	122	554	146	1360	142	1058	200	1082	76	12	56	136	70	122
CP002520.	Burkholderi	168	362	120	568	174	1504	130	1194	186	1072	108	12	40	138	78	120
CP002015.	Burkholderi	48	174	52	266	50	714	34	578	66	496	28	2	18	60	16	52

In pink read counts for the genomes selected for k-mer analyses of the captured fractions (genomes against which the largest number of reads were aligned in the captured fraction (in green)).
In blue, read counts for the genomes selected for k-mer analyses of the uncaptured fractions (genomes against which the largest number of reads were aligned in the uncaptured fraction (in green)).

"CA", captured fraction; "UC", uncaptured fraction.
